# Assessing portfolio diversification via two-sample graph kernel inference. A case study on the influence of ESG screening

**DOI:** 10.1371/journal.pone.0301804

**Published:** 2024-04-16

**Authors:** Ragnar L. Gudmundarson, Gareth W. Peters

**Affiliations:** 1 Department of Actuarial Mathematics and Statistics, Heriot-Watt University, Edinburgh, United Kingdom; 2 Centre for Networks & Enterprise, Edinburgh Business School, Edinburgh, United Kingdom; 3 Department of Statistics & Applied Probability, University of California, Santa Barbara, Santa Barbara, California, United States of America; University of Kotli Azad Jammu and Kashmir Faculty of Management Sciences, PAKISTAN

## Abstract

In this work we seek to enhance the frameworks practitioners in asset management and wealth management may adopt to asses how different screening rules may influence the diversification benefits of portfolios. The problem arises naturally in the area of Environmental, Social, and Governance (ESG) based investing practices as practitioners often need to select subsets of the total available assets based on some ESG screening rule. Once a screening rule is identified, one constructs a dynamic portfolio which is usually compared with another dynamic portfolio to check if it satisfies or outperforms the risk and return profile set by the company. Our study proposes a novel method that tackles the problem of comparing diversification benefits of portfolios constructed under different screening rules. Each screening rule produces a sequence of graphs, where the nodes are assets and edges are partial correlations. To compare the diversification benefits of screening rules, we propose to compare the obtained graph sequences. The method proposed is based on a machine learning hypothesis testing framework called the kernel two-sample test whose objective is to determine whether the graphs come from the same distribution. If they come from the same distribution, then the risk and return profiles should be the same. The fact that the sample data points are graphs means that one needs to use graph testing frameworks. The problem is natural for kernel two-sample testing as one can use so-called graph kernels to work with samples of graphs. The null hypothesis of the two-sample graph kernel test is that the graph sequences were generated from the same distribution, while the alternative is that the distributions are different. A failure to reject the null hypothesis would indicate that ESG screening does not affect diversification while rejection would indicate that ESG screening does have an effect. The article describes the graph kernel two-sample testing framework, and further provides a brief overview of different graph kernels. We then demonstrate the power of the graph two-sample testing framework under different realistic scenarios. Finally, the proposed methodology is applied to data within the SnP500 to demonstrate the workflow one can use in asset management to test for structural differences in diversification of portfolios under different ESG screening rules.

## 1 Introduction

The ability to investigate the time-varying nature of portfolio diversification is fundamental to portfolio managers. In this work we seek to enhance the frameworks practitioners in asset management and wealth management may adopt when assessing how their investment decision-making may influence the portfolio diversification.

We consider a setting where an asset manager can select a total of *n* assets. However, due to considerations such as regulation, fund risk appetite, investor mandates on investment practice and scope of investment objects, etc., the fund manager has to select a subset of the *n* assets which satisfies these restrictions. The procedure we develop in this work, allows one to statistically test for differences in diversification and risk and return profiles of the obtained portfolio with respect to other possible protfolios. That is, the procedure allows for a robust testing and comparsion of various screening criteria or optimal investment strategies. This is achieved at the level of the inter-asset statistical return relationships rather than directly at the portfolio level, thereby also providing valuable interpretable insights into the resulting portfolio structures.

The problem of comparing screening rules and investment strategies arises in many investment contexts and we will illustrate this specifically in the timely and topical area of Environmental, Social, and Governance (ESG) based investing practices. For instance, take the iShares ETF investment platform, or the Vanguard platforms and look at the investment goals suggested by the robo-advisory. Each has some version that allows investors to select something akin to “go sustainable”, where in the case of the iShares platform, this selection directs the investor to a collection of iShares ETFs that have portfolios of screened assets that align with particular ESG objectives that include four broad categories: “Screened” which involves selections of ETFs that are designed to eliminate exposure to certain controversial business activities that pose risks or do not align with stated preferences.; “Broad ESG” which involves a selection of ETFs that invest in securities based on overall environmental, social, and/or governance (ESG) performance.; “Thematic ESG” involves a collection of ETFs that pursue specific environmental, social, governance, or Sustainable Development Goals (SDGs) issues.; and “Impact” which involves a collection of ETFs that intend to contribute to measurable positive environmental, social or SDG outcomes while pursuing financial returns.

The statistical frameworks developed in this manuscript will allow for the statistical testing of changes to diversification structures between portfolios constructed from different screening criteria, which enhances the growing literature on questions of ESG portfolio risk and return performance.

### 1.1 Motivation for studying portfolio diversification based on ESG screening

ESG investing is a broad field with many different investment approaches addressing various investment objectives across an array of equity and fixed-income markets. However, it is important to acknowledge that the field is still very much in its infancy and naturally this comes with many challenges that are yet to be resolved such as the basic question: Does ESG scores drive returns and value in the long run? The construction of ESG scores is in itself a challenging problem and consequently, there is an emerging literature highlighting challenges and issues that arise when developing coherent ESG scoring methods, see [1–5]. Nevertheless, sustainable investing is increasingly becoming central to capital allocation in many markets, as such the ESG metrics and scores have become of critical importance. Hence, in this work, we do not seek to enter the debate regarding the efficacy of ESG scores, as important as it is, as we feel that this area of investment practice is here to stay. We take this view in light of the significant infrastructure being built by Bloomberg, Moodys, Fitch, MSCI, Sustainalytics, etc. regarding measuring and monitoring ESG scores for all listed company stocks, warrants, depository receipts, ETFs, mutual funds, and the continued effort to start to add such ratings also in fixed income bond issuance’s. Therefore, whilst the form of the scoring methodology will undoubtedly evolve, the consideration of ESG scores and screening of investment assets by ESG scores will increasingly be relevant in investment decision making.

In this work, we seek to understand how to develop a statistically rigorous framework that allows one to test the effect ESG screening rules have on the risk profiles of portfolios, for example, does the diversification effects change? Such a framework, we believe, will be useful for practitioners who seek to form ESG factor investing-based models, ESG ETF funds, impact funds, and various other screened asset-traded instruments. Just as it is important to understand the role of counter-cyclical assets, growth assets, and defensive assets when forming an investment portfolio in the equity space, the new dimension of screening of assets by ESG scores may also influence the performance of the portfolio. This is a novel approach, as the focus of ESG studies has been on associations and possible causation between ESG scores and portfolio performance, see examples in [6–8].

In [7] they studied the link between ESG information and the valuation and performance of companies. This was achieved by examining a variety of transmission channels within a standard discounted cash flow model. Namely, they considered a cash-flow channel, an idiosyncratic risk channel, and a valuation channel. They argue based on the work of [9] that companies that possess a strong ESG profile are more competitive than their peers. In particular, their competitive advantage can be identified as arising from factors such as greater efficiency in the use of resources, enhanced human capital infrastructure, and better innovation management. Furthermore, they argue that companies that have better ESG ratings are typically better at developing long-term business plans and long-term incentive plans for senior management. They argue that not only do such companies often produce cashflows as dividends but that they are also considered growth stocks as they have a greater capacity to utilize their competitive advantage to generate abnormal returns, which ultimately leads to higher profitability. Consequently, higher profitability results in higher dividends. Evidence was provided for the validity of such economic channels in practice using the constituents of the MSCI ACWI Index, where the MSCI is a global equity index designed to represent the performance of the full opportunity set of large- and mid-cap stocks across 23 developed and 24 emerging markets. It is a good cross-section and highly representative as it covers more than 2,933 constituents across 11 sectors and approximately 85% of the free float-adjusted market capitalization in each market. The index is built using MSCI’s Global Investable Market Index (GIMI) methodology, which is designed to take into account variations reflecting conditions across regions, market cap sizes, sectors, style segments, and combinations.

Furthermore, [10–12] have studied the risk profiles of companies that have good ESG scores. For instance, they have shown that companies with strong ESG characteristics typically have above-average risk control and compliance standards. Consequently, they tend to suffer less frequently from severe incidents such as fraud, embezzlement, corruption, or litigation cases [9, 13]. As a result, they argue that with a reduction of such incidents there will be fewer stock-specific downside price pressures on the share price of companies. One could also argue that with less of such events, the operational risk capital that they may wish to set aside to mitigate such losses is reduced, freeing up such capital for more productive use in expanding the business. In [9] they again study such an economic channel in the MSCI ACWI constitute assets and show the relationship between each individual ESG characteristic of companies and how they are linked to stock price returns tail risks. They compare the residual volatility of companies across ESG quintiles, that is, the volatility that is not explained by the common factors in the MSCI Barra Global Equity Model. They also compared the kurtosis of stock returns across ESG quintiles; kurtosis is a commonly used measure for tail risks. They concluded that each of these stock-specific risk measures shows a lower idiosyncratic risk for high ESG-rated companies, in particular with respect to tail risks. Furthermore, such downside risks have been further studied in the pension fund context by [14].

When it comes to the question of individual company valuation and ESG ratings, [13, 15] demonstrated that the transmission channel from lower systematic risk to higher valuations can also be explained through the relative size of the investor base. They subsequently argued that companies with poor ESG ratings have a relatively small investor base since increasingly risk-averse investors are socially conscious and so will tend to avoid exposure to poorly ranked ESG rated companies, especially when they are aided by robo-advisory tools that provide them with guidance on such responsible investing screening of assets.

Whilst the highlighted studies above have elucidated the economic channels that link ESG scores to enhanced risk-return performance. It should be noted that there does exist a large literature on such studies and that the aforementioned works are just a small selection of the many works that have arisen from both in academia and the asset management industry. Challenges remain in the analysis undertaken in this domain, which has been exacerbated by two common causes: The different underlying ESG data used. There is still not a standardized and agreed-upon framework and methodology for how best to quantify ESG scores for companies and assets, see discussions in [16].; Secondly, many empirical studies analyzing the link between ESG and financial performance do not strictly differentiate between correlation and causality. With the exception of the aforementioned papers above, numerous works have estimated a correlation between ESG and financial variables and implicitly interpreted this to mean that ESG is the cause and financial value the effect, although the transmission easily could also be reversed. For instance, one can argue that companies with high ESG scores are better at managing their risks, leading to higher valuations. Alternatively, companies with higher valuations might be in better financial shape and therefore able to invest more in measures that improve their ESG profile; such investments might lead to higher ESG scores. Discussions on such challenges in this literature have been undertaken on a large scale through several meta-studies that have summarized the results of over 1,000 research reports and found that the correlation between ESG characteristics and financial performance was inconclusive. The existing literature found positive, negative, and nonexistent correlations between ESG and financial performance, although the majority of researchers found a positive correlation, see further discussions in [17, 18].

Finally, it has been argued that responsible ESG practices might mitigate the tail risk of a company meaning that they can reduce the left-tail risk of companies and, therefore, reduce ex-ante expectations of a left-tail event [19, 20]. Furthermore, good ESG scores are said to reduce the probability of an adverse event occurring and essentially reduce expected litigation costs, reputation losses, and environmental hazards [21, 22]. The impact of carbon risk on stock pricing has been investigated by [23, 24] where a brown-minus-green (BMG) risk factor is developed. [25] predict the accuracy of main financial indicators using ESG indicators. [20] used a R-vine copula to model the (tail-)dependencies of assets using ESG information. It is apparent that ESG factors have made the investment process noticeable more complex and while most empirical studies have been performed to analyze the interplay of ESG factors and financial performance, little has been done to test structural changes in the financial market due to ESG factors [26] across a large collection of screened assets say from the S&P500 universe of 500+ assets which are all ESG rated.

From the actuarial perspective, there is also activity taking place in the profession to explore the role of ESG based investing practice and the role of environmental practice in asset management, see for instance the Institute and Faculty of Actuaries (IFoA) who have initiated a working party on sustainability research, including: Net zero and the implications for investment portfolios, climate change disclosures, managing sustainability in the absence of metrics and measurements, and asset management. This demonstrates the fact that the actuarial community puts a great emphasis on developing adequate methods to measure and address the difficult subject of social responsibility.

### 1.2 Contributions

As outlined above current studies have largely focused on marginal relationships between a company’s risk-return profile and its ESG ratings. In this work, we focus on a different aspect of the problem. We study the structural relationships between assets and portfolio diversification structures that arise when various ESG based screening rules are applied to select assets for investment consideration in a portfolio strategy. This allows us to consider addressing formally and rigorously in a statistical framework questions such as: Does ESG screening of assets influence the diversification structure of a market or a portfolio, ETF, or other such targeted investment strategies? Furthermore, which components are affected most by ESG screening criteria, eg. sectors, industries, defensive stocks, counter-cyclical stocks?

In order to study such an aspect rigorously, we develop a statistical testing framework utilizing a kernel graph two-sample testing framework. This research is related to risk analysis within the ESG verse. We introduce a method that complements risk modeling at the portfolio level, by allowing for robust testing of structural changes in dependency structures in assets within a portfolio under different screening rules, our screening rules are based on ESG scores. This is achieved by using a graph testing framework that will be described in detail below. We further note that graph testing can also be used used to test structural changes in dependencies of financial markets after an intervention event should one also seek a causal analysis study.

Specifically, this paper makes the following contributions: An extensive statistical analysis is undertaken to list the expressiveness of various graph kernels for various graph structures using synthetic case studies. We consider cases, where traditional graph hypothesis settings fail such as where the nodes and/or edges are attributed, are labeled, and where the graph topology can be binary or weighted. For some kernels, we furthermore discuss why and in which setting they will give good or bad performances. We present a real-world application in risk management by showing how graph kernel two-sample testing can be used to compare financial graphs (portfolios). The method provides a statistical test to quantify whether two portfolios are the same or not, and can furthermore, be easily extended to change point detection analysis.

### 1.3 Notation

We use the following notation: Small boldface letters will denote a vector of observations and capital boldface letters will denote matrices. Capital letters (non-bold) can denote sets or random variables and should be clear from the context. *G* denotes a graph for consistency to other papers, if *G* denotes a random graph then we will underline the fact using G∼P where P is a probability distribution. *E* is a set of edges and *V* is the set of vertices. We also write *E*(*G*) and *V*(*G*) to denote the edge and node set of graph *G*. **A** is the adjacency matrix of a graph and bold upper case letters will denote matrices. Ω will denote a space containing graphs. H is a Hilbert space, and $H_{1}$ is the unit ball in a Hilbert space. (⋅, ⋅) is a kernel function where ⋅ represents a input. $\left|\right|\cdot {\left|\right|}_{H}$ will denote a norm with respect to the metric (Hilbert) space H and $<\cdot {>}_{H}$ is a dot product defined on the space H. 1: *M* is the set {1, …, *M*} where *M* is an integer. If *S* is a set then |*S*| denotes it cardinality. F will denote a *σ*-algebra, Q is a probability distribution. ⊗ is the Kronecker product.

### 1.4 Software for reproducible results

In undertaking this study, the graph kernels are calculated using the GraKel package [27] with the exception of the WWL and RW kernel choices. The WWL kernel was calculated using the original code from [28]. The RW code was written from scratch using the ideas from [29]. The code to calculate the robust MMD estimator was taken from [30] but adjusted to allow for different sample sizes. The Huge package was used for graph construction [31]. The code repository for this paper can be found https://github.com/ragnarlevi/MMD_Graph_Diversification.

## 2 Methodology

Whilst hypothesis testing and inference procedures are well established in standard Euclidean domains, there is a pressing need to develop families of inference procedures that will facilitate the development of testing frameworks when working with the topologies of graphs. This is particularly relevant as there is a growing literature that is developing graph and network-based statistical models for financial risk analysis and insurance modeling, see [32, 33].

There are well-established techniques being developed for transforming complex data structures into graph data embeddings, examples include obtaining samples of graphs independently, as observed sub-graphs/communities in a larger graph over time or as sub-graphs in a larger graph, see [34, 35]. Alternatively, one can use graph construction methods such as graph lasso methods to construct graph valued data, see [36]. Furthermore, substantial work has been performed on graph estimation from correlation matrices, especially in the financial research area. The main methods used are graphical lasso types of estimation [37–40], Laplacian estimation [41] or threshold rules [42, 43]. In this paper, we use the graphical lasso to estimate the precision matrix/graph of assets as it is a widely used covariance estimation tool that allows both positive and negative correlations.

However, having obtained such graph and network-structured data, there are far fewer studies on how to perform inference on such data structures. There has been some work on graph hypothesis testing, for example, [44] consider two-sample testing of large Erdös-Rényi graphs and proof asymptotic results both for single graph and samples of graphs. [45] present a framework based on matrix representation of networks and considers test statistics based on the matrix pairwise distances. [46] consider a two-sample hypothesis testing problem of undirected graphs where one has access to only one observation from each model. [47] consider two-sample testing of weighted graphs. [48] use a test statistic based on a similarity graph constructed on the pooled observations from the two samples. In this paper, we consider a kernelized version of graph sample testing. The main advantage of the kernelized graph testing framework is that it generalizes various graph testing frameworks by allowing a wide range of additional features such as node labels or attributes and edge labels or weights along with allowing different graph structures such as undirected, directed, bipartite, and weighted.

The work created in this manuscript takes a novel perspective on two sample testing for graph-valued data. A detailed development of kernel-based families of hypothesis testing frameworks for graph-valued data will be established. It will be demonstrated that such testing frameworks provide very flexible testing frameworks that can accommodate many types of graph testing structures of relevance to applications in financial risk and insurance modeling. The focus of the applications in this paper will be on Environmental, Social, and Governance (ESG) scoring methods and equity returns. This a topic of importance to all investment management communities, wealth management practitioners, pension funds, and endowments.

In formulating the concept of hypothesis testing on graph-based data. One can define many forms of inference questions and hypotheses to test based on specific structures of the graph data. The core focus of this work will be to explore two-sample hypothesis testing, effectively generalizing the classical notion of two-sample testing for distributions in Euclidean space to two-sample testing for distributions on graph structures via kernel two-sample testing. Such applications have relevance in addressing a multitude of inferential hypotheses.

In particular, the framework proposed in this manuscript will utilize the recently developed kernel two-sample testing framework introduced by [49] with an extension to graph-valued data samples through the development of various graph kernels. In the two-sample kernel testing methodology, one constructs two-sample testing by using the so-called maximum mean discrepancy (MMD) statistic. This has been shown to provide various forms of test statistics which have trade-offs between bias and computational evaluation efficiency, something that could be significant when testing collections of large graphs. Subsequent developments were made on kernel two-sample testing, where a statistic to account for autocorrelation was developed in [50] and robust methods were extended in [30]. Furthermore, there has been progress in forming estimates of the distribution under the null such as permutation, parametric, and eigenvalue methods see [49, 51].

The application of kernel MMD two-sample testing has to date focused on problems such as evaluating the performance of models [52, 53] and two-sample tests for nonstationary random processes [54]. [52] introduced a method to select kernels that maximize the power of a test. Additionally to the kernel MMD two-sample testing there exist kernel methods for measuring independence and conditional independence [55, 56]. It is not yet the case that such kernel testing methods have been extended to graph-valued data sets, this is therefore one of the novel contributions of the work undertaken in this manuscript.

To achieve the development of kernel two sample testing for graph data, we will explore various families of graph kernels. Graph kernels seek to quantify a notion of closeness or similarity between pairs of graphs, depending on the type of graph kernel used, one can define various notions of similarity between graphs at a macro scale and a micro vertex neighborhood scale. Graph kernels have mainly been used in graph classification using support vector machines and therefore they are benchmarked accordingly [57, 58]. This paper looks at a different approach and evaluates the performance or the expressiveness of kernels on various different graph structures. To do so we carefully perform various experiments and identify the best kernels to be used for a given graph structure. The graph structures tested are binomial graphs, scale-free graphs, stochastic block graphs, weighted graphs, node-labeled graphs, node-attributed graphs, and signed graphs. All these various types of graphs represent different types of graph-valued data sets that arise in applications in practice.

The majority of graph kernels are based on a convolution kernel [59] and there are multiple different graph kernels that exist. The Weisfeiler-Lehman kernel [60, 61] is one of the most used graph kernels and furthermore acts as a building block for other kernels. It utilizes the Weisfeiler-Lehman (WL) algorithm and can be used on node-labeled graphs. Random walk kernels are popular kernels [62–64] that can be used on undirected, directed, labeled, and edge-labeled graphs. Their computation efficiency is generally low, making the use of such graph kernels slow when large collections of graph-valued data are being studied or when the graph-valued datum involves very large numbers of vertices. However, recently [29] introduced a fast computation for the case of the geometric graph random walk kernel. The shortest path kernel [65] is a kernel that measures and compares the shortest path of graphs. It can be used for node-labeled graphs and attributed graphs, the computation is however slow. The Wasserstein Weisfeiler-Lehman graph kernel is based on the WL algorithm and can be used on node-labeled graphs [28]. It measures the distance between Weisfeiler–Lehman-inspired embeddings using the Wasserstein distance. Propagation kernels are based on monitoring how information spreads through a set of given graphs [66]. It can be used on directed, node-labeled, and attributed graphs. The pyramid match kernel can be used on graph samples with or without node labels [67, 68]. The WL optimal assignment kernel can be used on node-labeled graphs [69], where it has been proven that an optimal assignment kernel can be derived from the WL algorithm. Finally, we have the baseline kernels, the vertex histogram, and the edge histogram, which are simply counting how often a node label or an edge label occurs, respectively. It should be noted that many more graph kernels exist, for example, see [57, 58].

## 3 Graphs hypothesis testing

In this section, we will begin by defining the concept of graph-valued data and its various forms and characteristics that make it distinct from classical ways to encode data for financial data analysis and insurance and risk modeling applications. This will also serve to introduce some core notations and concepts used in later methodological developments for two sample graph testing frameworks.

### 3.1 Graphs valued data descriptors

In order to proceed with developing the concept of graph kernels and graph-valued data two-sample testing, we must first begin by defining a graph data point (i.e. a sample datum that is a graph) along with some of the characteristics of such a datum that will be useful to define the graph kernels that will measure similarity between data points (i.e. pairs of graphs).

**Definition 3.1** (Graph). *A graph is a pair G* = (*V*, *E*) *where V is a set of n vertices or nodes V* = {*v*_1_, …, *v*_*n*_} *and E is a set of edges E* ⊂ *V* × *V*.

The size of the graph is characterized by the number of nodes within the graph, denoted throughout by *N* = |*V*|, and the structure of the graph is characterized by the density which relates to the number of edges, denoted by *M* = |*E*|. Furthermore, a graph *G* is termed undirected if (*v*_*i*_, *v*_*j*_) ∈ *E* ⇔ (*v*_*j*_, *v*_*i*_)∈*E*. We sometimes write *V*(*G*) and *E*(*G*) to denote the node and edge set of a graph *G* respectively. One way to characterize a graph is via its adjacency matrix **A**, see Definition 3.2.

**Definition 3.2** (Adjacency Matrix). *The adjacency matrix* **A** *of a unweighted graph is*:

$$\begin{array}{c} A_{ij}=\{\begin{array}{cc} 1 & if(v_{i},v_{j})\in E\\ 0 & if(v_{i},v_{j})\notin E. \end{array} \end{array}$$

*The adjacency matrix* **A** *of a weighted graph is*:

$$\begin{array}{c} A_{ij}=\{\begin{array}{cc} w_{ij} & if(v_{i},v_{j})\in E\\ 0 & if(v_{i},v_{j})\notin E, \end{array} \end{array}$$

*where* $w_{ij}\in R$.

The local structure of a given sample graph datum provides a lot of information about the bigger structure of the graph. The adjacent nodes or the neighborhood of the *i*-th node (*v*_*i*_) provides a lot of information about the node itself and this fact is used in node classification and graph kernel construction. Formally, we can define the notion of a node or vertex neighborhood.

**Definition 3.3** (Neighborhood of a Node). *The neighborhood of a node v*_*i*_ *is denoted as* $N(v_{i})$ *and is the set of all vertices adjacent to v*_*i*_:

$$\begin{array}{c} N\left(v_{i}\right)=\left\{v_{j}:\left(v_{i},v_{j}\right)\in E\right\}. \end{array}$$

One of the most important node statistics often used to summarise graph-valued sample data, is the degree of a graph *G*, which described the significance of each node in the graph in terms of its connectedness to other vertices or nodes.

**Definition 3.4** (Node Degree). *The degree of an undirected graph G is*:

$$\begin{array}{c} deg\left(v_{i}\right)=k_{i}=|\left\{v_{j}:\left(v_{i},v_{j}\right)\in E\right\}|=|N\left(v_{i}\right)|. \end{array}$$

*The in-degree of a directed graph G is*:

$$\begin{array}{c} {deg}_{in}\left(v_{i}\right)=k_{i}^{in}=\left|\left\{v_{j}:\left(v_{j},v_{i}\right)\in E\right\}\right|. \end{array}$$

*The out-degree of a directed graph G is*:

$$\begin{array}{c} {deg}_{out}\left(v_{i}\right)=k_{i}^{out}=\left|\left\{v_{j}:\left(v_{i},v_{j}\right)\in E\right\}\right|. \end{array}$$

Furthermore, some graph data will have an additional structure such as labels associated with the vertices or nodes as well as labels potentially also associated with its edge set. These labels can give substantial information related to encoding the semantics of complex objects.

**Definition 3.5** (Labeled Graph). *A labeled graph G is endowed with a function l*: *V* ∪ *E* ↦ Σ *that assigns labels to the vertices and/or edges of the graph from a discrete set*, Σ, *of labels, called the alphabet. If the labeling function only labels nodes then the graphs are called node-labeled graphs and if the labeling function only labels edges then the graphs are called edge-labeled graphs*.

Note that there can be two labeling functions, one for the edges and one for the nodes with two distinct alphabets. Similar to labels, one may often encounter graph data sets in which each graph sample has nodes or edges that have an associated real-valued vector or matrix, usually called the node attributes.

**Definition 3.6** (Attributed Graph). *An attributed graph G is endowed with a function* $a:V\cup E\mapsto R^{d}$ *that assigns real-valued attributes to the vertices and/or edges*.

Other considerations one must think about when developing a graph kernel is whether it will focus on local vertex neighborhood structures or on global structures encompassing the entire graph when developing measures of similarity. In this vane, for the microlocal structure setting one can focus on the neighborhood of a collection of nodes or the community they live in rather than the whole graph itself. These communities are called sub-graphs and are often used when developing measures of similarity in graph kernel constructions or community analysis.

**Definition 3.7** (Sub-Graphs). *Let S be a set of vertices S* ⊂ *V*. *Then, G*[*S*] = (*S*, *E*[*S*]) *is the subgraph induced by S where E*[*S*] *is the set of edges that have both end-points in S*:

$$\begin{array}{c} E\left[S\right]=\{\left(v_{i},v_{j}\right):v_{i},v_{j}\in S\}. \end{array}$$

Besides the adjacency matrix **A** it is oftentimes convenient to work with the Laplacian matrix **L**. The Laplacian is of great importance in financial applications, where it is often assumed to encode the precision matrix associated with financial networks and is therefore used in the objective function and constraints in network estimation problems, see [41].

**Definition 3.8**. *Let* **A** *be the adjacency matrix of an undirected graph* **G** *and let* **D** *be a diagonal matrix with the degree of each node on the diagonal, D*_*ii*_ = ∑_*j*_ *A*_*ij*_. *Then the Laplacian* **L** *is*:

$$\begin{array}{c} L=D-A. \end{array}$$

Having presented some core components required to develop the graph two sample testing methodology presented in this paper, we can now proceed to introduce the framework.

### 3.2 Inference procedures for graph valued data: Two sample testing

We now aim to explore the notion of a distribution taking support on graphs. This is important to consider as when we discuss two sample testing for graph-valued data, we are effectively exploring either equivalence between two families of population distribution or their properties such as moments, cumulants, etc. As such it will be meaningful to recall the definition of a random graph in the context of two sample testing.

**Fig 1:**
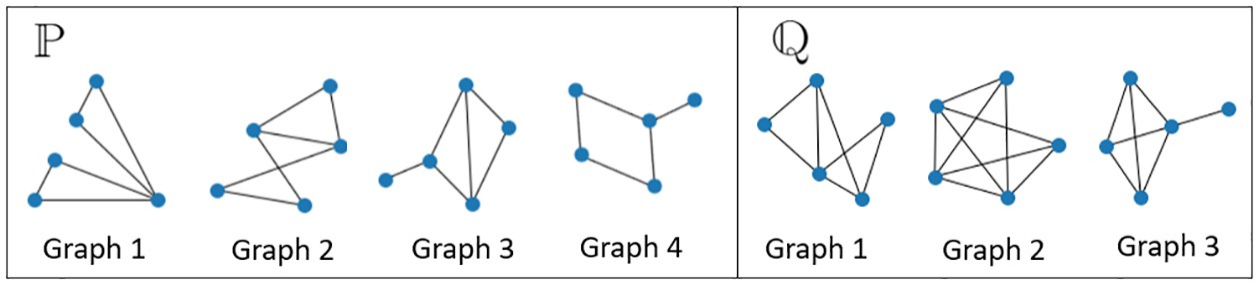
Graph two sample testing. The graph two sample testing scenario. Here we have observed 4 graphs from P and 3 graphs from Q. The sample space of P and Q is the same ((52) possible edges).

In the two-sample testing of graph-valued data we will explore, we assume we are given two sets of samples/observations that comprise collections of graph-valued data {*G*_1_, …, *G*_*n*_} and $\left\{G_{1}^{\prime },\ldots ,G_{n^{\prime }}^{\prime }\right\}$ where $G_{i},G_{j}^{\prime }\in \Omega ,\;\forall i,j$. The graphs in the two samples are all generated independently from two probability spaces (Ω,F,P) and (Ω,F,Q), and the goal is to infer whether P=Q. A visualization of the problem is given in Fig 1.

It is worth pausing for a moment to inspect the probability spaces more closely. In the simplest case, the sample space Ω contains all possible edges that can occur in a graph *G*, that is Ω = {(*v*_1_, *v*_2_), …, (*v*_1_, *v*_|*V*|_), (*v*_2_, *v*_1_), …, (*v*_|*V*|_, *v*_|*V*|−1_)} (We are assuming that a node can not be connected to itself). As the sample space is discrete we can define the *σ*-algebra as the power set of Ω, namely, F=P(Ω). The probability function P:F↦[0,1] then defines the probability of obtaining a certain graph in the sample set of graph-valued data. As an example we can define for instance a population distribution to be uniform P(G(|V|,|E|))=1(M|E|) where M=(|V|2) is the total number of possible edges and *G*(|*V*|, |*E*|) is a graph with |*V*| vertices and |*E*| number of edges.

Now, returning to the concept of two sample testing for graph valued data. The goal is to infer whether the two samples of graphs are generated according to the same distribution. This involves developing a statistical test $T({\left\{G\right\}}_{i=1}^{n},{\left\{G^{\prime }\right\}}_{i=1}^{n^{\prime }})$ to determine from the population samples whether there is sufficient evidence to reject a null that both population distributions generating the two samples of graphs are equivalent, where $T\left({\left\{G\right\}}_{i=1}^{n},{\left\{G^{\prime }\right\}}_{i=1}^{n^{\prime }}\right):{\left\{G\right\}}_{i=1}^{n}\times {\left\{G^{\prime }\right\}}_{i=1}^{n^{\prime }}\mapsto \left\{0,1\right\}$ is a function that distinguishes between the null hypothesis and the alternative hypothesis:

$$\begin{array}{c} \begin{array}{cc} H_{0}: & P=Q\\ H_{1}: & P\neq Q. \end{array} \end{array}$$ (3.1)

As in classical inference procedures, one can directly employ the ideas from standard inference to graph-valued testing settings. The test (3.1) is the main theme of this study. A type *I* error is made when P=Q is rejected when *H*_0_ is true and a type *II* error is made when P=Q is failed to be rejected when *H*_1_ is true. The level *α* of a test is the upper bound of a Type *I* error and is a parameter decided beforehand. The power of a test is the probability that the test correctly rejects *H*_0_ when *H*_1_ is true. A consistent test achieves *α* and a type *II* error of zero when the samples approach infinity.

When constructing graph sample tests one is required to define some kind of features that summarize the graphs within the sample. This will be used to make an inference or seek empirical evidence to reject a null statement that the data-generating population distributions for each graph-valued sample set are equivalent.

In this context, one can choose to map from graph-valued data to summary statistics of each graph data point and then seek statistical evidence that such features are sufficiently different so as to reject a null that the two graph samples were from the same population. In this regard, one could resort to summary statistics such as the average of the average degree of the graphs or the average of the shortest paths. This, however, might be too simple a measure for graphs as they can have rich features which are not captured by these single summary statistics. The challenge with this approach is that the summary statistics may not characterise sufficiently the features of the graph distribution, such basic summaries will in general not act as sufficient statistics, the consequence is then to have a less powerful test for the decision to be made as to reject the null or not given two samples of graphs.

Instead, to preserve the power of testing such a hypothesis we advocate in this paper for the use of graph kernels, which utilize the entirety of the graph-valued data samples, rather than just crude summary statistics when forming the test statistic. This however comes with some level of complexity as one must now define a mechanism to measure the similarity between pairs of graphs from each population sample. This is where kernel methods come into play [70, 71]. In this paper, we are interested in computing the similarity between two graphs *G* and *G*′ by computing their similarity in a reproducing kernel Hilbert space (RKHS) H.

### 3.3 Graph valued data embedding to kernel RKHS space

The main idea of the kernel method is that if two functions f∈H,g∈H are close in the RKHS H then *f*(*G*) and *g*(*G*′) are close for all *G* and *G*′ that are close, such that *G*, *G*′ ∈ Ω where Ω is as space of graphs. This fact is only true if the space is a so-called reproducing kernel Hilbert space (RKHS) space.

We begin by introducing a special function in H, namely a function that assigns to each f∈H its value at *G* ∈ Ω which plays an important role in the theory of RKHS:

**Definition 3.9** (RKHS). *Let* H *be a Hilbert space of functions* f:Ω→R. *Consider the linear functional over the space of functions in* H *that evaluates each function at a point G*,

$$\begin{array}{c} \delta_{G}:f\mapsto f\left(G\right),\forall f\in H. \end{array}$$

*If for all G*, *δ*_*G*_ *is continuous at any* f:Ω↦R, *or equivalently, δ*_*G*_ *is bounded. Then the Hilbert space of functions* H *is called a reproducing kernel Hilbert space (RKHS)*.

Since the evaluation functional *δ*_*G*_ is linear and bounded/continuous we have by the Riesz representation theorem [72] that there exists an element/function $k_{G}\in H$ such that $\delta_{G}\left(f\right)=f\left(G\right)={\langle f,k_{G}\rangle }_{H}$. *k*_*G*_ is called the reproducing element.

**Definition 3.10** (Reproducing Kernel). *Let* H *be a Hilbert space of functions* f:G↦R *and for any two graphs G and G*′ *such that G*, *G*′ ∈ Ω. *The kernel function measuring similarity between G and G*′ *through k*(*G*, *G*′) *defined by the function* k:Ω×Ω↦R *is called the reproducing kernel of* H *if it satisfies*:

- ∀*G* ∈ Ω, k(·,G)∈H,
- ∀*G* ∈ Ω, ∀f∈H, ${\langle f,k\left(\cdot ,G\right)\rangle }_{H}=f\left(G\right).\;$ (*reproducing property*)

When working with kernels the usual thing that is done is to use a feature mapping *k*_*G*_ = *ϕ*(*G*) (see definition 3.11). In particular it can be seen that as $k_{G},k_{G^{\prime }}\in H$ we have $k\left(G,G^{\prime }\right)=<k\left(\cdot ,G\right),k\left(\cdot ,G^{\prime }\right){>}_{H}$. In our case, we are mapping each *G* to a function that is a linear combination of kernels within H. The kernel function is defined as:

**Definition 3.11** (Kernel Function). *A kernel is a positive definite function k such that for all graphs G*, *G*′ ∈ Ω

$$\begin{array}{c} k\left(G,G^{\prime }\right)=<\phi \left(G\right),\phi \left(G\right){>}_{H}, \end{array}$$

*where* ϕ:G↦H *is called the feature mapping*

When working with data we need to calculate the kernel function between all data points and store them in a matrix called the kernel matrix **K**.

**Definition 3.12** (Kernel Matrix). *The kernel matrix* $K\in R^{n\times n}$ *is defined as*:

$$\begin{array}{c} K_{ij}=k\left(G_{i},G_{j}\right), \end{array}$$

*where* ${\left\{G_{i}\right\}}_{i=1}^{n}$ *is the data*.

In the context of two sample graph testing, we have two data sources, sample 1 of graphs assumed drawn from P and sample 2 of graphs assumed drawn from Q. It can be good to order the kernel matrix such that **K** has a block structured as follows:

$$\begin{array}{c} K=[\begin{array}{cc} K_{PP} & K_{PQ}\\ K_{QP} & K_{QQ} \end{array}], \end{array}$$ (3.2)

where $K_{PP}$ is the Kernel function evaluated at data points within the sample coming from the unknown distribution P, $K_{QQ}$ is the Kernel function evaluated at data points within sample coming from the unknown distribution Q, and $K_{PQ}$ is the Kernel function evaluated at data points between the two samples. Note $K_{PQ}$ = $K_{QP}^{T}$.

Given this brief introduction to kernels, we can now introduce the kernel mean embedding. Assume that the sample ${\left\{G_{i}\right\}}_{i=1}^{n}$ was generated from a probability distribution P, meaning that each *G*_*i*_ is a realization of the random graph G∼P. The notion of feature maps for probability distributions is extended to the so-called embedding of a probability distribution [73] and is defined as follows:

**Definition 3.13** (The Mean Embedding). *The mean embedding* $\mu_{P}\in H$ *of the random variable K*(*G*, ⋅), *where G is a random graph with the law* P, *is defined as*:

$$\begin{array}{c} E_{G∼P}\left[f\left(G\right)\right]={\left\langle f,\mu_{P}\right\rangle }_{H}, \end{array}$$

*for all* f∈H.

The following lemma gives conditions such that the mean embedding exists given that the mean embedding $\mu_{P}$ exists see [49]:

**Lemma 3.1**. *If k*(⋅, ⋅) *is measurable and* $E_{G∼P}\left[\sqrt{k(G,G)}\right]<\infty$ *then* $\mu_{P}\in H$ *exists*.

If $\mu_{P}$ exists then we have, by the reproducing property and the definition of $\mu_{P}$, $\mu_{P}\left(G^{\prime }\right)=\langle \mu_{P},k\left(G^{\prime },\cdot \right)\rangle =E_{G∼P}\left[k\left(G^{\prime },G\right)\right]$. If P is known then it is sometimes possible to find the mean embedding $\mu_{P}$. As an example consider the kernel:

$$\begin{array}{c} k\left(G,G^{\prime }\right)={\left(b⊗b\right)}^{T}\left(A⊗A^{\prime }\right)\left(b⊗b^{\prime }\right), \end{array}$$

here the random variable is the graph *G* so the adjacency matrix **A** is random ***a***, ***a***′, ***b***, and ***b***^**′**^ are vectors (not random). The kernel is called a 1-step random walk kernel and will be covered more in-depth in a latter section. If we use the binomial graph measure where the probability of edges *p* is fixed:

P(G)=pm(1-p)(|V|2)-m,

then the mean embedding will be:

$$\begin{array}{c} \mu_{P}\left(G^{\prime }\right)=E_{G∼P}\left[{\left(b⊗b^{\prime }\right)}^{T}\left(A⊗A^{\prime }\right)\left(a⊗a^{\prime }\right)\right]={\left(b⊗b^{\prime }\right)}^{T}\left(E_{A∼P}\left[A\right]⊗A^{\prime }\right)\left(a⊗a^{\prime }\right), \end{array}$$

where $E_{A∼P}\left[A\right]$ is a matrix where each entry is *p* except at the diagonal where it is 0.

**Remark**. *Note that in this simple scenario there is a one-to-one correspondence between a graph and its adjacency matrix*.

### 3.4 Graph two sample test statistic: Maximum mean discrepancy

The maximum mean discrepancy (MMD) is a general class of operator that measures equivalence between population distributions when they are embedded into an RKHS space, which is then equivalent to measuring the supremum between the mean of functions in the RKHS with respect to each population distribution. Its properties also allow for an empirical formulation to be used where one replaces population distributions with empirical measure estimators obtained from samples drawn from each population distribution. This can then be used as the basis of a hypothesis-testing framework as will be outlined below.

In order to explain this in detail we first present the population distribution definition of the MMD statistic. Consider some function class F (which will be the unit ball in a RKHS space in our case) then the MMD is given in Definition 3.14.

**Definition 3.14** (MMD). *Let* F *be a class of functions* f:Ω→R. *The maximum mean discrepancy (MMD) is defined as*:

$$\begin{array}{c} \text{MMD}\left[F,P,Q\right]≔\underset{f\in F}{\text{sup}}\;\left(E_{G∼P}\left[f\left(G\right)\right]-E_{G^{\prime }∼Q}\left[f\left(G^{\prime }\right)\right]\right). \end{array}$$ (3.3)

If we let the function class $F=H_{1}={\left\{f:||f|\right|}_{H}=1\}$ be the unit ball in a reproducing kernel Hilbert space H characterized by reproducing kernel *k*(⋅, ⋅) (in our case between graph valued data pairs of points), then it is possible to derive an attractive test statistic based on kernel evaluations of elements in the two samples, given that the mean embedding $\mu_{P}$ exists [49]:

$$\begin{array}{c} \begin{array}{cc} {\text{MMD}}^{2}\left[H_{1},P,Q\right] & ={\left(\underset{f\in H_{1}}{\text{sup}}\;\left(E_{G∼P}\left[f\left(G\right)\right]-E_{G^{\prime }∼Q}\left[f\left(G^{\prime }\right)\right]\right)\right)}^{2}\\ & ={\left(\underset{f\in H_{1}}{\text{sup}}\;<\mu_{P}-\mu_{Q},f>\right)}^{2}\\ & =\left|\right|\mu_{P}-\mu_{Q}{\left|\right|}_{H}^{2}\\ & =<\mu_{P},\mu_{P}{>}_{H}+<\mu_{Q},\mu_{Q}{>}_{H}-2<\mu_{P},\mu_{Q}{>}_{H}\\ & =E_{G∼P}\left[\mu_{P}\left(G\right)\right]+E_{G^{\prime }∼Q}\left[\mu_{Q}\left(G^{\prime }\right)\right]-2E_{G∼P}\left[\mu_{Q}\left(G\right)\right]\\ & =E_{P,P}\left[k\left(G,G\right)\right]-2E_{P,Q}\left[k\left(G,G^{\prime }\right)\right]+E_{Q,Q}\left[k\left(G^{\prime },G^{\prime }\right)\right], \end{array} \end{array}$$ (3.4)

where G∼P and $G^{\prime }∼Q$ are random graphs. For the MMD to be a metric, some assumptions need to hold, see Proposition 3.2.

**Proposition 3.2**. *Let* $H_{1}$ *be a ball in a universal RKHS* H, *defined on the compact metric space* Ω, *with associated continuous kernel k. Then* ${MMD}^{2}\left[H_{1},P,Q\right]=0$ *if and only if* P=Q.

This verifies that one has a reference value of zero for the population-based metric under a null statement that both population graph measures are equivalent. Hence, if one can then find a means to estimate this MMD metic using samples from the population distributions, this could then form the basis of a test statistic for two sample testing. Fortunately, the next result demonstrates that such a sample-based estimator can be considered when estimating Eq (3.4), which is provably unbiased and given for two sets of *n* and *n*′ samples of graphs by:

$$\begin{array}{c} {\hat{\text{MMD}}}_{u}^{2}\left[H_{B},P,Q\right]=\frac{1}{n(n-1)}\sum_{i=1}^{n}\sum_{j\neq i}^{n}k\left(G_{i},G_{j}\right)+\frac{1}{n^{\prime }\left(n^{\prime }-1\right)}\sum_{i=1}^{n^{\prime }}\sum_{j\neq i}^{n^{\prime }}k\left(G_{i}^{\prime },G_{j}^{\prime }\right)-\frac{2}{nn^{\prime }}\sum_{i=1}^{n}\sum_{j=1}^{n^{\prime }}k\left(G_{i},G_{j}^{\prime }\right). \end{array}$$ (3.5)

The computational complexity of evaluating this statistic on two population samples of size *n* and *n*′ respectively is *O*((*n* + *n*′)^2^). This would be expensive if one had a large collection of graph-valued data samples and even worse if each sample was comprised of graphs with very large vertex set cardinality. However, there exists a linear time statistic that will be denoted by ${\hat{\text{MMD}}}_{l}^{2}$ which can be computed in *O*(*n*) time. Assume that *n* = *n*′ and define *n*_2_ = ⌊*n*/2⌋, where ⌊⋅⌋ is the floor function, then the linear estimate is computed as:

$$\begin{array}{c} {\hat{\text{MMD}}}_{l}^{2}\left[H_{B},P,Q\right]=\frac{1}{n_{2}}\sum_{i=1}^{n_{2}}\left(k\left(G_{2i-1},G_{2i}\right)+k\left(G_{2i-1}^{\prime },G_{2i}^{\prime }\right)-k\left(G_{2i-1},G_{2i}^{\prime }\right)-k\left(G_{2i},G_{2i-1}^{\prime }\right)\right), \end{array}$$

while ${\hat{\text{MMD}}}_{l}^{2}$ has higher variance than ${\hat{\text{MMD}}}_{u}^{2}$, it is computationally much more appealing.

### 3.5 Defining extreme outlier samples for graph valued data and robust MMD test statistics

**Fig 2:**
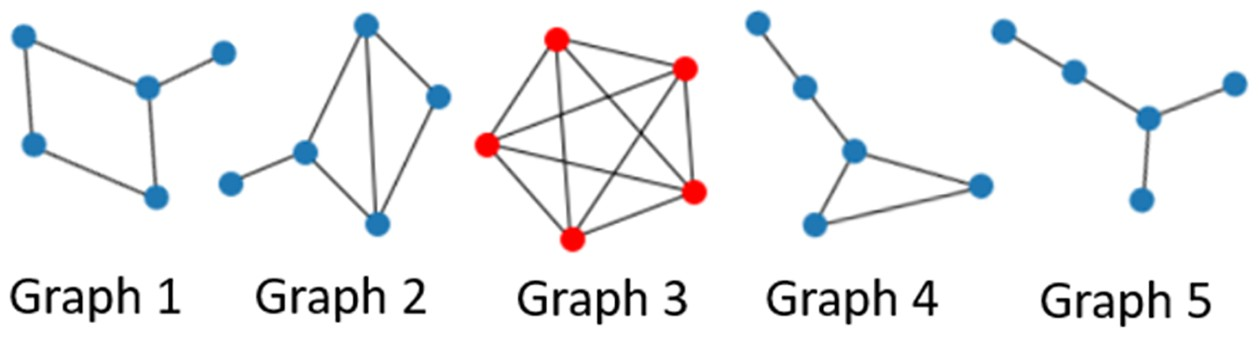
Outlier graph. A graph sample with an outlier graph (red) with respect to degree.

**Fig 3:**
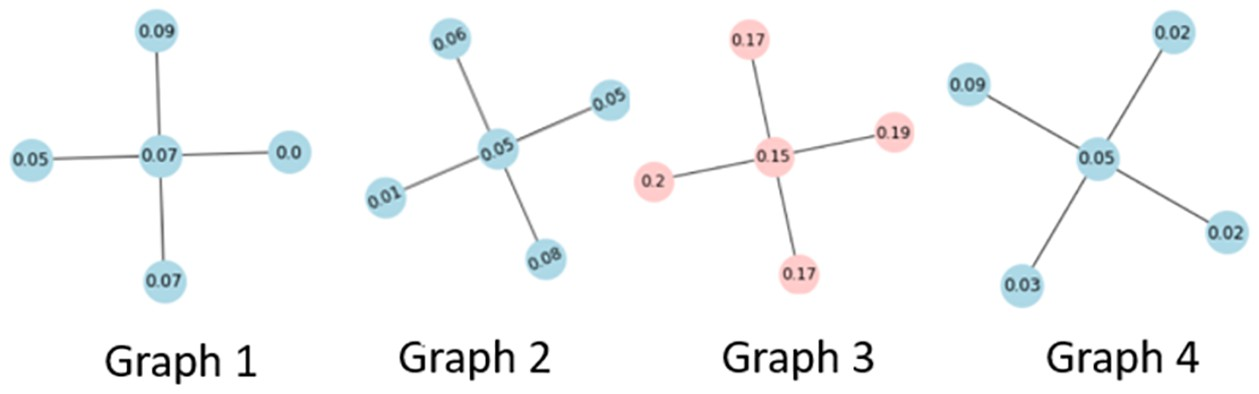
Outlier graph. A graph sample with an outlier graph (red) with respect to attributes.

Before we detail how to develop a robust version of the MMD statistic estimator. It is worth putting some consideration towards what exactly is an outlier or extreme sample when considering graph-valued sample data. In other words, what is meant by an outlier in a graph-valued data setting? In essence, there can be more than one type of outlier that can be observed when it comes to structured data as can be seen in Figs 2 and 3. In Fig 2 we can see that the graphs have an average degree of around 2, however, graph no. 3 is a complete graph and is clearly different from the rest of the graphs in the samples. This example is a realistic scenario in the context of the financial markets. Namely, in times of crisis, most financial assets show strong correlations while in a normal state the correlations are usually weaker and the resulting graphs have fewer connections. In Fig 3, we show another type of outlier, namely in the node attributes. Here the outlier is again graph 3 as the node attributes are significantly higher, even though the graph topology in terms of edge relations to vertices is common between all samples. This is the usual notation of an outlier in numerical data. Other types of outliers may happen as well such as the node label distribution, edge weights, or the number of triangles. If an outlier is present in the sample it can severely affect estimates that are not robust to outliers like the unbiased MMD estimate. Therefore, a robust version that replaces the expectation with the median of averages taken over non-overlapping blocks of the data has been developed [30].

In order to robustify the MMD statistic estimator, we may proceed as follows with the classical robust estimator one would adopt in Euclidean sample space settings, modified for the MMD setting for graph-valued data as follows. Consider the partition {*S*_*q*_}_*q* ∈ 1 : *Q*_ of |*S*_*q*_| = *N*/*Q* partitions. The empirical measure of sample *S*_*q*_ is, $P_{S_{q}}={\left|S_{q}\right|}^{-1}\sum_{i\in S_{q}}\delta \left(x_{i}\right)$, giving rise to the expectation $E_{P_{S_{q}}}\left[h\left(X\right)\right]={\left|S_{q}\right|}^{-1}\sum_{i\in S_{q}}h\left(x_{i}\right)$ where h∈H. The Median Of meaN (MON) is defined as:

$$\begin{array}{c} MON_{Q}\left[f\right]=med_{q\in 1:Q}\left\{P_{S_{q}}h\right\}=med_{q\in 1:Q}\left\{<h,\mu_{P_{S_{q}}}>\right\}. \end{array}$$

Implying that we are taking the median of the mean embeddings of each partition. The MON-based MMD estimator associated to kernel *k* (*MONK*) is then defined as:

$$\begin{array}{c} \begin{array}{cc} {\hat{MMD}}_{Q}\left(P,Q\right) & =\underset{f\in H_{1}}{\text{sup}}\;med_{q\in 1:Q}\{<f,\mu_{P_{S_{q}}}-\mu_{Q_{S_{q}}}{>}_{H}\}\\ & =\underset{f\in H_{1}}{\text{sup}}\;med_{q\in 1:Q}\{E_{G∼P_{S_{q}}}\left[f\left(G\right)\right]-E_{G^{\prime }∼Q_{S_{q}}}\left[f\left(G^{\prime }\right)\right]\}. \end{array} \end{array}$$

Replacing the population expectation with the empirical expectation we have:

$$\begin{array}{c} \begin{array}{cc} {\hat{MMD}}_{Q}\left(P,Q\right) & =\underset{f\in H_{1}}{\text{sup}}\;med_{q\in 1:Q}\{\frac{1}{|S_{q}|}\sum_{j\in S_{q}}f\left(G_{j}\right)-\frac{1}{|S_{q}|}\sum_{j\in S_{q}}f\left(G_{j}^{\prime }\right)\}. \end{array} \end{array}$$

By the representer theorem [74] we can express the optimal *f* as:

$$\begin{array}{c} f=\sum_{i}a_{i}k\left(\cdot ,x_{i}\right)+\sum_{i}b_{i}k\left(\cdot ,y_{i}\right), \end{array}$$

where $a_{i},b_{i}\in R$ are some constants. We next observe that by denoting $c=\left[a,b\right]\in R^{2n}$, $K=\left[K_{PP},K_{PQ};K_{QP},K_{QQ}\right]\in R^{2n\times 2n}$. We can rewrite the MON-based MMD estimator as:

$$\begin{array}{c} \underset{c:c^{T}Kc\leq 1}{\text{max}}\;med_{q\in 1:Q}\{\frac{1}{|S_{q}|}\left[1_{q},-1_{q}\right]Kc\}, \end{array}$$

where $1_{q}\in R^{n}$ is an indicator vector of block *q*. Note that we have **c**^*T*^**K** **c** ≤ 1 as we are searching for functions within the unit ball of H. This matrix objective function is then solved to find the best MON-based estimate. The key takeaway is that the number of corrupted samples, *N*_*c*_ can almost be half of the number of blocks, *Q*. That is, there exists *δ* ∈ (0, 1/2] such that *N*_*c*_ ≤ *Q*(1/2 − *δ*).

### 3.6 Hypothesis decision making

Given our MMD statistic on probability distributions, we would like to infer whether two sets of samples come from the same underlying distribution by determining whether the observed empirical MMD is within a reasonable decision region. Under the null $H_{0}:P=Q$, ${\hat{MMD}}_{u}^{2}$ converges asymptotically to a distribution that depends on the unknown distribution P[49]. This unfortunately means that we can not evaluate a closed-form decision threshold *c*_*α*_ and reject *H*_0_ if $n{\hat{MMD}}_{u}^{2}\left(P,Q\right)>c_{\alpha }$ where *α* is the level of significance of this test that represents the probability of a false rejection i.e. a Type II error corresponding to a false negative. Instead, we estimate a data-dependent threshold ${\hat{c}}_{\alpha }$ by using a permutation procedure that replaces the unknown population distribution P with a sampled empirical equivalent. The exact algorithm can be found in the accompanying technical S1 Appendix.

**Fig 4:**
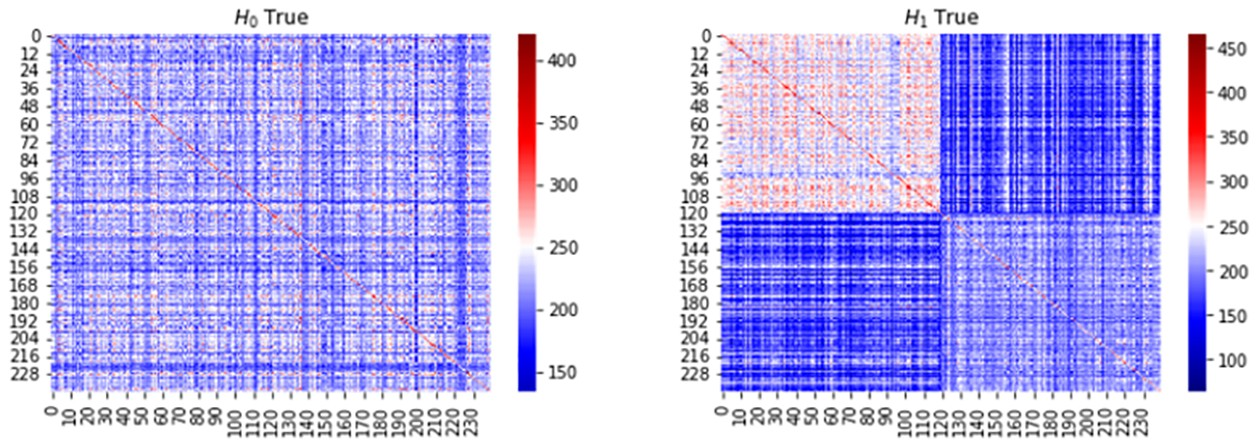
Kernel matrix visualization using heatmaps. The left figure shows a heatmap of the kernel matrix when the null hypothesis is true and the right figure shows a heatmap when the alternative hypothesis is true.

To understand the permutation test it may be beneficial to visualize the kernel matrix, such a visualization can be done as the elements of the kernel matrix **K**_*ij*_ = *k*(*G*_*i*_, *G*_*j*_) measures the similarity between *G*_*i*_ and *G*_*j*_ according to the RKHS of *k*. We calculate the kernel matrix for two cases, one when the null hypothesis is true, and one when the alternative is true. The kernel matrix is then structured as demonstrated in Eq (3.2). The two cases are demonstrated in Fig 4 which displays the heatmaps when when the null hypothesis is true (lef) and when the alternative hypothesis is true (right). When *H*_0_ is true we can see that the kernel matrix appears homogeneous giving us a reason not to reject the null hypothesis. Practically, if we perform a permutation test which is essentially the same as shuffling the rows and columns of kernel matrix and recalculating the MMD, we would see values that are very close to the original sample estimate. Conversely, when *H*_1_ is true then the kernel matrix is heterogeneous and a clear block structure appears within the kernel matrix. This gives us a reason to reject the null hypothesis. Now the permutation of the kernel matrix and recalculation of the MMD would give lower estimates and therefore a low p-value. Furthermore, note that in both cases we can see that the diagonal blocks have higher numerical values than the off-diagonal blocks. The reason being is that objects are more similar to themselves than other objects. This fact can lead to a problem called the diagonal dominance problem if the kernel used to assess the similarity between collections of graphs is too specific in how it assesses similarity in structure. This also motivates why it is important to study and consider the testing framework using various types of graph kernels. We emphasize that although a visualization can be a good aid, it does not replace the *p*-value and should solely be used for empirical diagnostic purposes.

### 3.7 Kernel closure properties

As we will later see, most graph kernels can not take negative weights explicitly into account. However, to incorporate this possible information we will assume that the following closure property of kernels holds for graph kernels [71].

-
$k\left(\left(G_{i},G_{j}\right),\left(G_{i}^{\prime },G_{j}^{\prime }\right)\right)=k_{1}\left(G_{i},G_{i}^{\prime }\right)k_{2}\left(G_{j},G_{j}^{\prime }\right)$ ,
-
$k\left(\left(G_{i},G_{j}\right),\left(G_{i}^{\prime },G_{j}^{\prime }\right)\right)=k_{1}\left(G_{i},G_{i}^{\prime }\right)+k_{2}\left(G_{j},G_{j}^{\prime }\right)$ .

Therefore to allow negative weights in the graph edges we can split each graph *G* into *G*^(−)^ and *G*^(+)^ where *G*^(−)^ is the subgraph containing all nodes but only negative edges and *G*^(+)^ is defined analogously. Then we perform the MMD test on the tensor-product kernel *k*(*G*, *G*′) = *k*((*G*^(+)^, *G*^(−)^), (*G*′^(+)^, *G*′^(−)^) = *k*(*G*^(+)^, *G*′^(+)^)*k*(*G*^(−)^, *G*′^(−)^). We could also perform two separate MMD tests: One for two samples containing only the positive graphs and the other test on two samples containing only the negative graphs and then report the Bonferroni adjusted *p*-value when the joint decision on both sets of graphs is combined into a decision rule. However, the Bonferroni adjustment is conservative and a set of synthetic experiments performed in this manner showed that the tenor-product method results in a higher power for the resulting test.

## 4 Graph kernels

In order to undertake the graph testing framework, one must determine the space upon which the kernel similarity will be measured and what family of kernels will be utilized. There exist many families of similarity measures and kernel mappings between graphs that are based on different transformations of the underlying graph structures. In this context, when dealing with graph kernels we usually have to define the feature vector explicitly. This implication means that computing graph kernels is often time-consuming. For example, at first glance, it might be a good idea to define a kernel between graphs that counts the number of all subgraphs. However, computing the all subgraph kernel is NP-hard as it is essentially the same decision problem as finding hamiltonian paths, [64, 65].

There is an additional problem when dealing with graph kernels. Namely, finding an injective graph kernel that separates different graphs completely. This problem is also known to be NP-hard, see [64]. To elaborate, we need the notion of complete graphs kernels and isomorphic graphs.

**Definition 4.1** (Isomorphic Graphs). *Two graphs G and G*′ *are isomorphic if there is a bijection* *ψ* : *V*(*G*) ↦ *V*(*G*′) *such that* ∀(*u*, *v*) ∈ *E*(*G*) ⇔ (*ψ*(*u*), *ψ*(*v*)) ∈ *E*(*G*′).

We denote that *G* and *G*′ are isomorphic by *G* ≃ *G*′.

**Definition 4.2** (Complete Graph Kernel). *Let* Ω *be a set that consists of graphs and let* ϕ:Ω↦H *be a mapping. Furthermore, let* k:Ω×Ω↦R *be such that* $<\phi \left(G\right),\phi \left(G^{\prime }\right){>}_{H}=k\left(G,G^{\prime }\right)$. *If ϕ is injective then k is called a complete graph kernel*.

Computing any complete graph kernel is at least as hard as deciding whether two graphs are isomorphic. This can be seen as follows. If *ϕ* is injective then $||\phi \left(G\right)-\phi \left(G^{\prime }\right){\left|\right|}_{H}=<\phi \left(G\right)-\phi \left(G^{\prime }\right),\phi \left(G\right)-\phi \left(G^{\prime }\right){>}_{H}=k\left(G,G\right)+k\left(G^{\prime },G^{\prime }\right)-2k\left(G,G^{\prime }\right)=0$ if and only if *G* ≃ *G*′, [64]. Therefore, we have a trade-off between complexity and expressiveness and one concludes from this that it is intractable to compute complete graph kernels when performing graph testing as undertaken in this work. However, as will be illustrated in the remainder of this section, there do exist tractable graph kernels which have affordable complexity and are expressive. Here we will introduce the graph kernels used in this study. For a survey on graph kernels see [57, 58].

Many graph kernels assume node-labeled graphs, but the graphs may not be labelled. To deal with this problem, graphs can be labeled by labeling the nodes according to the node degrees. If the graph is attributed one can also try to bin the attributes to create labels. Graph kernels that assume node-labeled graphs can also be used on edge-labeled graphs. This can be done by labeling each node by concatenating the edge labels from its edges in alphabetical order.

In the remainder of this section we will introduce the following families of graph kernels: Random walk kernels on graphs (with various sub-families as special cases); shortest path kernels on graphs; Weisfeiler-Lehman graph kernels; optimal assignment graph kernels; pyramid matching graph kernels; propagation graph kernels and Wasserstein Weisfeiler-Lehman graph kernels. Each family of kernels will be defined formally and described with regard to the interpretation of what the resulting kernel proximity measures are seeking to evaluate when comparing graph samples from two population samples, as required in this graph testing context.

### 4.1 Convolution kernel

The convolution kernel forms a basis for most graph kernels and is thus worth mentioning. We can think of structured data (for example graphs) as objects that are composed of subobjects or substructures. For example, a string is composed of smaller strings. Using this property the idea is to compute the product of subkernels and sum over the set of allowed decompositions. Formally, the convolution kernel is defined the following way, [57, 59]:

**Definition 4.3** (Convolution Kernel). *Let* $R=R_{1}\times \ldots R_{d}$ *denote the space of components such that a graph G* ∈ Ω *decomposes into elements of* R. *Furthermore, let* R:R×Ω↦{TRUE,FALSE} *denote the mapping from components to objects, such that* $R\left(r_{1},\ldots r_{d},G\right)=R\left(\vec{r},G\right)=TRUE$ *if and only if the components* $\vec{r}\in R$ *make up the graph G* ∈ Ω, *and let* $R^{-1}\left(G\right)=\left\{\vec{r}\in R:R\left(\vec{r},G\right)=TRUE\right\}$. *Then, the R-convolution kernel is*:

$$\begin{array}{c} k_{\text{conv}}\left(G,G^{\prime }\right)=\sum_{r\in R^{-1}\left(G\right)}\;\sum_{r^{\prime }\in R^{-1}\left(G^{\prime }\right)}\;\prod_{i=1}^{d}\;k_{i}\left(r_{i},r_{i}^{\prime }\right), \end{array}$$

*where k*_*i*_ *is a kernel on* $R_{i}$ *for i* ∈ {1, …, *d*}.

In other words, *R* defines a relation on the set $R_{1}\times \ldots \times R_{d}\times \Omega$.

### 4.2 Random walk kernel on graphs

One way to reduce the complexity of graph kernels is to consider walks instead of paths. The idea is similar but the main difference is that walks can visit nodes multiple times while paths can only visit nodes exactly once. Random walk kernel originate from walk kernels. Let $W_{n}\left(G\right)$ be the set of walks with *n* vertices and W(G) be the set of all walks. The walk kernel may then be defined as in Definition 4.4.

**Definition 4.4** (Walk Kernel on Graphs). *Let* $S_{n}$ *denote the set of all possible label sequences of walks of length n, and* $S=\cup_{n\geq 1}S_{n}$. *For any graph G let a weight* λ_*G*_(*w*) *be associated with each walk* $w\in W_{G}$. *Let the feature vector* $\phi \left(G\right)={\left(\phi_{s}\left(G\right)\right)}_{s\in S}$ *be defined by*:

$$\begin{array}{c} \phi_{s}\left(G\right)=\sum_{w\in W(G)}\;\lambda_{G}\left(w\right)1\left(s\;\text{is}\;\text{the}\;\text{label}\;\text{sequence}\;\text{of}\;w\right). \end{array}$$

*Then the walk kernel is defined by*:

$$\begin{array}{c} k_{\text{walk}}\left(G_{1},G_{2}\right)=\sum_{s\in S}\phi_{s}\left(G_{1}\right)\phi_{s}\left(G_{2}\right). \end{array}$$

One can then make a particular selection for the weight function in the kernel to produce what is known as the random walk kernel on a graph. The random walk kernel is obtained with $\lambda_{G}\left(w\right)={\text{Pr}}_{G}\left(w\right)$, where ${\text{Pr}}_{G}\left(w\right)$ is the probability of observing random walk *w*, (usually a Markov random walk). In that case we have [62]:

$$\begin{array}{c} \begin{array}{cc} k_{\text{walk}}\left(G,G^{\prime }\right) & =\sum_{s\in S}\phi_{s}\left(G\right)\phi_{s}\left(G^{\prime }\right)\\ & =\sum_{s\in S}\sum_{w}\text{Pr}\left(w\right)1\left(s\in w\right)\sum_{w^{\prime }}\text{Pr}\left(w^{\prime }\right)1\left(s\in w^{\prime }\right)\\ & =\text{Pr}(\text{label}\left(W_{1}\right)=\text{label}\left(W_{2}\right)). \end{array} \end{array}$$

**Remark**. *In order to define such a kernel on walks, define the normalized adjacency matrix as* $\tilde{A}=AD^{-1}$ *where* **A** *is the adjacency matrix and* **D** *is a diagonal matrix containing the degrees. Note that its rows sum up to 1 and can therefore be thought of as a transition matrix of a Markov chain. Using this construction, consider a Markov process that generates a sequence of vertices* $v_{i_{1}},v_{i_{2}}\ldots$ *according to* $\text{Pr}(i_{k+1}|i_{1},\ldots ,i_{k})={\tilde{A}}_{i_{k+1},i_{k}}$. *We can write* ${\tilde{A}}_{ij}^{t}$ *as the probability of transition from v*_*j*_ *to v*_*i*_ *in t steps. We note however that a valid kernel is still obtained even though no normalization of the adjacency matrix is done, one simply has to make sure that the sum converges, that is, it should be square summable*.

In [64] it was shown that performing a simultaneous random walk on *G* and *G*′ is equivalent to performing a random walk on the direct product graph *G*_×_ which is defined as follows. Given two graphs *G*(*V*, *E*) and *G*′(*V*′, *E*′), their direct product *G*_×_ is a graph with vertex set $V_{\times }=\left\{\left(v_{i},v_{r}^{\prime }\right):v_{i}\in V,v_{r}^{\prime }\in V^{\prime }\right\}$ and edge set $E_{\times }\left\{\left(\left(v_{i},v_{r}^{\prime }\right),\left(v_{j},v_{s}^{\prime }\right):\left(v_{i},v_{j}\right)\in E\wedge \left(v_{r}^{\prime },v_{s}^{\prime }\right)\in E^{\prime }\right)\right\}$.

In [63] they propose a generalized framework for random walk graph kernels. Let **p** and **p**′ denote the initial probability distributions over the vertices of *G* and *G*′, then the corresponding initial probability distribution on the direct product graph is **p**_×_ = **p** ⊗ **p**′. Similarly, if **q** and **q**′ are stopping probabilities then the stopping probability on the direct product graph is **q**_×_ = **q** ⊗ **q**′. The starting and stopping probabilities allow us to put prior information into the kernel design. If *G* and *G*′ are edge-labeled graphs then we can define a weight matrix $W_{\times }\in R^{\left|V|\right|V^{\prime }\left|\times |V|\right|V^{\prime }|}$ with *G*_×_ such that **W**_×_ = Φ(**X**) ⊗ Φ(**X**′) where $X\in X^{\left|V|\times |V\right|}$ and X is a set of labels, including a label for no label. The product is defined as:

$$\begin{array}{c} {\left[\phi \left(X\right)⊗\phi \left(X\right)\right]}_{\left(i-1\right)n^{\prime }+k,\left(j-1\right)n^{\prime }+l}=<\phi \left(X_{ij}\right),\phi \left(X_{kl}^{\prime }\right){>}_{H}. \end{array}$$

As a consequence of this definition, the entries of **W**_×_ are non-zero only if the corresponding edge exists in the direct product graph *G*_×_. We can simply take $\Phi (X)=\tilde{A}$ which gives the weight matrix $W_{\times }=\tilde{A}⊗{\tilde{A}}^{\prime }$. The kernel is finally defined as outlined in Definition 4.5.

**Definition 4.5** (Random Walk Kernel on Graphs). *Let G* = (*V*, *E*) *and G*′ = (*V*′, *E*′) *be two graphs. The random walk kernel is defined as*:

$$\begin{array}{c} k\left(G,G^{\prime }\right)=\sum_{s=0}^{\infty }\;\mu \left(s\right)q_{\times }^{T}W_{\times }^{s}p_{\times }, \end{array}$$ (4.1)

*where* *μ*(*s*) *is a weight function to ensure convergence of the sum and* **p**_×_, **q**_×_, *and* **W**_×_ *are defined as above*.

**Remark**. *A valid kernel is still obtained even if* **p**_×_ *and* **q**_×_ *are not probabilities in* Eq (4.1). *One only has to make sure that the sum converges for all graphs*.

The RW kernel is indeed positive semi-definite as it is possible to write each term in an inner product form, as shown in [63]:

$$\begin{array}{c} q_{\times }^{T}W_{\times }^{s}p_{\times }={\left(q\Phi {\left(X\right)}^{s}p\right)}^{T}\left(q^{\prime }\Phi {\left(X^{\prime }\right)}^{s}p^{\prime }\right). \end{array}$$

Furthermore, as the p.s.d kernels are closed under convex combinations we have that the random walk kernel is a valid p.s.d. kernel.

By taking *μ*(*s*) = λ^*i*^, where $0\leq \lambda <\frac{1}{\lambda_{\times }}$ where λ_×_ is the largest eigenvalue of **W**_×_ we get the so-called geometric random walk kernel $k\left(G,G^{\prime }\right)=q_{\times }^{T}{\left(I-\lambda W_{\times }\right)}^{-1}p_{\times }$. Another choice is *μ*(*s*) = λ^*s*^/*s*! which gives the exponential random walk kernel $k\left(G,G^{\prime }\right)=q_{\times }^{T}\;\text{exp}\left(\lambda W_{\times }\right)p_{\times }$. It is also possible to consider a finite sum in Definition 4.5. Taking only *s* = 0 would only take the prior information of **p**_×_ and **q**_×_ into account, *s* = 1 would perform one transition update, etc. Using a too low *s* does in some sense not take enough moments into account as we will see later in this subsection. The naive way of calculating the geometric random walk kernel involves inverting a |*V*|^2^ × |*V*|^2^ matrix which has a time complexity of *O*(|*V*|^6^). Luckily, using a low-rank approximation of **W** and the Sherman-Woodbury-Morrison lemma [75] it is possible to calculate the kernel in *O*((|*E*| + |*V*|)*r* + *r*^2^) time where *r* is the number of eigenvalues used in the approximation [29]. The fast random walk kernel is given in Definition 4.6.

**Definition 4.6** (r-Approximate Random Walk Kernel, ARKU_plus). *Let G* = (*V*, *E*) *and* *G*′ = (*V*′, *E*′) *be two graphs. The r-approximate random walk kernel is defined as*:

$$\begin{array}{c} k\left(G,G^{\prime }\right)=q_{\times }^{T}{\left(I-c{\hat{W}}_{\times }\right)}^{-1}p_{\times }, \end{array}$$ (4.2)

*where* ${\hat{W}}_{\times }$ *is a low rank approximation of* **W**_×_ *and c is constant to ensure invertibility*.

**Remark**. *There exist well-known methods to make reasonable low-rank approximations of such matrices such as the Nystrom method, see* [76].

**Remark**. *In this study, we will mainly use the r-Approximate Random Walk kernel and it will be the kernel we are referring to when we mention the RW kernel*.

Note that the speed-up scheme can allow for directed, node-labeled, and edge-labeled graphs. It all depends on how one defines the matrix **W**_×_. For example in the case of edge-labels one can define $W_{\times }=\sum_{l=1}^{L}A^{\left(l\right)}⊗A^{\prime (l)}$ where $A_{ij}^{\left(l\right)}=A_{ij}$ if the edge (*v*_*i*_, *v*_*j*_) is labelled as *l* and *L* is the total number of labels [63]. The speed-up scheme for this case can be found in the S1 Appendix. For node labels one can use **W**_×_ = **S**(**A** ⊗ **A**′) where **S** is a diagonal matrix whose (*i*, *i*) entry is 0 if the *i*-th row of (**A** ⊗ **A**′) has label inconsistency, 1 otherwise [29].

Additionally, it is possible to allow node attributes using the random walk kernel [77]. Node attributes encode valuable information such as observations of graph signals. Node signals are, for example, encountered in portfolio design problems where stock returns are connected through a latent graph structure [33] or for insurance pricing of products with geographic risk [78]. These signals can additionally depend on some external features: Let $Z_{i}\in R^{n\times d}$ denote a matrix containing the vertex attributes of a graph *G*_*i*_ and let $\beta_{i}\in R^{d}$ be a (trainable) vector s.t. **y**_*i*_ = ***Z***_*i*_ ***β***_*i*_. Let $y=\text{Vec}\left(\left[y_{i},y_{j}\right]\right)\in R^{n_{i}n_{j}}$ where *n*_*i*_, *n*_*j*_ are the number of nodes in graph *G*_*i*_ and *G*_*j*_. We can then write the resulting kernel as follows [77]:

$$\begin{array}{c} \begin{array}{cc} k(G,G^{\prime }) & =\sum_{s=0}^{\infty }\;\mu \left(s\right)\left(\sum_{i=1}^{|W_{\times }|}\;\sum_{j=1}^{|W_{\times }|}\;y_{i}y_{j}{\left[W_{\times }^{s}\right]}_{ij}\right)\\ & =\sum_{s=0}^{\infty }\;\mu \left(s\right)\left(\sum_{i=1}^{|W_{\times }|}\;\sum_{j=1}^{|W_{\times }|}\;{\left[y\;y^{T}⊙W_{\times }^{s}\right]}_{ij}\right)\\ & =\sum_{s=0}^{\infty }\;\mu \left(s\right)y^{T}W_{\times }^{s}y. \end{array} \end{array}$$ (4.3)

It is possible to perform a kernelized version, let **K**_*y*_ be the kernel matrix containing < *ϕ*(**y**_*i*_), *ϕ*(**y**_*j*_) >. The graph kernel matrix then contains elements given by:

$$\begin{array}{c} \begin{array}{cc} k(G,G^{\prime }) & =\sum_{s=0}^{\infty }\;\mu \left(s\right)\left(\sum_{i=1}^{|W_{\times }|}\;\sum_{j=1}^{|W_{\times }|}\;\left\langle \phi \left(y_{i}\right),\phi \left(y_{j}\right)\right\rangle {\left[W_{\times }^{s}\right]}_{ij}\right)\\ & =\sum_{s=0}^{\infty }\;\mu \left(s\right)\left(\sum_{i=1}^{|W_{\times }|}\;\sum_{j=1}^{|W_{\times }|}\;{\left[K_{y}⊙W_{\times }^{s}\right]}_{ij}\right). \end{array} \end{array}$$ (4.4)

It is possible to interpret the RW in the case of distribution embeddings, namely, the random walk kernel is connected to the average degree. In fact by taking a special case of the weight vector such that *μ*(*s*) = 1 if *s* = 1 and *μ*(*s*) = 0 otherwise, with Φ(*X*) = **A** and **q** = **1** and **p** = **1**/|*V*|, then one may write the mean embedding as follows:

$$\begin{array}{c} \begin{array}{cc} \mu_{P}\left(G^{\prime }\right) & =E_{G∼P}\left[k\left(G^{\prime },G\right)\right]\\ & =E_{G∼P}\left[{\left(1\;A\frac{1}{\left|V\right|}\right)}^{T}\left(q^{\prime }A^{\prime }p^{\prime }\right)\right]\\ & ={\left(q^{\prime }\right)}^{T}A^{\prime }q^{\prime }E\left[K\right], \end{array} \end{array}$$

where *K* is the degree denoted as a random variable. If we take 2 steps and define *μ*(0) = 0, *μ*(1) = 1 and *μ*(1) = 1 and assume that *G* is an undirected graph, then it is possible to show that:

$$\begin{array}{c} E\left[\phi_{G^{\prime }}\left(G\right)\right]={\left(q^{\prime }\right)}^{T}A^{\prime }q^{\prime }\left(E\left[K\right]+E\left[K^{2}\right]\right). \end{array}$$ (4.5)

Unfortunately, we do not get the third moment if we take 3 steps. This can be seen if we inspect *k*^3^ of a 4 node graph, $k_{i}^{3}={\left(A_{i1}+A_{i2}+A_{i3}+A_{i4}\right)}^{3}$, expanding the cubic form has the term *A*_*i*2_*A*_*i*2_*A*_*i*2_ 3 times but it only appears 2 times in the matrix multiplication. However, the third step starts counting triangles among other walks, which means that more steps start comparing more complex structures and essentially allows for comparing higher-order moments. The density of an undirected graph is defined as *ρ* = 2|*E*|/(|*V*|(|*V*| − 1)) = ∑_*i*_ *d*_*i*_/(|*V*|(|*V*| − 1)). Thus, taking **q** = **1**/(**n** − **1**) gives us a mean embedding of the density of the graph as the features of similarity between the graphs being compared.

In summary, we have demonstrated how to construct kernels for measuring similarity between graphs and demonstrated their feasible computational complexity and their explicit interpretations when measuring similarity structures between two graph samples. The focus on the random walk kernel was due to its versatility. It can be used to perform inference on weighted directed or undirected graphs possibly with node labels or edge labels. The Naive time complexity of calculating *K*(*G*, *G*′) is *O*(|*V*|^6^). Luckily, there exist speeding up methods [29] which schemes based on the geometric random walk kernel with time complexity *O*((|*E*| + |*V*|)*r* + *r*^2^) for undirected graphs and *O*(|*V*|^2^*r*^4^ + |*E*|*r* + *r*^6^) for directed graphs, *r* is the number of eigenvalues used in the SVD decomposition of the adjacency matrices. A fast approximation of edge-labeled graphs can be found in the S1 Appendix. In summary, the random walk kernel is a R-convolution kernel that can be used on weighted, directed, undirected, and bipartite graphs with node labels, node attributes, edge labels, and edge attributes. We do not compute the explicit feature mapping although it can sometimes be found, for example, according to definition 4.4.

### 4.3 Shortest-Path kernels on graphs

**Fig 5:**
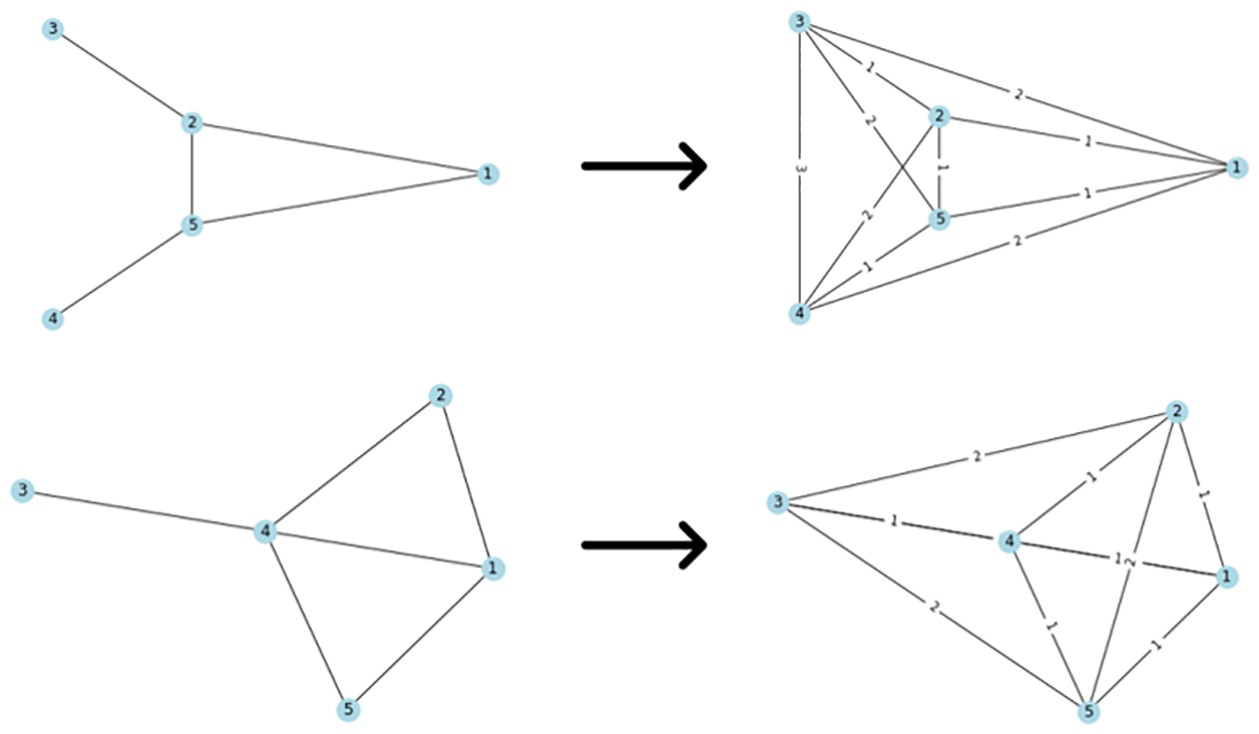
Floyd-transformation of two graphs. The transformation puts an edge between all nodes and labels it by the shortest distance.

As previously observed, computing explicitly path kernels is an NP-hard problem. However, finding the shortest path can be solved in polynomial time, for example, Dijkstra, [79] or Floyd-Warshall, [80]. [65] suggested a kernel based on the shortest paths in a graph as it can be solved in polynomial time. The first step is to transform the original graphs into shortest-path graphs. The resulting graph has an edge between all nodes and the edge is labeled by the shortest distance between the graphs as can be seen in Fig 5. The transformation is called Floyd transformation.

The kernel is then defined in the following manner described in Definition 4.7.

**Definition 4.7** (Shortest-Path Graph Kernel). *Let G*_1_ *and G*_2_ *be two graphs that are Floyd-transformed into S*_1_ *and S*_2_. *The shortest path kernel on S*_1_ = (*V*_1_, *E*_1_) *and S*_1_ = (*V*_1_, *E*_1_) *is defined as*

$$\begin{array}{c} k_{SP}\left(S_{1},S_{2}\right)=\sum_{e_{1}\in E_{1}}\;\sum_{e_{2}\in E_{2}}k_{walk}^{\left(1\right)}\left(e_{1},e_{2}\right), \end{array}$$ (4.6)

*where* $k_{walk}^{\left(1\right)}$ *is a kernel on edge walks of length 1*.

Let *e*_*i*_ = (*v*_*i*_, *u*_*i*_) and *e*_*j*_ = (*v*_*j*_, *u*_*j*_) then $k_{walk}^{\left(1\right)}$ is usually defined as

$$\begin{array}{c} \begin{array}{cc} k_{walk}^{\left(1\right)} & =k_{v}\left(l\left(v_{i}\right),l\left(v_{j}\right)\right)k_{e}\left(l\left(e_{i}\right),l\left(e_{j}\right)\right)k_{v}\left(l\left(u_{i}\right),l\left(u_{j}\right)\right)\\ & +k_{v}\left(l\left(v_{i}\right),l\left(u_{j}\right)\right)k_{e}\left(l\left(e_{i}\right),l\left(e_{j}\right)\right)k_{v}\left(l\left(u_{i}\right),l\left(v_{j}\right)\right), \end{array} \end{array}$$ (4.7)

where *k*_*v*_ is a kernel comparing vertex labels, and *k*_*e*_ is a kernel comparing shortest path lengths. Vertex labels are usually compared via a Dirac kernel, while shortest path lengths may also be compared via a Dirac kernel or another kernel like the Brownian bridge. The time complexity of the shortest path kernel is *O*(|*V*|^4^) [65]. The SP kernel is a R-convolution kernel that can be used on weighted undirected and directed graphs with possible node and/or edge labels. A time complexity of *O*(|*V*|^4^) is very hindering but in the case when the base kernel is a Dirac delta kernel then a significant speed-up scheme can be done. Therefore, in the case of weighted and/or node-attributed graphs, one has to rely on binning strategies in order to compute the kernel efficiently.

### 4.4 The Weisfeiler-Lehman framework

In this section, we will discuss the popular Weisfeiler-Lehman framework for graph kernel construction. The Weisfeiler-Lehman framework (WL) is firstly a label enrichment strategy inspired by the Weisfeiler-Lehman test of graph isomorphism, [61]. The Weisfeiler-Lehman iteration algorithm takes an input graph *G* = *G*_0_ and labelling function *l*_0_ = *l* and returns a sequence {*G*_0_, …*G*_*h*_} = {(*V*, *E*, *l*_0_), …(*V*, *E*, *l*_*h*_)}. Its runtime scales only linearly in the number of edges of the graphs and the length of the Weisfeiler-Lehman graph sequence. Let *k*_0_ be any kernel for graphs that we will call the base kernel. The general Weisfeiler-Lehman kernel with *h* iterations with the base kernel *k*_0_ is defined as

$$\begin{array}{c} k_{WL}^{\left(h\right)}=\sum_{i=0}^{h}\;k_{0}\left(G_{i},G_{i}^{\prime }\right). \end{array}$$ (4.8)

The most commonly used WL kernel is the Weisfeiler-Lehman subtree kernel [60, 61] provided in Definition 4.8.

**Definition 4.8** (Weisfeiler-Lehman Subtree Kernel). *Let G*′ *and G*′ *be two graphs. The [Weisfeiler-Lehman Subtree Kernel is defined as the vector inner product*

$$\begin{array}{c} \begin{array}{cc} k_{\text{WLsubtree}} & =\langle \phi_{WLsubtree}\left(G\right),\phi_{WLsubtree}\left(G^{\prime }\right)\rangle ,\\ \phi_{WLsubtree}\left(G\right) & =(c_{0}\left(G,\sigma_{01}\right),\cdots ,c_{0}\left(G,\sigma_{0|\Sigma_{0}|}\right),\cdots ,c_{h}\left(G,\sigma_{h1}\right),\cdots ,c_{h}\left(G,\sigma_{h|\Sigma_{h}|}\right)), \end{array} \end{array}$$

*where σ*_*ij*_ *is letter number j in the alphabet* Σ_*i*_ *after i number of WL-iterations. That is, we obtain a new alphabet after each WL iteration. c*_*i*_(*G*, *σ*_*ij*_) *is the count of occurrences of the letter σ*_*ij*_ *in the graph G*.

By looking at the definition we can see that the graph features considered by the WL subtree kernel essentially count different rooted subtrees in the graph. For the Weisfeiler-Lehman subtree, we compute the feature mapping explicitly and it has a runtime complexity of *O*(*h*|*E*|) [60]. The WL framework kernel can be used on undirected and directed node-labeled graphs.

### 4.5 Optimal assignment kernels

Optimal assignment kernels act to assign parts of one object to the parts of the other object, for example, matching of vertices of two graphs, such that the similarity is maximized. However, the optimal assignment procedure does not always give rise to a positive semi-definite kernel [81]. [69] consider a particular class of base kernels that give rise to positive semi-definite kernels. Let X be a set of possible components that can be extracted from the graphs in Ω, Π_*n*_ be the set of all possible permutations of [1, …, *n*] and $X\in X^{n}$ and $X^{\prime }\in X^{m}$ be a set of components from the graphs *G* and *G*′, for example, the nodes. Then the optimal assignment kernel is defined as:

$$\begin{array}{c} k_{OA}^{k_{0}}\left(X,X^{\prime }\right)=\underset{\pi \in \Pi_{n}}{\text{max}}\;\sum_{i=1}^{n}k_{0}\left(x_{i},x_{\pi_{i}}^{\prime }\right), \end{array}$$

where *k*_0_ is called the base kernel. If the objects have different cardinality then we may fill the smaller set by new objects *z* with k(z,x)=0,∀x∈X. [69] show that the WL algorithm defines a strong hierarchy on the set of all vertices and thus the resulting optimal assignment called the Weisfeiler-Lehman Optimal Assignment (WLOA) kernel will be a positive semi-definite kernel as detailed in Definition 4.9.

**Definition 4.9** (Weisfeiler-Lehman Optimal Assignment Kernel). *Let G* = (*V*, *E*) *and G*′ = (*V*′, *E*′) *be two graphs. The Weisfeiler-Lehman optimal assignment kernel is defined as*

$$\begin{array}{c} k_{WLOA}\left(G,G^{\prime }\right)=k_{OA}^{k_{0}}\left(V,V^{\prime }\right), \end{array}$$

*where k*_0_ *is the following base kernel*:

$$\begin{array}{c} k_{0}\left(v,v^{\prime }\right)=\sum_{i=0}^{h}\delta \left(l_{i}\left(v\right),l_{i}\left(v^{\prime }\right)\right), \end{array}$$

*where l*_*i*_(*v*) *is the label of node v at the end of the i-th iteration of the Weisfeiler-Lehman relabeling procedure*.

The WLOA kernel can be used on node-labeled graphs and has a computational cost of *O*(*h*|*E*|).

#### 4.5.1 Pyramid match graph kernel

The pyramid match graph kernel [68], based on the pyramid match kernel of [67], first embeds the vertices of each graph into a low-dimensional vector space using the eigenvectors of the largest in magnitude eigenvalues of the adjacency matrix of the graph. The procedure then partitions the feature space into regions of increasingly larger size and a weighted sum of the matches that occur at each level is taken. Two points are matched if they fall into the same region. Given a sequence of levels from 0 to *L*, then at level *l*, the *d*-dimensional unit hypercube has 2^l^ cells along each dimension and *D* = 2^*l*^*d* cells in total. Let $H_{G}^{l}$ and $H_{G^{\prime }}^{l}$ denote the histograms of the graphs *G* and *G*′ at level *l* and $H_{G}^{l}\left(i\right)$, $H_{G^{\prime }}^{l}\left(i\right)$, the number of vertices of *G*, *G*′ that lie in the *i*^*th*^ cell. The pyramid match kernel is then given in Definition 4.10.

**Definition 4.10** (Pyramid Match Graph Kernel). *Let G and G*′ *be two graphs. The pyramid match graph kernel is defined as*:

$$\begin{array}{c} k\left(G,G^{\prime }\right)=I\left(H_{G}^{L},H_{G^{\prime }}^{L}\right)+\sum_{l=0}^{L-1}\;\frac{1}{2^{L-l}}\left(I\left(H_{G}^{l},H_{G^{\prime }}^{l}\right)-I\left(H_{G}^{l+1},H_{G^{\prime }}^{l+1}\right)\right), \end{array}$$ (4.9)

*where*

$$\begin{array}{c} I\left(H_{G}^{l},H_{G^{\prime }}^{l}\right)=\sum_{i=1}^{D}\;\text{min}\;\left(H_{G}^{l}\left(i\right),H_{G^{\prime }}^{l}\left(i\right)\right). \end{array}$$

The kernel can be performed on labeled graphs by matching only vertices that share the same labels. The emerging kernel for labeled graphs corresponds to the sum of the separate kernels:

$$\begin{array}{c} k\left(G,G^{\prime }\right)=\sum_{i=1}^{c}k^{i}\left(G,G^{\prime }\right), \end{array}$$ (4.10)

where *c* is the number of distinct labels and *k*^*i*^(*G*_1_, *G*_2_) is the pyramid match kernel between the sets of vertices of the two graphs which are assigned the label *i*. The pyramid match kernel can be performed on weighted undirected graphs with or without node labels. The time complexity of the pyramid kernel is *O*(*d*|*V*|*L*).

#### 4.5.2 Propagation kernel

Propagation kernels are based on monitoring how information spreads through a set of given graphs, see [66]. We start by considering (partially) labeled graphs without attributes, where *V* = *V*_*L*_∪*V*_*U*_ is the union of labeled and unlabeled nodes. We monitor the distribution of labels encountered by random walks. Let $P_{0}\in R^{\left|V|\times |\Sigma \right|}$ be the prior label distribution of all nodes in *V* where *i*-th row ${\left(P_{0}\right)}_{i}=p_{0,v_{i}}$ corresponds to the prior label distribution of node *v*_*i*_. Note there are *k* number of labels. If node *v*_*i*_ ∈ *V*_*L*_ is observed then $p_{0,v_{i}}=\delta_{l(v_{i})}$. If the nodes are attributed then the “prior” is set as $p_{0,v_{i}}=x_{i}$ where *x*_*i*_ is the attributed vector of node *i*. The label diffusion process is

$$\begin{array}{c} P_{t+1}\leftarrow TP_{t}, \end{array}$$

where **T** = **A** **D**^−1^ is the transition matrix and row *i* of **P**_*t*_ is the distribution of labels at iteration *t* for node *v*_*i*_. If we consider an absorbing node set *S* ⊂ *V*, then the absorbing random walk $\hat{T}$ is defined as.

$$\begin{array}{c} {\hat{T}}_{ij}=\{\begin{array}{cc} 0 & \text{if}\;i\in S\;\text{and}\;i\neq j\\ 1 & \text{if}\;i\in S\;\text{and}\;i=j\\ T_{ij} & \text{otherwise}. \end{array} \end{array}$$

The label propagation process is

$$\begin{array}{c} P_{t+1}\leftarrow \hat{T}P_{t}. \end{array}$$

If *S* = *V*_*L*_ then we have the label propagation algorithm for node classification in [82].

Note that other propagation may be defined. The kernel proceeds by comparing the label/attribute propagation matrix **P**_*t*_ for graph *G* to the label/attribute propagation matrix $P_{t}^{\prime }$ of for graph *G*′ and sums each comparison for *t* = 1, …, *T* where *T* is the maximum number of propagation steps. In order to make the kernel more efficient the propagation kernel uses a hash function that maps the rows of the propagation matrices label distribution or the propagated attributes to integer-valued bins. [66] suggest early stopping rather than the steady-state as the intermediate distributions obtained by the diffusion during the convergence process provide useful insights about the graph structure.

**Definition 4.11** (Propagation Kernel). *Let G and G*′ *be two node-labeled or node-attributed graphs. Define b*_*t*_ *as the number of integer bins (or the number of labels) occupied by the nodes of G and G after applying the hashing function to the node attributes at the t-th iteration of the algorithm. Let also c*_*t*_(*G*, *i*) *be the number of nodes of G placed into bin i at the t-th iteration of the algorithm. Then, the propagation kernel with t*_*max*_ *iterations between Graphs* *G and G*′ *is defined as*:

$$\begin{array}{c} \begin{array}{cc} K_{t_{max}}\left(G,G^{\prime }\right) & =\sum_{t=1}^{t_{max}}K\left(G_{t},G_{t}^{\prime }\right)\\ & =\langle \phi \left(G\right),\phi \left(G^{\prime }\right)\rangle , \end{array} \end{array}$$

*where*

$$\begin{array}{c} \phi \left(G\right)=[c_{0}\left(G,1\right),\ldots ,c_{0}\left(G,b_{0}\right),\ldots ,c_{t_{max}}\left(G,1\right),\ldots ,c_{t_{max}}\left(G,b_{t_{max}}\right)]. \end{array}$$

The kernel is very general as it can be used to construct kernels for many graph types, including node-labeled, partially labeled, unlabeled, directed, and node-attributed graphs. Computing the kernel has a time complexity of *O*(*t*_*max*_|*V*|). The total running time of one pair of graphs is *O*((*t*_*max*_ − 1)|*E*| + *t*_*max*_|*V*|).

#### 4.5.3 Wasserstein Weisfeiler-Lehman graph kernels

The Wasserstein Weisfeiler-Lehman graph kernel (WWL) was developed in [28] and can be applied to attributed, labeled, and weighted graphs. The idea is to calculate a Weisfeiler–Lehman-inspired embedding scheme that works for both categorically labeled and continuously attributed graphs which are then coupled with a graph Wasserstein distance.

Given two sets of matrices $X\in R^{n\times m}$ and $X^{\prime }\in R^{n^{\prime }\times m}$, the Wasserstein distance between them is defined as:

$$\begin{array}{c} W_{1}\left(X,X^{\prime }\right)=\underset{P\in \Gamma (X,X^{\prime })}{\text{min}}\;\left\langle P,M\right\rangle , \end{array}$$

here **M** is the distance matrix between each elements of **X** and **X**′, P∈Γ is a transport/joint probability matrix and 〈⋅, ⋅〉 is the Frobenius dot product. The transport matrix P contains the fractions that indicate how to transport the values from **X** to **X**′ in the most efficient way.

**Definition 4.12** (Wasserstein Weisfeiler-Lehman Graph Kernel). *Consider two labeled graphs G* = (*V*, *E*) *and G*′ = (*V*′, *E*′). *The Wasserstein Weisfeiler-Lehman Graph Kernel is defined as*:

$$\begin{array}{c} k_{WWL}=e^{-\lambda D_{W}}, \end{array}$$

*where* λ > 0 *and* **D**_*W*_ *is the graph Wasserstein distance defined as*:

$$\begin{array}{c} D_{W}^{f}\left(G,G^{\prime }\right)=W_{1}\left(f\left(G\right),f\left(G^{\prime }\right)\right), \end{array}$$

*where* $f:G\mapsto R^{|V|\times m}$ *is an embedding scheme defined as* $f^{h}\left(G\right)=concatenate\left(X_{G}^{0},\ldots ,X_{G}^{h}\right)$ *where* $X_{G}^{h}=[l_{i}\left(v_{1}\right),\ldots ,l_{i}{\left(v_{\left|V|\right)}\right]}^{T}$ *where l*_*i*_(*v*) *is the label of node v after the i-th iteration of the WL relabeling procedure and h is the total number of WL iterations*.

The ground distance matrix **M** is the Hamming distance for node-labeled graphs. This graph kernel can be used on undirected graphs with node labels and can be used on node-attributed graphs with the right embedding scheme as well. The node feature vector is not computed explicitly. The Wasserstein distance is the computational bottleneck with a complexity of *O*(|*V*|^3^ log |*V*|) naively but there exist speedup tricks [28].

### 4.6 Enhancing graph kernels

We should briefly mention that new kernels can be constructed by manipulating and combining kernels as explained in [71]. These include:

$$\begin{array}{c} \begin{array}{c} k(G,G^{\prime })=k_{1}\left(G,G^{\prime }\right)+k_{2}\left(G,G^{\prime }\right),\\ k(G,G^{\prime })=k_{1}\left(G,G^{\prime }\right)k_{2}\left(G,G^{\prime }\right),\\ k(G,G^{\prime })=k_{3}\left(\phi \left(G\right),\phi \left(G^{\prime }\right)\right), \end{array} \end{array}$$ (4.11)

where *k*_1_ and *k*_2_ are some graph kernels, and *k*_3_ is kernel over $R^{d}\times R^{d}$ where *d* is the dimension of the feature vector *ϕ*(*G*). These properties can then be used to show that the following kernel manipulations are further possible:

$$\begin{array}{c} \begin{array}{c} k(G,G^{\prime })=p(k_{3}\left(\phi \left(G\right),\phi \left(G^{\prime }\right)\right)),\\ k(G,G^{\prime })=\text{exp}(k_{3}\left(\phi \left(G\right),\phi \left(G^{\prime }\right)\right)),\\ k(G,G^{\prime })=\text{exp}(||\phi \left(G\right),\phi \left(G^{\prime }\right)|{|}^{2}/\left(2\sigma^{2}\right)), \end{array} \end{array}$$ (4.12)

where *p* is a polynomial, || ⋅ || is some norm and *σ*^2^ > 0 is some constant. We saw in the previous subsections that many graph kernels explicitly compute feature vectors, meaning that they essentially transform the graph data to vector data. The final step is often to apply a linear kernel to the constructed vector data [69]. However, it is well-known that better results are often obtained when a non-linear kernel such as the polynomial or RBF kernels are used. Therefore, it may be beneficial to apply one of the kernel construction methods in Eq (4.12) to enhance the predictive performance, and indeed, this was suggested in [69]. As noted the third equation in (4.12) shows that any norm can be used. Over the past years, a few developments of p-adic number theory have been made that enhance the understanding of complex networks, see for instance [83]. The p-adic numbers give an extension of the ordinary arithmetic of rational numbers. The p-adic numbers, where *p* is any prime number, come from an alternate way of defining the distance between two rational numbers. The p-adic field $Q_{p}$ is a complete metric space like the real number field, but unlike the reals the p-adics are an ultrametric space, leading to a number of fascinating but often counterintuitive results. A p-adic number is a series of the form:

$$\begin{array}{c} x=x_{-k}p^{-k}+x_{-k+1}p^{-k+1}+\cdots +x_{0}+x_{1}p+\cdots , \end{array}$$ (4.13)

with *x*_−*k*_ ≠ 0, the *x*_*j*_ coefficients are the p-adic digits, i.e. numbers in the set {0, 1, …, *p* − 1}. The natural norm of p-adic numbers is defined as ||*x*||_*p*_ = *p*^*k*^, and the ultrametric property refers to the fact that ||*x* − *y*||_*p*_ ≤ max{ ||*x* − *z*||_*p*_, ||*z* − *y*||_*p*_}. The use of p-adic numbers has recently been suggested in the machine learning literature. For example in [84, 85], who develop so-called p-adic cellular neural networks where the weights of a neural network are p-adic numbers, and [86] who investigate the correspondence between neural networks and p-adic statistical field theories. In the case of kernel learning one may construct a p-adic kernel by combining the p-adic norm and the kernel algebra above as follows:

$$\begin{array}{c} k\left(G,G^{\prime }\right)=\text{exp}\;(\sum_{i=1}^{d}{||\left[\phi \left(G\right)\right]}_{i}-{\left[\phi \left(G^{\prime }\right)\right]}_{i}{\left|\right|}_{p}^{2}/\left(2\sigma_{i}^{2}\right)), \end{array}$$ (4.14)

where $\phi \left(\cdot \right)\in R^{d}$, [*ϕ*(*G*)]_*i*_ is the *i*-th element of *ϕ*(*G*), and $\sigma_{i}^{2}>0$ are some constants for each *i*.

## 5 Synthetic experiments exploring the influence of the graph kernel on the power of two sample graph testing

We performed multiple synthetic studies regarding graph two-sample hypothesis testing where we assumed various different graph distributions. We considered graph distributions that mimic real-world graphs such as binomial graphs, scale-free graphs, and stochastic block matrices with and without node attributes, edge labels, node labels, and outlier graphs [87]. We used the Area Under the Curve (AUC) metric to assert the performance of the kernels. Here we will report the main findings but we refer to the S1 Appendix for the complete study.

### 5.1 Positively and negatively weighted graphs with attributes

We include the experiment when the graphs can have both negative and positive edge weights and node attributes. This experiment is designed to mimic comparison between portfolios, where the nodes are average returns and the graph itself is the precision matrix (minus the diagonal).

The samples were generated in the following manner: First, generate a sample with binomial graphs with degree *k*_*i*_. Next, for each edge present, give an edge weight according to an exponential distribution with scale parameter λ_*i*_. The sign of each edge weight is flipped with probability *p*_*i*_ (note the graph is symmetric so we flip the sign of both edges *e*_*ij*_ and *e*_*ji*_). Finally, for each node generate a normal random variable *X*_*i*_ with mean *m*_*i*_ and standard deviation *s*_*i*_ as its attribute.

**Fig 6:**
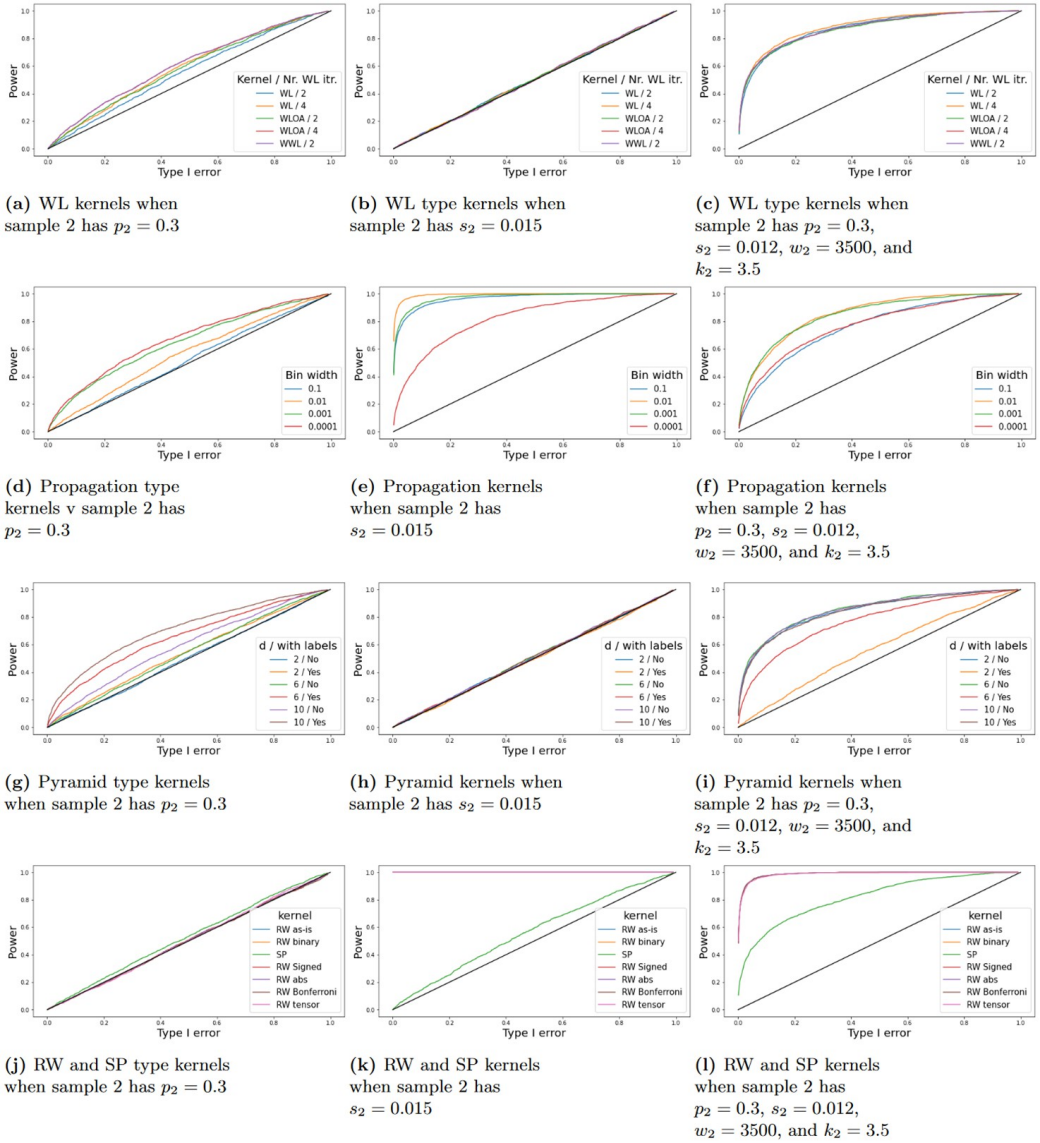
ROC curves. The power of various kernels in the graph two-sample testing problem.

In the experiment we will construct 20 binomial graphs with 20 nodes in each sample and the attributes are set to the following values: *m*_1_ = 0.00038, *s*_1_ = 0.01, *k*_1_ = 4, λ_1_ = 3000, *p*_1_ = 0.35. In the experiment, we changed one parameter at a time for sample 2 and the results can be seen in Fig 6. The MMD_*u*_ estimate of the MMD was used. We both tried using a tensor product kernel $K\left(G_{1},G_{2}\right)=K\left(G_{1}^{\left(+\right)},G_{2}^{\left(+\right)}\right)K\left(G_{1}^{\left(-\right)},G_{2}^{\left(-\right)}\right)$ as explained in section 3.7 and a kernel assuming absolute values for the edge weights for WL, WWL, WLOA, and propagation kernels to incorporate negative weights. We found that the results only differ when the only difference between the graphs was the edge weight sign distributions, therefore we only report the result from the tensor product kernels.

Fig 6 illustrate the results of the experiments performed. In each row, we can see the performance in different scenarios for a fixed basket of kernels. The plots demonstrate the Receiver-Operator (ROC) curves that show Type I error versus the Power of the test. We show this for a variety of experimental settings and kernel choices.

- The WL, WLOA, and WWL kernels utilize the Weisfeiler Lehman iteration scheme on node labels. In our experiment the nodes were labeled according to their degrees. It can be seen that the kernels give good performances when the average degree between the two samples is different and when the probabilities of a negative edge are different. The first case is in concordance with other experiments (the label distributions are different). The latter can be explained as follows: Take the pair $\left(G_{i}^{\left(+\right)},G_{i}^{\left(-\right)}\right)$ and $\left(G_{j}^{\left(+\right)},G_{j}^{\left(-\right)}\right)$, where *i* comes from sample 1 and *j* comes from sample 2. If the two samples have the same probability of negative signs and the same average degree then we would not expect the test to be rejected, however, if the two samples have different sign probabilities but the same average degree, then depending on the magnitude of *p*_*i*_ (the probability of a positive edge), $G_{i}^{\left(-\right)}$ will be more sparse or dense than $G_{j}^{\left(-\right)}$ and the WL iterations will be different, even though the label distribution is the same. Not surprisingly we see that the test has 0 AUC when only the attributes are different. Overall we do not see AUC differences when a different number of WL iterations are used nor from the WWL discount parameter.
- The number of walks *t*_*max*_ in the propagation kernel was held constant in this experiment at 6. The propagation kernel gives non-zero AUC in all cases. We can see that the lower the bin width the higher the power, which makes sense due to the relatively low value of the attributes ±2 * 0.01. However, the lower the bin width the more diagonally dominant the kernel matrix becomes. It might be surprising that the AUC is not zero when *p*_1_ = 0.35 and *p*_2_ = 0.3 but all other attributes remain the same, as the propagation kernel simply normalizes the adjacency matrix and essentially calculates the average node attribute of the neighborhood of each node repeatedly. However, as the probability of a negative sign is different between the two samples we have the fact that $G_{i}^{\left(-\right)}$ will be more sparse or dense than $G_{j}^{\left(-\right)}$. This means that if the bin width of the propagation is smaller than the standard deviation of the kernel then the test will detect differences between the two samples.
- The depth *L* of the pyramid match kernel was held constant in this experiment at *L* = 6. The pyramid kernel has a non-zero AUC when the sign count is different between the two samples. This is not surprising as the sparseness is different between $G_{i}^{\left(-\right)}$ and $G_{i}^{\left(-\right)}$ if *j* and *i* belong to different samples, the same is true for the positive graphs. Interestingly we see that including labels does give better performances even though the average degree between the two samples is the same. We can also see that including more eigenvectors is preferable. Not surprisingly we have a zero AUC when only the attributes are different between the two samples as the pyramid match does not take attributes into account. Lastly, we see that when the two samples are even more different the AUC increases.
- We tested multiple versions of the RW kernel and for the SP kernel, we used a discretization strategy where the attributes were divided by the maximum attribute and then rounded to the first non-zero digit and the weights followed a similar discretization strategy but were rounded to the second non-zero digit. The different versions of the RW and SP all give good AUC except for the case when only the sign probability was different. Interestingly, when attributes are ignored the RW kernel will give good performances when the sign probabilities between the two samples are different. All types of random walk kernels seem to give very good performances while the SP kernel is similar to the propagation kernel. We remark that the reason that the binary RW kernel is doing a good job is that the attributes are different. If only the edge weights are different then the binary RW kernel will give a zero AUC performance.
- We also tried a different labeling scheme for the WL-type kernels. The labels were set by binning the attributes. The attributes were divided by the largest attribute observed in each graph and then the new attribute was rounded using 1 or 2 digits. The test had positive AUC and rounding of 1 or 2 does not matter in this case. However, using a round of 3 gave 0 AUC.

We also tested the MONK estimator of the MMD in the presence of outliers both when the topological structure was observed as an outlier and when the node attributes contained outliers as in Fig 3. We found empirically that when the two samples are generated from the same distribution but one sample contained 5% outliers the monk estimator had significantly lower type I error. We also performed MMD tests on other graph typologies which gave similar results, although we only tested it for binary weights. The main difference is that the shortest path kernel did not manage to discriminate two populations of scale-free graphs with different scaling parameters. The reason is that the shortest path for scale-free graphs is independent of the scaling parameter [88].

The second main synthetic study we undertook which is of direct relevance to understanding when using these methods for the ESG portfolio analysis undertaken in the real data case studies in this manuscript is shown below and pertains to the regularisation of the graph sample estimation.

### 5.2 Effects of sparse graph sample construction via lasso regularization and its influence on graph testing

**Fig 7:**
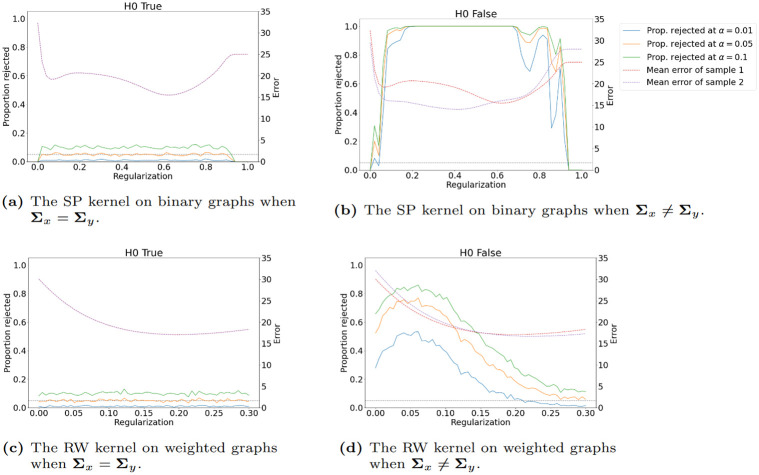
The effect of regularization in graph learning for the kere two-sample problem.

Another experiment vital to the application undertaken in this manuscript for use of this two-sample graph testing inference in portfolio diversification comparisons is the effect of regularization in the graph construction/estimation in this two-sample testing context. Consider two samples ${\left\{x_{i}\right\}}_{i=1}^{n}$ and ${\left\{y_{i}\right\}}_{i=1}^{n}$ such that **x**_*i*_ ∼ N(**0**, **Σ**_*x*_) and **y**_*i*_ ∼ N(**0**, **Σ**_*y*_) with $\Sigma_{x},\Sigma_{y}\in R^{d\times d}$ unknown. It is known that random fluctuations in a sample can lead to spurious correlations in the empirical covariance matrix. The graphical lasso, see section 6.2 has been proposed for filtering out spurious correlations, either by regularizing the covariance [39] matrix or the precision matrix [89]. Let **Θ** = **Σ**^−1^ be the precision matrix. In this example we generate 100 samples from $\text{N}(0,\Theta_{x}^{-1})$ and 100 samples from $\text{N}(0,\Theta_{y}^{-1})$ and estimate **Θ**_*x*_ and **Θ**_*y*_ using graphical lasso. This was performed 50 times, giving two samples of graphs ${\left\{\Theta_{i,x}\right\}}_{i=1}^{5}0$ and ${\left\{\Theta_{i,y}\right\}}_{i=1}^{5}0$. We calculate the *p*-value of the MMD test statistic. The experiment is performed 1,000 times for different regularization parameters both when **Θ**_*x*_ = **Θ**_*y*_ and **Θ**_*x*_ ≠ **Θ**_*y*_. Two cases are considered: 1) We ignore the weights and signs and only consider binary graphs: *A*_*ij*_ = 1 if |Σ_*ij*_| > 0, 0 otherwise. For this case we use the SP kernel 2) We consider the precision matrix as-is and use the r-approximate RW kernel. From Fig 7 we can see a very interesting result. Namely in the case when *H*_1_ is true, then the power of the test is low with no regularization while it increases when *ρ* is near its optimal value. This clearly shows the significance of using sparsity-inducing estimation methods like the glasso. We also see when *H*_0_ is true that the proportion rejected stays within the type I error *α*. These observations are both true for the binary graph experiment and the weighted graph experiment but note that the power starts to fall down before the error reaches the minimum value for the weighted graph experiment.

## 6 Real data experimental design: Assessing diversification in ESG screened equity portfolios

In this case study, the focus is on the exploration of the screening of assets for inclusion/exclusion in a wealth management portfolio such as those found in the emerging lucrative green finance investing universe of ETFs, Mutual Funds, and 401k superannuation managed accounts constructed by investment managers and self-managed super investors. This market is increasingly making up the bulk of equity investments and as such, it is important to understand better the role that ESG ratings may have in effecting portfolio diversification if screening by ESG ratings is practiced. We demonstrate the methodology on assets within the S&P500 index from the years 2016–2022. The historical prices and ESG scores of each asset were obtained using Yahoo’s publicly available APIs, which is intended for research and educational purposes. The data was used for modelling and demonstration purposes only, and the data collection and analysis methods complied with the terms and conditions of the Yahoo developer API terms of use (The Yahoo terms can be found at Yahoo terms). ESG scores were obtained from ESG scores where the ticker has to be changed for each asset. The historical prices were obtained from prices where the ticker has to be set for each asset.

There are four stages of the experimental design and case study undertaken. Namely, smoothing of the ESG scores, graph estimation, portfolio construction, and portfolio valuation. We start by describing the steps taken to perform the study.

**Stage 1**: **Smoothly Stochastically Interpolating Equity ESG Scores**

The first step is to smooth the ESG scores using a Gaussian Process which is a vital part of the study to give a more accurate screening procedure. Since ESG scores for each of the companies in listed stock exchanges are infrequently produced (scored), the screening procedure adopted will benefit from an interpolation of the time series of ESG scores per company. Furthermore, since ESG scores are typically noisy and may contain outliers, it is beneficial to first smoothly interpolate the scores so that any portfolio screening rules will not be affected adversely by rapid changes in the inclusion/exclusion of assets on a monthly screening and rebalance schedule of a portfolio, simply due to noisy scoring methods.

Hence, in stage 1 of the process, the framework proposed for this case study was a procedure that interpolates and essentially generates more observations which in turn gives more rejection decision data points from the two-sample tests. This in turn allows for a better comparison between the classical portfolio performance metrics and the inter-asset differences measured by the MMD.

**Stage 2**: **Dynamic ESG Screening of Assets into Various ESG Ranked Asset Universes for Portfolio Inclusion**

The obtained smoothed ESG scores were then used as screening criteria to construct two dynamic portfolios. Let *P*_1,*t*_ and *P*_2,*t*_ be two portfolios containing assets *i* ∈ *I* at rebalancing step *t*, where *I* is the set of assets considered for inclusion in the portfolio managers investment thesis/theme. In our study, we considered multiple investment sets, both assets belonging to one particular sector within the SnP500 index and all assets in the SnP500 index. An important note is that the asset set was constructed such that |*I*| mod 3 = 0. This was obtained by randomly removing assets from the original asset set during the entire experiment such that the remaining set was divisible by 3. This was done so that the number of assets in each portfolio was equal. Denote ESG_*i*,*t*_ as the ESG scored observed for asset *i* at time *t*.

At each rebalancing time, *t* the assets were ordered according to their current ESG score and split into three groups. The first group contained assets with the best ESG scores (lowest), the second group contained assets with medium ESG scores and the last group contained assets with poor ESG scores. The portfolios were then updated such that *P*_1,*t*_ contained non-zero weightings of positions only for the best ESG assets from group 1 and *P*_2,*t*_ contained assets with the poorest ESG ratings from group 3 assets (the medium ESG assets were not used at the particular time *t*). We can define the sets more rigorously as follows:

$$\begin{array}{c} \begin{array}{c} P_{1,t}=\sum_{j=1}^{I}\;w_{j}X_{j}I[j\in \{i:i\in I,{\text{ESG}}_{i,t}<Q_{t,1/3\%}\}],\\ P_{2,t}=\sum_{j=1}^{I}\;w_{j}X_{j}I[j\in \{i:i\in I,{\text{ESG}}_{i,t}>Q_{t,2/3\%}\}], \end{array} \end{array}$$

where *I* is the asset set and *Q*_*t*,*j*%_ is the *j*-th quantile of the smoothed ESG scores at time *t*. To emphasize, by construction we have:

$$\begin{array}{c} \sum_{j=1}^{I}\;I[j\in \{i:i\in I,{\text{ESG}}_{i,t}<Q_{t,1/3\%}\}]=\sum_{j=1}^{I}\;I[j\in \{i:i\in I,{\text{ESG}}_{i,t}>Q_{t,2/3\%}\}]=\left|I\right|/3 \end{array}$$

**Stage 3**: **Construction of Two Sets of Graph Samples via Screened Assets by ESG Rankings from Stage 2 Portfolios**

With the screened asset sets, the historical log-returns $R_{1,t},R_{2,t}\in R^{n\times p}$ were extracted for the screened assets in portfolio 1 and portfolio 2. Where *n* is the number of days considered and *p* = |*I*|/3 is the number of assets in each group. A graph representation of inter-asset relations was then estimated for each asset set using the graphical lasso [89].

The graphical lasso estimates the precision matrices **Θ**^(*j*,*t*)^ *j* ∈ {1, 2} using the historical log returns as input (or their covariance). Finally, the estimated precision matrices were used to estimate the adjacency matrices of the two portfolio graphs *G*_1,*t*_ and *G*_2,*t*_ at time *t*. We assumed that vertices cannot be connected to themselves so the diagonal of ${\hat{\Theta }}^{\left(j,t\right)}\;j\in \left\{1,2\right\}$ were ignored/set to zero. The edge weights were defined to be the elements of -${\hat{\Theta }}^{\left(j,t\right)}\;j\in \left\{1,2\right\}$. This is justified by the fact that the negative precision encodes the partial covariance. Additionally to the graph estimation, we used 3 portfolio construction techniques for the assets in *P*_1,*t*_ and *P*_2,*t*_ and analyzed their performances using various portfolio performance metrics. The graph estimation step was performed in a rolling window fashion using every other time point, i.e.e the rebalancing was performed on the set *t* ∈ {*t*_2*i*_ : *i* ∈ {*w*, *w* + 1, …, ⌊*n*/2⌋}} where *w* < *n* is the graph estimation window size.

Furthermore, for each of these screened portfolios, the optimal portfolio weights for *P*_1,*t*_ and *P*_2,*t*_ were determined according to the mean-variance criterion to produce various optimal portfolio strategies: Global Minimum Variance and Maximum Sharpe Ratio.

**Stage 4**: **Two-Sample Graph Testing Inference to Assess Influence of ESG Screening on Portfolio Diversification**

The final step involved using the MMD testing procedure in a rolling window fashion on the two graph samples ${\left\{G_{i,t}\right\}}_{t=1}^{M}\;i\in \left\{1,2\right\}$ where *M* is the total number of graphs in each sample. That is at each time step *t* a kernel two-sample test was performed using the two graph sub-samples ${\left\{G_{i,t}\right\}}_{t=t^{\prime }-s}^{t^{\prime }}\;i\in \left\{1,2\right\}$ where *s* is the size of the graph testing rolling window (not the same as graph estimation rolling window *w*). In our experiment, we used *s* = 20. The overall graph estimation and graph testing procedure are written in a pseudo-code fashion in the S1 Appendix.

### 6.1 Smoothly stochastically interpolating equity ESG scores

**Fig 8:**
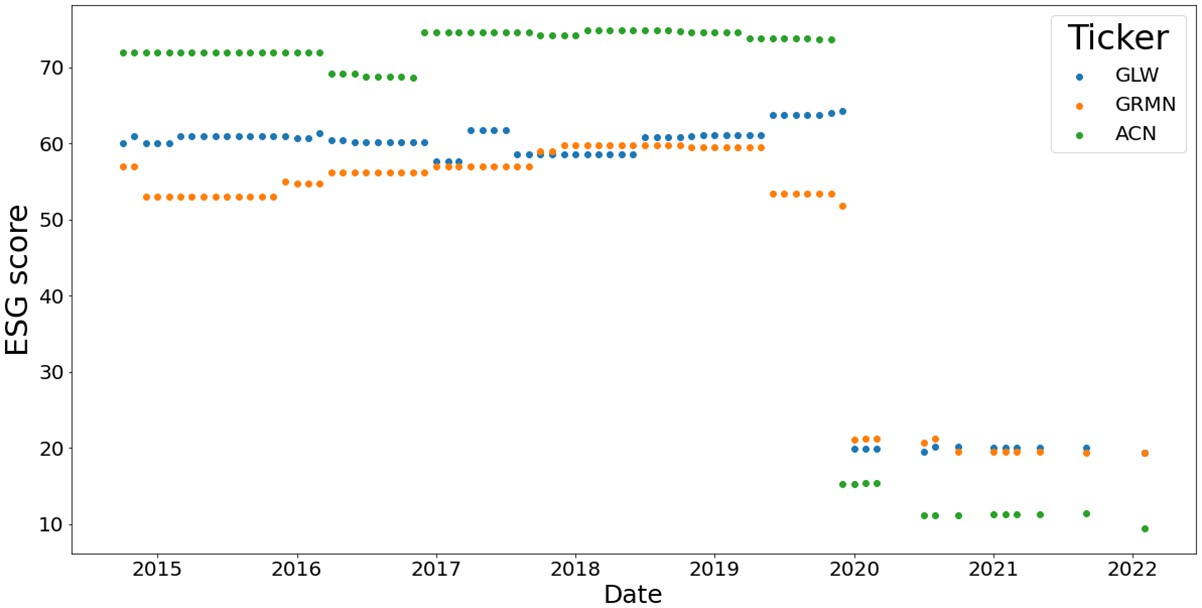
ESG scores for 3 tickers within the SnP500 asset universe.

The ESG data from 2016–2022 was obtained from yahoo finance and shows monthly ESG scores of companies within the SnP500 index. Not all companies within the SnP500 have ESG scores and some only have very few observations, around 10–20. Companies with 20 or fewer observations are discarded giving 429 companies to analyze. Fig 8 shows the ESG time series of 3 companies. First, we can see that the ESG scores are piecewise constant functions, Secondly, ESG scores are usually observed monthly but during COVID and after the observation get less frequent. Finally, there is a level shift around 2020. The shift does not occur in the same month for all series as we can see in the figure, BA and GILD shift before ADI.

We remove the shift by taking the mean before and after the shift and subtract the difference from the original series $y_{i}^{\left(orig\right)}$ for company *i* before the shift *k*:

$$\begin{array}{c} \begin{array}{cc} y_{i,1:k} & =y_{i,1:k}^{\left(orig\right)}-\frac{1}{k}\;\sum_{t=1}^{k}\;y_{i,t}^{\left(orig\right)}+\frac{1}{T-k}\;\sum_{t=k+1}^{T}\;y_{i,t}^{\left(orig\right)},\\ y_{i,k+1:T} & =y_{i,k+1:T}^{\left(orig\right)}, \end{array} \end{array}$$

where *T* is the number of observations. We are interested in studying the effect of ESG scores on portfolios in a rolling window manner. However, due to the characteristics of the ESG series, it will pose some difficulties as we only have a maximum of 75 observations for an individual series compared to 2681 observations for financial log-returns. To generate daily estimates we fit a Gaussian process (GP) smoother to each ESG index [90]. Formally, GPs are defined as:

**Definition 6.1** (Gaussian Process). *Denote* f(x):X↦R *a stochastic process parametrised with state-space* x∈X, *where* $X\subset R^{d}$. *The random function* *f*(*x*) *is a Gaussian process if all its finite-dimensional distributions are Gaussian, where for any* n∈N, *the random vector* [*f*(**x**_1_), *f*(**x**_2_), …, *f*(**x**_*n*_)] *is jointly normally distributed*.

We can therefore interpret a GP as equivalently characterized by the following class of random functions:

$$\begin{array}{c} f≔\{f\left(\cdot \right):X\mapsto R:\;f\left(\cdot \right)∼GP\left(\mu \left(\cdot ;\theta_{\mu }\right),k\left(\cdot ,\cdot ;\theta_{k}\right)\right\}, \end{array}$$

with $\mu (\cdot ;\theta_{\mu }):X\mapsto R$ and $k\left(\cdot ,\cdot ;\theta_{\mu }\right):X\times X\mapsto R^{+}$ such that:

$$\begin{array}{c} \mu \left(\cdot ;\theta_{\mu }\right)≔E\left[f\left(\cdot \right)\right],\;k\left(\cdot ,\cdot ;\theta_{k}\right)≔E\left[\left(f\left(\cdot \right)-\mu \left(\cdot ;\theta_{\mu }\right)\right)\left(f\left(\cdot \right)-\mu \left(\cdot ;\theta_{\mu }\right)\right)\right]. \end{array}$$

This means that, as Gaussian processes are simply a Gaussian distribution, we can completely specify them using the mean function *μ* and a covariance function *k*. Assume that we observe noisy observations **y** (the ESG score):

$$\begin{array}{c} y=f(x)+\epsilon , \end{array}$$

where $\epsilon ∼N(0,\sigma^{2}I)$. Let the subscript _*_ denote unobserved values then we can write the joint distribution of **y** and f_*_ as:

$$\begin{array}{c} {}[\begin{array}{c} y\\ f_{*} \end{array}]∼N([\begin{array}{c} \mu (X)\\ \mu (X_{*}) \end{array}],\;[\begin{array}{cc} k\left(X,X\right)+\sigma^{2}I & k(X,X_{*})\\ k(X_{*},X) & k(X_{*},X_{*}) \end{array}]), \end{array}$$

where *k*(**X**, **X**) is a matrix obtained by applying *k* pairwise to each data point in **X** and *μ*(**X**) is the mean vector obtained by applying the mean function row-wise to **X**. Deriving the conditional distribution of f_*_ we arrive at the key predictive equations for Gaussian process regression

$$\begin{array}{c} \begin{array}{cc} E[f_{*}] & =\mu \left(X_{*}\right)+k\left(X_{*},X\right){\left[K\left(X,X\right)+\sigma^{2}I\right]}^{-1}\left(y-\mu \left(X\right)\right),\\ \text{cov}(f_{*}) & =k\left(X_{*},X_{*}\right)-k\left(X_{*},X\right){\left[K\left(X,X\right)+\sigma^{2}I\right]}^{-1}k\left(X,X_{*}\right). \end{array} \end{array}$$

**Fig 9:**
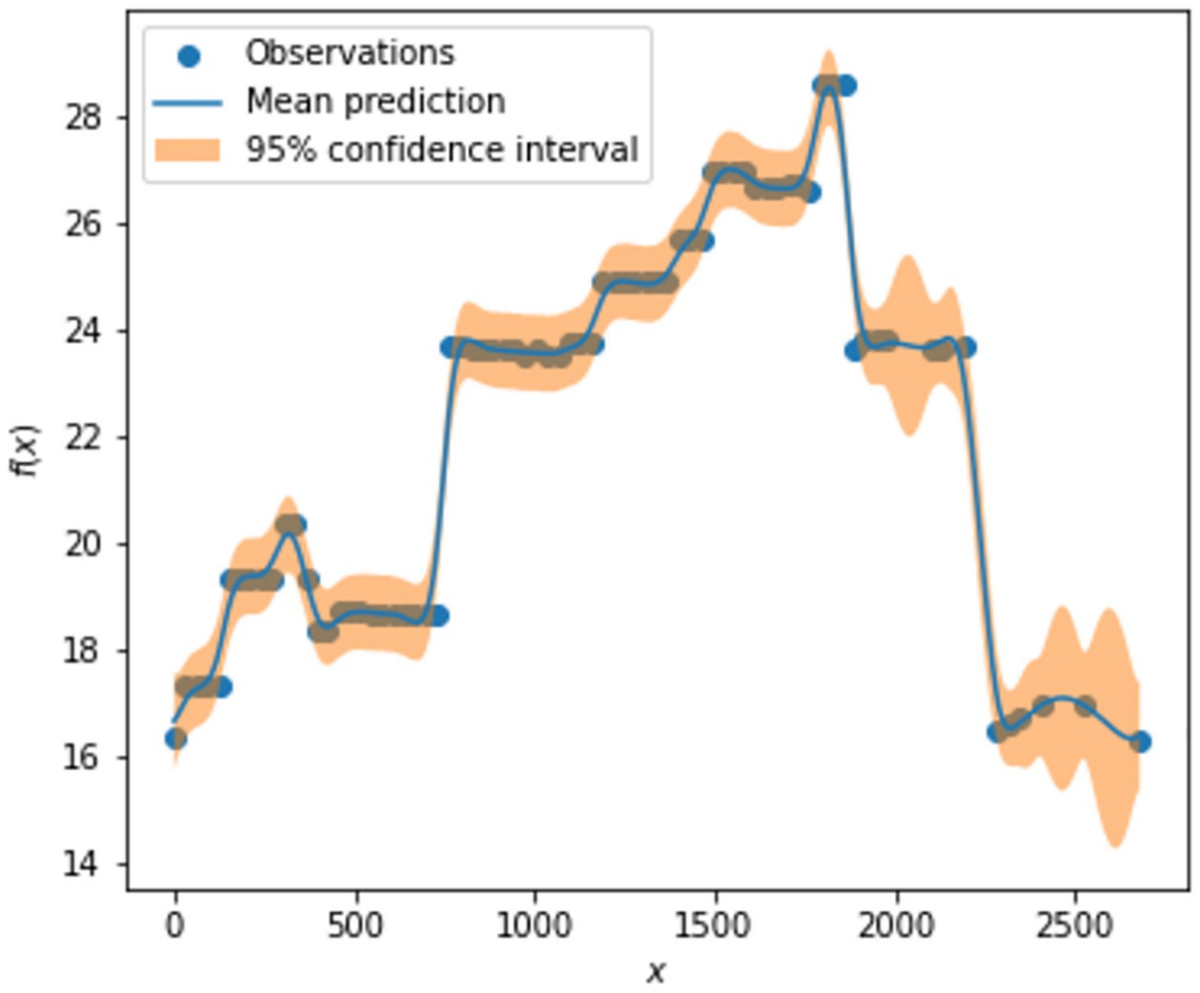
GP smoothed ESG of the AAPL ticker.

There are usually three sets of parameters that need to be estimated when fitting a Gaussian Process *θ*_*μ*_, the hyperparameters of the mean function, *θ*_*k*_, the parameters of the covariance function and *σ*^2^ the noise level of the observations. In our case, we assume that the mean function is zero leaving only *θ*_*k*_ and *σ*^2^ to be estimated. In this manuscript, we are more interested in smoothing rather than forecasting so we use a stratified cross-validation to choose the best *σ*^2^ while *θ*_*k*_ is estimated by maximizing the log-likelihood for a given value of *σ*^2^. The metric used in each fold is ${\hat{R}}^{2}$ defined as:

$$\begin{array}{c} {\hat{R}}^{2}=1-\frac{\sum_{i\in \text{test}}{\left(y_{i}-{\hat{y}}_{i}\right)}^{2}}{\sum_{i\in \text{test}}{\left(y_{i}-{\bar{y}}_{i}\right)}^{2}}, \end{array}$$

where ${\hat{y}}_{i}$ are the predictions within the test set of the GP and ${\bar{y}}_{i}$ is the mean of the sample. We used the Sklearn [91] library to fit the gaussian processes. The overall smoothing algorithm is explained in the S1 Appendix. We use a Matern kernel with *ν* = 3/2 to smooth the ESG scores (using the time index as a feature). It gave the maximum log-likelihood compared to other kernels tested such as other Matérn kernels, the RBF kernel, and the rational quadratic kernel. Fig 9 shows the GP smoothed ESG scores of an asset.

### 6.2 Construction of two sets of graph samples via screened assets by ESG rankings from stage 2 portfolios

In this section, we explain how one can take the multivariate time series of returns for the screened assets for Portfolio 1 and Portfolio 2 and construct a time series of regularised graph samples that will form the input to the graph testing framework. Furthermore, since the graphs should capture aspects of the structure between the time series of asset returns in each ESG score-screened sub-population, it is important to base the graph estimation on a measure of portfolio diversification. The most natural of these is the correlation structure between the assets that will be included in each portfolio.

Such samples of graphs constructed from financial returns should contain the most important connections within the ensemble of assets being investigated. These connections are, however, by no means obvious, and unraveling them all is a very complicated task. Here we will apply the most widely used graph estimation method to estimate the latent financial sample graphs which are then to be used in the portfolio optimization procedure and graph testing framework.

The graphical lasso [39, 89] has been proposed for filtering out spurious correlations. It can either be performed as a regularization on the covariance matrix or the precision matrix but the objective function is non-convex for the covariance matrix making the precision matrix problem a bit easier to work with. The estimated graph will be sparse because of the imposed l1 regularization.

**Fig 10:**
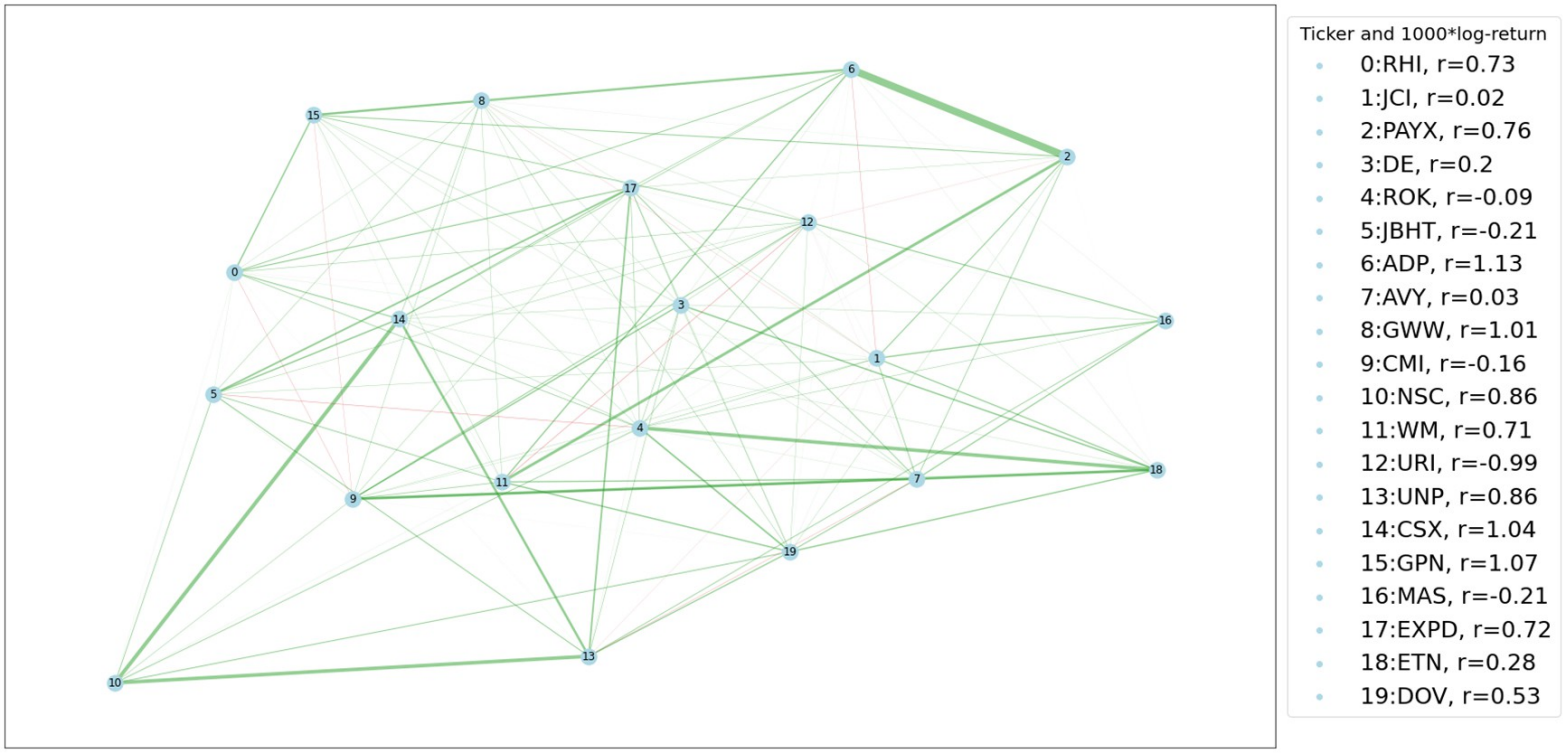
Example of a learnt graph. Example of a top ESG Portfolio Graph for the Industrial sector at a time *t*. Each vertex represents an asset, and each vertex has furthermore the mean return over the graph construction period as an attribute. The mean return has been multiplied by 1000.

At time *t* we estimate two graphs/precision matrices, one that encodes financial covariances between assets with good ESG scores (those screened to be included in portfolio 1) and one that encodes financial covariances between assets with poor ESG scores (those screened to be included in portfolio 2). Further, assume that we use the past *n* observations to do so and let **Θ**^(1,*t*)^ = **Σ**^−1,(1,*t*)^, **Θ**^(2,*t*)^ = **Σ**^−1,(2,*t*)^ be the precision matrix at time *t* for the good ESG portfolio and the poor ESG, respectively. The graphical lasso is:

$$\begin{array}{c} \begin{array}{cc} L(\Theta^{\left(k,t\right)}) & =\frac{n}{2}\text{log}\;\text{det}\left(\Theta^{\left(t\right)}\right)-\frac{1}{2}\sum_{i=t-n}^{n}{\left(r_{i}-\mu \right)}^{T}\Theta^{\left(k,t\right)}\left(r_{i}-\mu \right)-\frac{nd}{2}\;\text{log}\;\left(2\pi \right)-\sum_{i=1}^{d}\sum_{j=1}^{i-1}\rho_{ij}\left|\Theta_{ij}^{\left(k,t\right)}\right|, \end{array} \end{array}$$

where *d* is the number of features, ***μ*** is the mean of the log-returns, and ***r***_*i*_ are realisations of the log-returns. The first 3 terms are the normal log-likelihood function and the last term is the lasso penalty term. For simplicity we often use the same penalty for each edge, that is, *ρ* = *ρ*_*ij*_. The objective is concave and can thus be solved by using a convex optimization procedure such as a coordinate-wise descent algorithm [89, 92, 93]. Fig 10 shows an example of a graph obtained from estimated precision matrix.

**Remark**. *The edge weights and signs are ignored for the WWL, WL, and WLOA kernels. The SP and Propagation kernel use absolute values of edge weights as those kernels can not use negative weights. The reason why we choose the absolute value instead of a tensor product is that very few negative edge weights were observed after the graph estimation was performed. The SP kernel further bins the node attributes and edge weights to speed up the kernel calculation*.

We tested two graph estimation packages: The Huge package [31] in R and Sklearn package in python [91]. The two packages differ slightly as the Huge package penalizes the diagonal of **Θ** whilst the Sklearn package does not. We chose to use the Huge package as it was easier to use for our purposes.

The (extended) BIC [94] approach is to maximize the posterior probability of a model *M*_*i*_ given the data **y**:

$$\begin{array}{c} P\left(M_{i}|y\right)=\int p\left(y|\theta_{i}\right)p\left(\theta_{i}\right)d\theta_{i}=\int \;\text{exp}\;\left(\text{log}\;\left(p\left(y|\theta_{i}\right)p\left(\theta_{i}\right)\right)\right)d\theta_{i}. \end{array}$$

We use Laplace’s method to approximate the integral, this only works if *p*(*y*|*θ*_*i*_)*p*(*θ*_*i*_) has a global maximum and decays rapidly to zero away from the maximum. We expand log (*p*(*y*|*θ*_*i*_)*p*(*θ*_*i*_)) about the mode ${\hat{\theta }}_{i}$ obtaining:

$$\begin{array}{c} Q=\text{log}\;\left(p\left(y|\theta_{i}\right)p\left(\theta_{i}\right)\right)\approx \text{log}\;\left(p\left(y|{\hat{\theta }}_{i}\right)p\left({\hat{\theta }}_{i}\right)\right)+{\left(\theta_{i}-{\hat{\theta }}_{i}\right)}^{T}{\nabla Q|}_{{\hat{\theta }}_{i}}+\frac{1}{2}{\left(\theta_{i}-{\hat{\theta }}_{i}\right)}^{T}H_{{\hat{\theta }}_{i}}\left(\theta_{i}-{\hat{\theta }}_{i}\right), \end{array}$$

where $H_{{\hat{\theta }}_{i}}=\nabla \nabla^{T}{Q|}_{{\hat{\theta }}_{i}}$ is the Hessian. Let ${\tilde{H}}_{{\hat{\theta }}_{i}}=-H_{{\hat{\theta }}_{i}}$, since *Q* obtains the maximum at ${\hat{\theta }}_{i}$ we have:

$$\begin{array}{c} \begin{array}{cc} P(M_{i}|y) & \approx p\left(y|{\hat{\theta }}_{i}\right)p\left({\hat{\theta }}_{i}\right)\int \;\text{log}\;(-\frac{1}{2}{\left(\theta_{i}-{\hat{\theta }}_{i}\right)}^{T}\;{\tilde{H}}_{{\hat{\theta }}_{i}}\left(\theta_{i}-{\hat{\theta }}_{i}\right))d\theta \\ & =p\left(y|{\hat{\theta }}_{i}\right)p\left({\hat{\theta }}_{i}\right)\frac{{\left(2\pi \right)}^{m/2}}{|{\tilde{H}}_{{\hat{\theta }}_{i}}{|}^{1/2}}, \end{array} \end{array}$$

where *m* is the number of parameters or number of edges in our case. For a large number of samples *n* we have:

$$\begin{array}{c} {\tilde{H}}_{sk}=-\frac{\partial^{2}\;\text{log}\;\left(\prod_{j=1}^{n}p\left(y_{j}|{\hat{\theta }}_{i}\right)\right)}{\partial \theta_{s}\partial \theta_{k}}\underset{n\to \infty }{\to }-\frac{\partial^{2}E\left[\sum_{j}n\;\text{log}\;p\left(y_{j}|{\hat{\theta }}_{i}\right)\right]}{\partial \theta_{s}\partial \theta_{k}}=-nF_{sk}, \end{array}$$

where **F** is the Fisher information. This asymptotic relation and taking the log further gives us the following equation:

$$\begin{array}{c} \begin{array}{c} \text{log}\;P\left(y|M_{i}\right)=\text{log}\;\left(p\left(y|{\hat{\theta }}_{i}\right)\right)+\text{log}\;\left(p\left({\hat{\theta }}_{i}\right)\right)+0.5m\;\text{log}\left(2\pi \right)-0.5m\;\text{log}\;n-0.5\;\text{log}\;F. \end{array} \end{array}$$

If we assume an uninformative flat prior then $\text{log}\;(p\left({\hat{\theta }}_{i}\right))=0$ and furthermore drop the Fisher matrix and the *m* log 2*π* term then the above formula reduces to the BIC, where we have multiplied the above with 2:

$$\begin{array}{c} \begin{array}{c} \text{BIC}=2\;\text{log}\;(p\left(y|{\hat{\theta }}_{i}\right))-m\;\text{log}\;n. \end{array} \end{array}$$

Now if we are in a high-dimensional setting, such as graph/covariance estimation where *m* >> *n* we have the BIC often prefers large models and will then possibly choose spurious covariates, to see this, say that there are 1000 covariates. The set of one covariate has a cardinality 1000 while the set of two covariate models has a cardinality of 1000*999/2, meaning that the two covariate model is 999/2 times more likely to be selected. This means that we should not set an uninformative flat prior in a high-dimensional setting. A prior that has been suggested is p(θ^i)∝(d(d+1)/2|E|)-β [94] with 0 < *β* < 1, where *d* is the dimension of each **r**_*i*_, resulting in the so-called extended BIC or EBIC:

$$\begin{array}{c} \begin{array}{c} \text{EBIC}=2\;\text{log}\;\left(p\left(y|{\hat{\theta }}_{i}\right)\right)-m\;\text{log}\;n-4\beta \left|E\right|\text{log}\;\left(d\right). \end{array} \end{array}$$

We can see that the higher the *β*, the sparser the optimal model is according to EBIC.

### 6.3 Monotone transformation

The graphical lasso relies heavily on the assumption of normality. Unfortunately, this assumption is usually discredited even by the log transformation of the returns. We consider a transformation suggested by [95] called the nonparanormal to transform the log-returns via smooth functions, meaning that we transform ***r*** by using *f*(***r***) = (*f*_1_(*r*_1_), …, *f*_*d*_(*r*_*d*_)) where *f*(***r***) is multivariate Gaussian. The random vector ***r*** is said to be nonparanormal if there exists functions ${\left\{f_{j}\right\}}_{j=1}^{d}$ such that *f*(***r***) ∼ *N*(***μ***, **Σ**) and we write:

$$\begin{array}{c} r∼\text{NPN}(\mu ,\Sigma ,f). \end{array}$$

It can be seen that the NPN is simply a Gaussian copula when *f*_*i*_ are monotone and differentiable and that the joint probability density of ***r*** is given by:

$$\begin{array}{c} p\left(r\right)=\frac{1}{{\left(2\pi \right)}^{d/2}{\left|\Sigma \right|}^{1/2}}\;\text{exp}\;(-\frac{1}{2}\left(f\left(r\right)-\mu \right)\Sigma^{-1}\left(f\left(r\right)-\mu \right))\prod_{j=1}^{d}\left|f_{j}^{\prime }\left(r_{j}\right)\right|. \end{array}$$

The density is not identifiable so it is assumed that *f*_*j*_ preserve means and variances:

$$\begin{array}{c} \mu_{j}=E[f\left(r_{j}\right]=E\left[r_{j}\right]\;\;\text{and}\;\;\sigma_{j}^{2}=\Sigma_{jj}=\text{V}\left[f\left(r_{j}\right)\right]=\text{V}\left[r_{j}\right]. \end{array}$$

This all implies that

$$\begin{array}{c} f_{j}\left(r_{j}\right)=\mu_{j}+\sigma_{j}\Phi^{-1}\left(F_{j}\left(x\right)\right), \end{array}$$

where *F*_*j*_ is the marginal distribution of *r*_*j*_ and Φ is the cumulative distribution function of the standard normal. We use the Winsorized empirical distribution, function to estimate *F*_*j*_ as suggested by [95]:

$$\begin{array}{c} {\tilde{F}}_{j}\left(r_{j}\right)=\{\begin{array}{cc} \delta_{n} & \text{if}\;{\hat{F}}_{j}\left(r_{j}\right)<\delta_{n}\\ {\hat{F}}_{j}\left(r_{j}\right) & \text{if}\;\delta_{n}\leq {\hat{F}}_{j}\left(r_{j}\right)\leq 1-\delta_{n}\\ 1-\delta_{n} & \text{if}\;{\hat{F}}_{j}\left(r_{j}\right)>1-\delta_{n}, \end{array} \end{array}$$

where $\hat{F}$ is the empirical distribution and $\delta_{n}=\frac{1}{4n^{1/4}\sqrt{\pi \;\text{log}\;n}}$.

**Remark**. *In addition to the nonparanormal transform, we also tried using standard scaling. Scaling is important if the features are of different scales which is generally not the case for log returns. We compared the results when the log returns were given as-is, scaled, and transformed nonparanormally. There were some discrepancies between the estimated graphs, as expected and in the end, we chose the nonparanormal transform*.

### 6.4 Portfolio construction and metrics

To maintain comparability between the graphs and the portfolios we used the estimated graph/precision matrix, as explained in subsection 6.2 to get an estimate of the covariance matrix ${\hat{\Sigma }}^{\left(j,t\right)}j\in \left\{1,2\right\}$ by setting:

$$\begin{array}{c} {\hat{\Sigma }}^{\left(j,t\right)}={\hat{\Theta }}^{-1,(j,t)}. \end{array}$$

This is justified as the graphical lasso can be seen as a robust estimate of **Θ**^−1,(*j*,*t*)^. To assess portfolio performance under ESG screening we consider three portfolio construction methodologies. The Passive Equal-Weighted Portfolio(PEW) is a buy-and-hold portfolio that serves a long-term investment strategy with minimal interaction with the market. The weights of all assets are set to be equal *w*_*i*_ = 1/*d* for asset *i* with *d* being the number of assets. PEW prohibits short positions. Global Minimum Variance Portfolio (GMV) portfolio seeks to find the portfolio with the lowest volatility [96, 97]. The optimal weights are allocated by an optimization problem subject to minimizing the variance of the return of the portfolio. The GMV’s optimization is given by:

$$\begin{array}{c} \underset{w}{\text{min}}\;\frac{1}{2}w^{T}\;\Sigma w\;\;\text{s.t.}\;\;\sum_{i}w_{i}=1. \end{array}$$

The analytic solution is $w=\frac{\Theta 1}{1^{T}\Theta 1}$ where **1** is a vector of ones. The Maximum Sharpe Ratio Portfolio (MS) is a portfolio that serves as a benchmark to achieve the highest return per unit risk. The weights are allocated by the optimization problem subject to maximizing the Sharpe ratio of the portfolio:

$$\begin{array}{c} \underset{w}{\text{max}}\;w^{T}\mu -\frac{1}{2}w^{T}\;\Sigma w\;\;\text{s.t.}\;\;\sum_{i}w_{i}=1. \end{array}$$

The analytic solution is $w=\frac{\Theta \mu }{1^{T}\Theta \mu }$.

Note that the optimization objectives allow for long and short positions for the GMV and the MS portfolios. This can lead to extreme asset allocations that result in an excessive concentration of positions in a few assets. The Federal Reserve usually limits the short-selling positions within a portfolio to be 50% of the portfolio weight, i.e., 150–50 fund investment strategy. In practice, the short-selling ratio ranges from 120–20 to 150–50, with 130–30 being the most common [98]. Consequently, we limit the short position to no more than 30% of all positions. We approximate the box constraints by renormalizing the asset weights rather than adding them to the optimization constraints in the following manner:

$$\begin{array}{c} w_{i}=\{\begin{array}{cc} 0.3\frac{w_{i}}{\sum_{j\in S}w_{j}} & \;\sum_{j\in S}w_{j}<-0.3\\ w_{i} & \;\sum_{j\in S}w_{j}\geq -0.3 \end{array},\;\forall i\in S, \end{array}$$

where *S* ⊆ {1, …, *d*} are the indices of the assets being shorted. Similarly, for the long positions

$$\begin{array}{c} w_{i}=\{\begin{array}{cc} 1.3\frac{w_{i}}{\sum_{j\in L}w_{j}} & \;\sum_{j\in S}w_{j}>1.3\\ w_{j} & \;\sum_{j\in L}w_{j}\leq 1.3 \end{array},\;\forall i\in L, \end{array}$$

where *L* ⊆ {1, …, *d*} are the indices of the assets being longed.

We can visualize the geometry of the different portfolios available for the top ESG universe and the low ESG universe by looking at the efficient frontier at different times *t*. The efficient frontier can be found by minimizing the following object for different portfolio returns ${\bar{r}}_{p}$,

$$\begin{array}{c} \underset{w}{\text{min}}\;\frac{{\bar{r}}_{p}^{T}w}{\sqrt{w^{T}\;\Sigma \;w}}, \end{array}$$ (6.1)

which is similar to the MS objective. The efficient frontier can be found by setting some predefined values for ${\bar{r}}_{p}$ and plotting the outcome using the following equation for the standard deviation:

$$\begin{array}{c} {\bar{\sigma }}_{p}=\frac{a{\bar{r}}_{p}^{2}-2b{\bar{r}}_{p}+c}{ac-b^{2}}, \end{array}$$ (6.2)

where *a* = **1**^*T*^ **Θ** **1**, *b* = **1 Θ** ***μ*** and *c* = ***μ***^*T*^ **Θ** ***μ***.

We consider multiple portfolio metrics to assess the performance of various ESG-screened portfolios. The diversification ratio measure is defined as:

$$\begin{array}{c} \text{DRatio}=\frac{w^{T}\sigma }{\sqrt{w^{T}\;\Sigma \;w}}, \end{array}$$

where ***σ***^2^ = diag(**Σ**) is variance of the individual assets. We also look at the Value-at-risk (VaR) diversification measure:

$$\begin{array}{c} \text{DVaR}=\frac{{\text{VaR}}_{q}\left(R_{p}\right)}{\sum_{i=1}^{N_{p}}\;w_{i}\;{\text{VaR}}_{q}\left(R_{i}\right)}, \end{array}$$

where *q* is the quantile, *R*_*p*_ and *R*_*i*_ are random variables for the return of the portfolio and asset *i*, respectively. We estimate VaR by using the empirical quantile. We additionally consider return metrics such as the Sharpe, Treynor, and Sortino values and the omega function evaluated at zero which is a measure that allows for higher moment information in the returns distribution. We exclude the exact formulas here but defer them to the technical S1 Appendix.

**Remark**. *Each of these metrics along with the average log returns were calculated over the same period as was used to estimate the graphical lasso. That is at time t we use the historical data with index i* ∈ [*t* − *n*, *t*].

## 7 ESG ratings and diversification benefits

Typically portfolio assessment is concentrated on the net portfolio risk and return profiles, we take a novel approach to study and quantize differences in inter-asset relations and graph structures which in turn affect diversification differences between two portfolios selected according to a dynamic screening criterion ranking assets for inclusion/exclusion according to their ESG ranking as outlined in the previous section, so-called good and poor ESG portfolios. The main objective is to analyze the rejection rates which occur when one tests differences in inter-asset relations in two portfolios. We further want to test if the rejection rates of graph kernels are translated in differences in diversification and risk-adjusted returns profiles and whether the rejections can be explained by differences in the ESG profiles.

To answer these questions we will look at the sensitivity and conservatism of different graph kernels. The kernel should not be too conservative but at the same time not too sensitive meaning that the kernel should not fail to reject all of the time but also not reject all of the time. After identifying the right kernels we then count how often the good ESG portfolio has better diversification and risk-adjusted return metrics while also reporting the rejection decision. A visual example is then demonstrated to showcase which differences the kernel is quantifying. We further analyze whether a MMD test rejection is directly translated to portfolio performances. This would mean that inter-asset relations affect portfolio risk and return profiles. To further assess the relationship between portfolio metrics and MMD rejections rates we will perform PCA and classification analysis to try and identify which portfolio metrics mostly affect a rejection.

**Remark**. *Portfolio testing does not have to be a cross-sectional study of two portfolios but also a temporal study where the inter-asset relationships change can be tested before and after some events, such as interest rate hikes, ESG scoring changes, or legalization changes*.

Different combinations of assets in the SnP 500 were used to construct 2 portfolios in a rolling-type fashion for different scenarios, as explained in section 6. The 2 dynamic portfolios will represent good ESG companies and low ESG companies respectively in multiple scenarios: When portfolios are only allowed to invest in single sectors and when the portfolios are allowed to select all assets in the SnP500 index. Table 1 lists how many assets belonged to each portfolio, i.e. |*I*|/3, for different asset worlds (sectors and global). It can be seen that some portfolios only contained a few assets (Basic Materials, Energy, and Communication) while others had more (Global, Consumer cyclical, Industrials, Healthcare and Financial).

**Table 1:** Number of assets in each portfolio, the good and poor ESG, for different asset worlds (sectors and global).

Global	Util.	Mat.	Energy	Ind.	Def.	Cyclical.	Com.	Fin.	Health.	Estate.	Tech.
143	9	5	6	20	10	18	5	20	18	9	19

**Fig 11:**
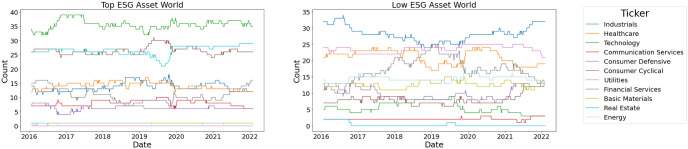
Asset ESG group membership. The figure shows how many assets belong to each sector at different time points for the two global portfolios (top and bottom).

**Fig 12:**
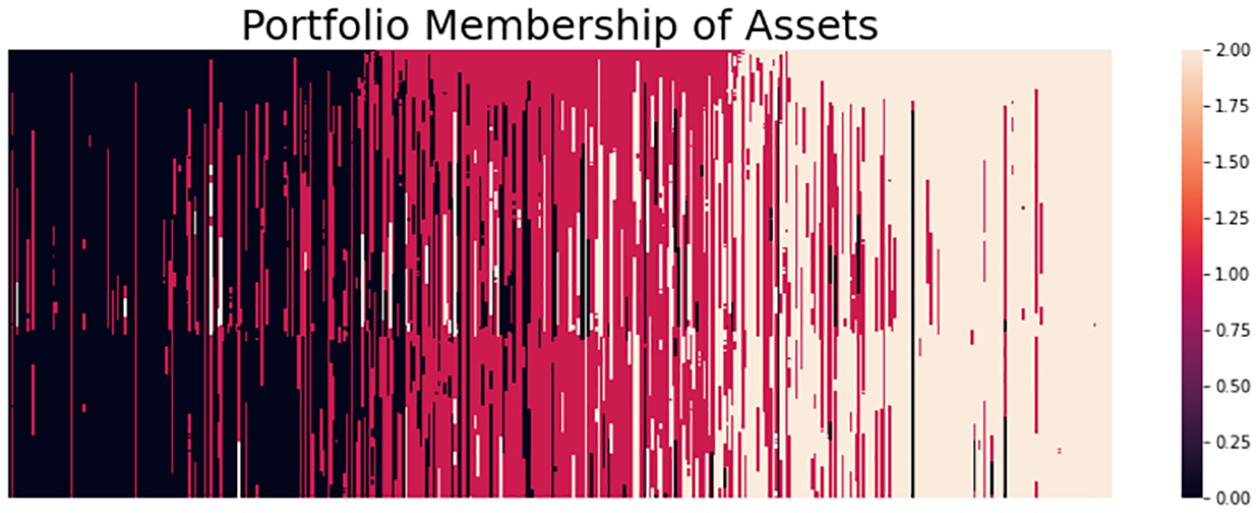
Changes in portfolio membership for the global assets. Each column is an asset and the row is time. Each cell indicates which portfolio the asset (column) belonged to at each rebalance date (row). Time flows from the top to the bottom. Color intensity 0 indicates that the asset belonged to the top ESG portfolio, 1 the medium ESG portfolio (not used in this study), and 2 the low ESG portfolio.

We add a few more comments on the portfolio structure. Fig 11 counts the number of sectors contained in the global good ESG portfolio and poor ESG portfolio. The top ESG portfolio mainly consists of assets that belong to a sector with better overall ESG scores such as the Healthcare, Real Estate, Technology, and Financial sectors while the poor ESG portfolio has assets that belong to poor ESG sectors such as the Materials, Utilities, Energy, and Industrials. We can also see in Fig 12 that the membership of assets is fairly consistent, this feature was also observed for portfolios in other sectors. That is the assets in portfolios *P*_1,*t*_ and *P*_2,*t*_ are mostly stable with only a few assets jumping between portfolios at each rebalancing step.

Table 2 gives an overview of the relation between the E score and CO_2_ emission. It can be seen that the Utilities, Energy, Materials, and Industrials sectors have by far the most CO_2_ emissions. We keep this table in mind and study if there are similarities in inter-asset relations of those sectors.

**Table 2:** Average environmental scores and amount of carbon emission of some companies in the S&P 500 separated by Global Industry Classification Standard (GICS) sector ranked by direct CO_2_ emissions, where ${\text{n}}_{{\text{CO}}_{2}}$ is the number of the assets available for calculating the number of carbon emissions, and ${\text{N}}_{{\text{CO}}_{2}}$ is the number of whole assets in the sector. The table was taken from [98] with permission.

Rank	Sector	E score	CO_2_ Emissions (Ton)	CO_2_ Emissions (%)	${\text{n}}_{{\text{CO}}_{2}}$	${\text{N}}_{{\text{CO}}_{2}}$
1	Utilities	14.90	29,884,383.57	46.06	21	28
2	Energy	15.64	16,488,511.67	25.41	15	21
3	Materials	13.08	6,951,694.12	10.71	24	28
4	Industrials	7.38	3,572,569.61	5.51	45	74
5	Communication Services	1.19	2,566,298.00	3.96	13	24
6	Consumer Staples	7.42	2,539,331.17	3.91	24	30
7	Consumer Discretionary	4.00	1,384,162.29	2.13	41	63
8	Information Technology	3.45	618,047.76	0.95	48	73
9	Real Estate	3.55	390,973.88	0.60	24	29
10	Health Care	1.49	337,598.75	0.52	39	64
11	Financials	1.56	147,601.10	0.23	38	64

Note: We cannot obtain the amount of carbon emissions from all companies in S&P 500. The coverage ratio can be calculated by ${\text{n}}_{{\text{CO}}_{2}}/{\text{N}}_{{\text{CO}}_{2}}$

In Table 3 we report the rejection rate, the number of rejections divided by the total number of tests performed, of different graph kernels using *α* = 0.01. It can be seen that the pyramid kernel and the SP kernel with binned attributes are too sensitive as they reject almost all of the time. The rejection rates do depend on the asset universe, for example, the RW kernel rejects more often for the Materials, Energy, Consumer Cyclical, and Real Estate sectors. The results for each asset universe are mostly heterogeneous across different kernels. Furthermore, it can be seen that the MONK estimator is usually more conservative than the MMD_*u*_ and MMD_*l*_ estimators, as expected.

**Table 3:** The number of rejections for different graph kernels assuming a cut-off of *α* = 0.01. The RW kernel discount was set to *c* ≈ 10^−9^ (varied a little between tests). The propagation used *w* = 0.0001 and *t*_*max*_ = 6. The number of WL iterations was set to *h* = 2. The WWL used a discount of λ = 0.1. The pyramid kernel used *L* = 10 and *d* = 6. Note that other hyperparameters were tested as well, giving very similar results. The SP kernel used a discretization of the continuous edge weights and node attributed, using rounding of 3 digits and 1 digit respectively. The MONK estimator used *Q* = 5 partitions.

	MMD\Kernel	RW attr.	RW	Prop.	SP	SP attr.	WL	WLOA	WWL	Pyr. labels	Pyramid
Global	MONK	0.66	0.7	0.86	0.87	0.82	0.92	0.87	0.82	1	1
	MMD_*u*_	0.7	0.8	0.9	0.93	0.98	0.96	1	1	1	1
	MMD_*l*_	0.67	0.75	0.9	0.95	0.98	0.96	0.99	0.99	1	1
Materials	MONK	0.95	0.98	0.91	0.9	0.98	0.49	0.5	0.52	1	1
	MMD_*u*_	0.97	0.99	0.97	0.96	1	0.52	0.52	0.54	1	1
	MMD_*l*_	0.98	0.99	0.99	0.98	1	0.55	0.55	0.55	1	1
Utilities	MONK	0.97	0.95	0.39	0.93	0.97	0.76	0.76	0.77	0.96	0.95
	MMD_*u*_	0.98	0.96	0.5	0.98	1	0.88	0.92	0.92	1	1
	MMD_*l*_	0.96	0.95	0.61	0.99	1	0.91	0.93	0.92	1	1
Industrials	MONK	0.79	0.68	0.6	0.6	0.91	0.57	0.47	0.52	0.97	1
	MMD_*u*_	0.83	0.78	0.68	0.68	0.99	0.8	0.91	0.9	1	1
	MMD_*l*_	0.78	0.63	0.7	0.65	1	0.88	0.92	0.93	1	1
Energy	MONK	0.72	0.86	0.67	0.9	0.99	0.65	0.64	0.64	1	1
	MMD_*u*_	0.77	0.88	0.73	0.99	0.99	0.71	0.73	0.74	1	1
	MMD_*l*_	0.8	0.92	0.85	1	1	0.76	0.79	0.79	1	1
Communication	MONK	0.89	0.79	0.74	0.91	0.98	0.52	0.5	0.54	1	1
	MMD_*u*_	0.95	0.86	0.85	0.97	1	0.57	0.58	0.59	1	1
	MMD_*l*_	0.92	0.86	0.96	0.99	1	0.7	0.71	0.71	1	1
Cyclical	MONK	0.98	0.92	0.76	0.88	0.98	0.54	0.53	0.55	0.99	1
	MMD_*u*_	0.99	0.96	0.82	0.91	1	0.82	0.94	0.94	1	1
	MMD_*l*_	0.98	0.95	0.84	0.92	1	0.86	0.9	0.9	1	1
Defensive	MONK	0.9	0.68	0.6	0.72	0.97	0.79	0.74	0.73	0.99	0.99
	MMD_*u*_	0.94	0.79	0.69	0.84	1	0.95	0.95	0.94	1	1
	MMD_*l*_	0.92	0.63	0.76	0.92	1	0.98	0.97	0.97	1	1
Real Estate	MONK	0.82	0.91	0.75	0.6	0.95	0.7	0.74	0.69	0.99	1
	MMD_*u*_	0.91	0.95	0.81	0.72	99	0.88	0.9	0.9	1	1
	MMD_*l*_	0.83	0.93	0.84	0.83	1	0.94	0.96	0.95	1	1
Technology	MONK	0.73	0.62	0.33	0.46	0.8	0.73	0.6	0.62	0.98	0.99
	MMD_*u*_	0.85	0.74	0.43	0.54	0.99	0.9	0.93	0.93	1	1
	MMD_*l*_	0.76	0.66	0.49	0.58	1	0.92	0.94	0.94	1	1
Healthcare	MONK	0.76	0.64	0.74	0.54	0.82	0.81	0.71	0.67	0.99	1
	MMD_*u*_	0.82	0.75	0.81	0.64	1	0.92	0.95	0.95	1	1
	MMD_*l*_	0.73	0.57	0.85	0.64	1	0.95	0.94	0.93	1	1
Financial	MONK	0.85	0.6	0.62	0.37	0.84	0.66	0.55	0.55	0.98	0.99
	MMD_*u*_	0.9	0.69	0.69	0.47	0.99	0.84	0.9	0.89	1	1
	MMD_*l*_	0.87	0.63	0.71	0.58	1	0.87	0.94	0.94	1	1

**Fig 13:**
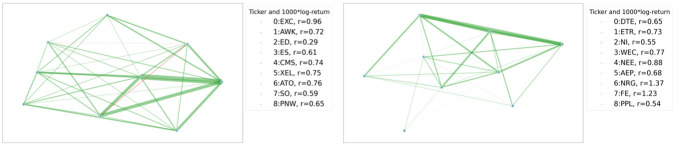
Comparison of two portfolio graphs. One graph comes from the good ESG sample and the other from the poor ESG sample, on 2019–3-15 when the test was rejected. The average log returns have been multiplied by 1000.

We can visualize the reasons for rejection by looking at two graphs, one good ESG and one poor ESG, constructed from the Utilities sector as the portfolio graphs only had 9 nodes as shown in Fig 13. The main difference, in this particular case, is that the good ESG portfolio graph was more dense and had bigger weights. Also, the good ESG portfolio only had 2 negative edges while the poor ESG portfolio has none. The average log return is higher for the bad ESG portfolio, 0.00068 vs 0.00082.

**Fig 14:**
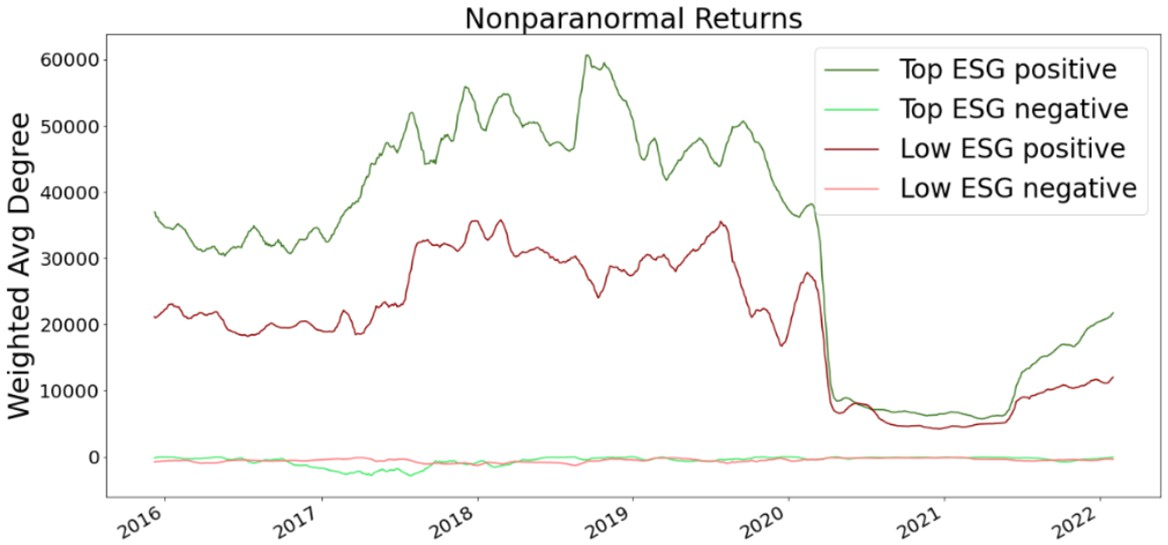
The average weighted degree of the graphs within the two portfolios. A separate degree is calculated for the negative and positive weights.

Furthermore, Fig 14 shows the average weighted average degree for the negative weights and the positive weights and the density for the graphs of the Utilities sector.

**Fig 15:**
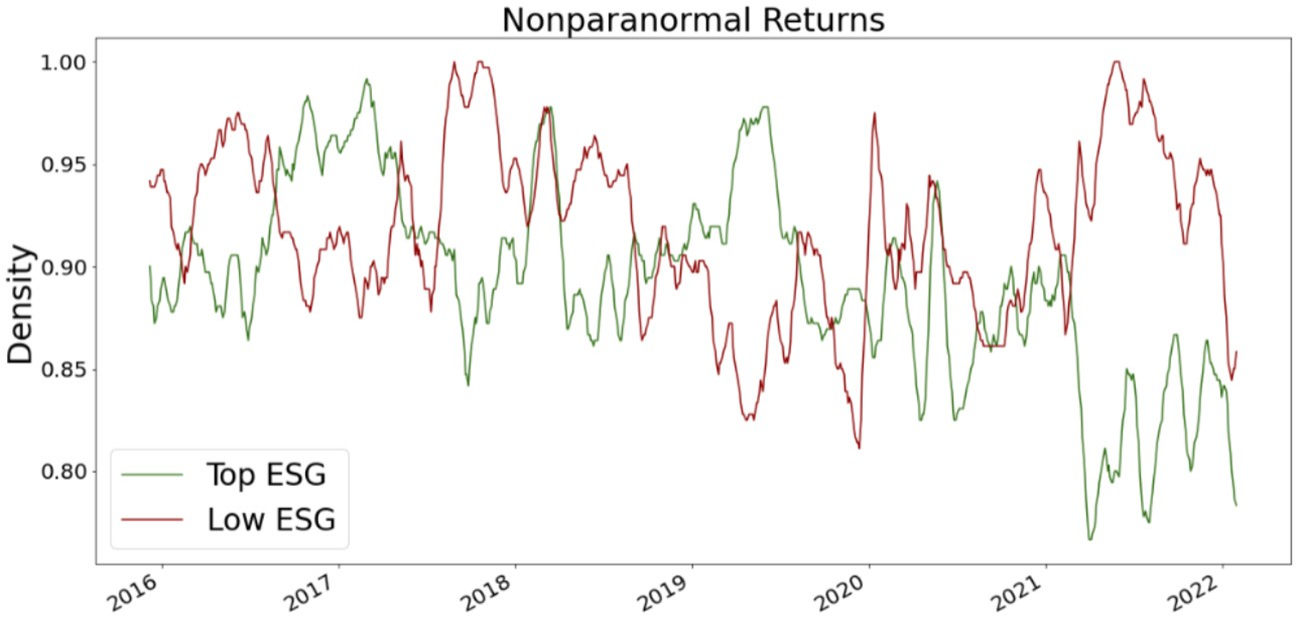
The density of the graph sample ignoring the signs and assuming a binary structure. The density is shown for the top ESG portfolio and the low ESG portfolio.

From the figure, we can see that there are very few negative edges and that the weighted average degree defined as:

$$\bar{k}=\frac{1}{\left|V\right|}\sum_{i\in V}k_{i}$$

where $k_{i}=\sum_{j\in N(v_{i})}w_{ij}$ and *w*_*ij*_ is the weight of the edge (*v*_*i*_, *v*_*j*_) are very different between the two portfolios. Interestingly, the density seen in Fig 15 between the graphs in the two samples is much more similar in value and the two two time series often cross. This is reflected in the fact that the RW kernel has more rejections as it corresponds to testing for weighted edges compared to the WL kernel which corresponds to considering only binary edges.

A natural question is whether the rejection rates are purely based on inter-asset relations or if they are related to portfolio ESG scores or portfolio performance metrics. We now mostly focus on the RW kernel as it gives good power while it can also explicitly deal with real weighted graphs (positive and negative).

**Remark**. *The negative weights in* Fig 14 *are very small in absolute value when compared to the positive weights, we also note that the number of negative edges is few. This is true for all sectors. Although negative weights are observed in sector portfolios we ignore the weights as the ratio of negative weights is very low and often 0 for different time points t. This fact can lead to problems as the negative G*^(−)^ *graphs will be empty, leading to a kernel evaluation of zero for some cases which will ruin the inference procedure if too many elements of* **K** *are zero. Therefore, we decided to ignore weights in kernels that do not deal explicitly with negative weights (all but the RW kernels) by taking the absolute value. We should also note that the attributes are of order* 10^−4^ *while the edge weights are of order* 10^3^. *For the RW kernel with attributes, we multiplied the attributes by 1000 to ensure the positive semi-definiteness of the kernel. The SP kernels use a binning strategy with the identity kernel as a base kernel to speed up the kernel calculations. Each edge weight has been divided by the maximum absolute value of the edge weights in the sample and each node attribute has been divided by the maximum absolute value of the node attributes in the sample. We did not find much difference in binning the attributes for the WL-type kernel vs using the degree as a label, but the binning strategy was usually more sensitive and rejected the test more often*.

**Fig 16:**
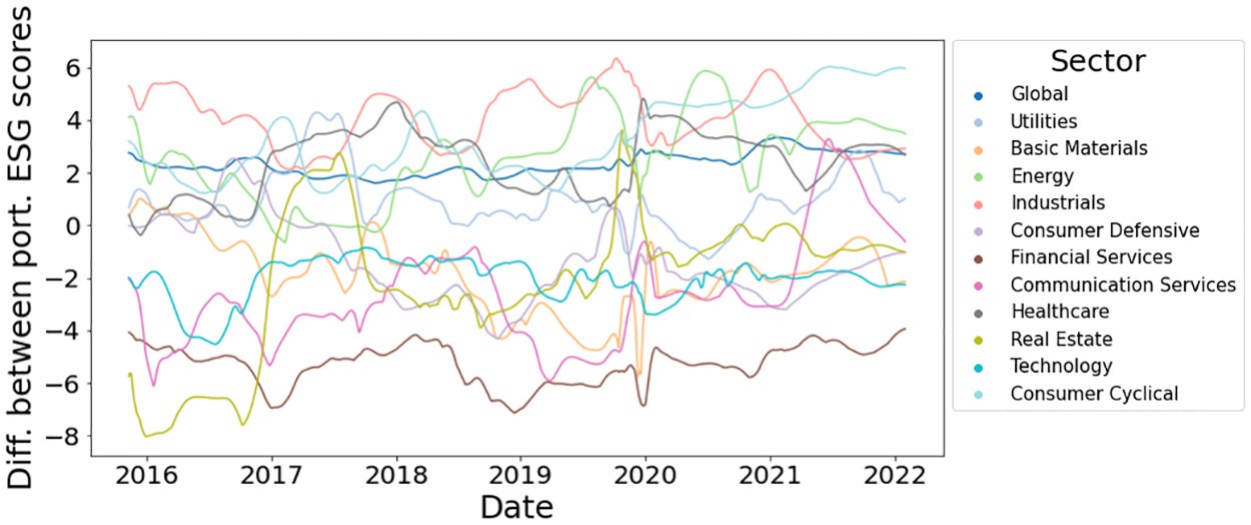
Difference of ESG scores for each sector. The figure shows *z*_*t*,*i*_, the difference between the 75% quantile of the ESG score of the good ESG portfolio and the 25% quantile of the poor ESG portfolio.

**Fig 17:**
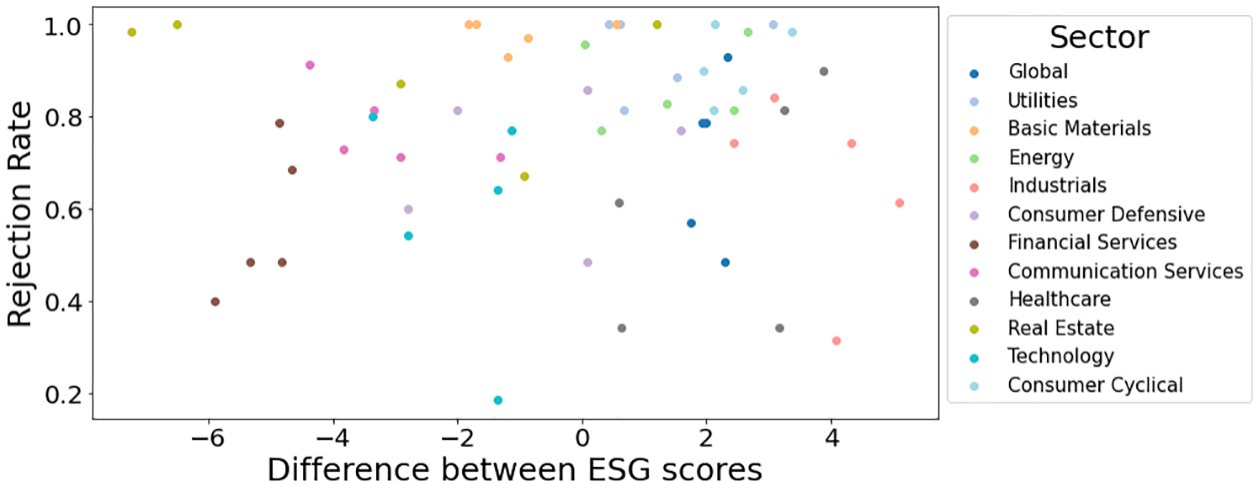
Rejection rates as a function of ${\bar{z}}_{s,i}$, the average quantile difference of the good and poor ESG portfolios. The rejection rates are the mean observed rejection in the interval[*t*_*s*_, …, *t*_*s*+*L*_].

To analyze if ESG scores affect inter-asset relations we define the following feature:

$$\begin{array}{c} \begin{array}{cc} z_{t,i} & ={\bar{e}}_{t,i}^{\left(g\right)}+2s_{t,i}^{\left(g\right)}-\left({\bar{e}}_{t,i}^{\left(b\right)}-2s_{t,i}^{\left(b\right)}\right),\\ {\bar{z}}_{s,i} & =\frac{1}{L}\;\sum_{t\in [t_{s},\cdots ,t_{s+L}]}^{L}\;z_{t,i}, \end{array} \end{array}$$

where ${\bar{z}}_{s,i}$ is the average over the time points observed in [*t*_*s*_, …, *t*_*s*+*L*_], *i* is the asset universe (sectors or global), ${\bar{e}}^{\left(g\right)}$ is the average of ESG score of the assets that make up the good portfolio and $s_{t,i}^{\left(g\right)}$ is the standard deviation. ${\bar{e}}^{\left(b\right)}$ and $s_{t,i}^{\left(b\right)}$ are defined similarly. *z*_*i*,*t*_ can be seen as the difference between the 75% quantile of the ESG score of the good ESG portfolio and the 25% quantile of the poor ESG portfolio. From Fig 16 we can observe that for some sectors the difference between the quantiles of the good and poor portfolios is negative, meaning that the ESG scores of the assets within the good and poor portfolios are similar, this is, for example, true for the Financial, Communication Services, Technology and Materials sectors. For other asset universes, the ESG profiles show differences such as the Global, Industrials, and Energy sectors. From Fig 17 we can see that there is no evidence between rejections rates and ESG differences.

Table 4 gives the occurrence ratio when the good ESG portfolio gave better performances for each portfolio type for each test decision. The exact formula is:

$$\begin{array}{c} \begin{array}{cc} {\text{P}}_{\text{rejected}} & =\frac{\sum_{i\in R}\;\sum_{t=(i-20)}^{20}\;\sum_{m_{i-t}\in M}\;1[m_{i-t}^{\left(g\right)}\;\text{better}\;\text{that}\;m_{i-t}^{\left(b\right)}]}{20|R||M|},\\ {\text{P}}_{\text{rejected},m} & =\frac{\sum_{i\in R}\;\sum_{t=(i-20)}^{20}\;1[m_{i-t}^{\left(g\right)}\;\text{better}\;\text{that}\;m_{i-t}^{\left(b\right)}]}{20|R|}, \end{array} \end{array}$$

where *R* is the set containing the time when the test was rejected using the MONK estimator and *M* = {DRatio, DVar, S, MDD, Ω, ST, TR} is the set of portfolio performance metrics and $m_{t}^{\left(g\right)}$ is the observed metric value at time *t* for the good ESG portfolio while $m_{t}^{\left(g\right)}$ is the observed value at time t for the poor ESG portfolio. *P*_not rejected_ is defined analogously but considers the set $\tilde{R}$ which contains the time when the test was not rejected using the MONK estimator. P_rejected,*m*_ considers a specific portfolio metric. We present the results for the RW kernel as the values are consistent over different kernels. The full table can be seen in the S1 Appendix.

**Table 4:** The occurrence ratio when the good ESG portfolio gave better performances for each portfolio type for each test decision. The results are using the MONK estimator from the RW kernel.

	PEW		GMV		MS	
	Not Rejected	Rejected	Not Rejected	Rejected	Not Rejected	Rejected
Global	0.67	0.75	0.57	0.53	0.4	0.41
Materials	0.79	0.86	0.74	0.86	0.62	0.79
Utilities	0.46	0.65	0.5	0.56	0.36	0.7
Industrials	0.79	0.77	0.76	0.7	0.68	0.61
Energy	0.65	0.92	0.44	0.61	0.59	0.68
Communication	0.65	0.44	0.53	0.4	0.55	0.5
Cyclical	0.33	0.25	0.37	0.38	0.31	0.31
Defensive	0.57	0.72	0.57	0.7	0.55	0.64
Real Estate	0.14	0.15	0.23	0.37	0.27	0.4
Technology	0.65	0.64	0.39	0.35	0.66	0.62
Healthcare	0.37	0.26	0.53	0.49	0.4	0.34
Financial	0.89	0.88	0.66	0.7	0.75	0.72

From Table 4 we first see that there is not an apparent difference between the ratios for the rejected and non-rejected cases. For the Global portfolio, we can see that the good ESG companies are preferred for index investing, namely the PEW portfolio, but not preferred for MS optimized portfolio while the good ESG GMV portfolio is slightly preferred over the poor ESG GMV portfolio. Then once we look at the sectors we can see that the good ESG portfolios, independent of the construction method, are most often preferred for the Materials, Industrials, Energy, Technology, and Financial sectors while the poor ESG portfolios are more often preferred for the Consumer Cyclical, Real Estate, and Healthcare. For other sectors, we usually have that the good ESG portfolio is slightly preferred using the aggregated proportion metric P_rejected_. Looking at each portfolio construction: 1) For index investing (PEW) the Consumer Cyclical, Real Estate sector and Healthcare sector show a preference for the poor ESG portfolio, 2) The poor ESG portfolios are preferred for Consumer Cyclical, Real Estate using the GMV portfolio construction and 3) Looking at the MS construction type it can be seen that the poor ESG portfolios consisting of global, Consumer Cyclical, Real Estate, and Healthcare assets outperformed the good ESG portfolio most of the time. One reason why the poor ESG Real Estate portfolio outperforms the good ESG part is that the ESG scores within the Real Estate sector are very similar, although, this is not the case for the Consumer Cyclical sector. The ESG score of the two portfolios over time can be found in the S1 Appendix. We can break down the analysis further by looking at P_rejected,*m*_. In general, the risk-adjusted returns, Sharpe, Treynor and Sortino, and omega usually agree on the ordering. Interestingly, if DRatio is better for the good ESG portfolio then DVaR is not necessarily better as well. This is, for example, the case for the global portfolio comparison. A table for P_rejected,*m*_ can be found in the S1 Appendix.

We have identified that there are structural differences between the two portfolios (the ratios in Table 4 are not all 0.5), so the natural question is whether these differences are manifested in portfolio-level performance. We start by looking at PCA biplots and then move on to logistic regression to try to identify which metrics show relations with the inter-asset connection. We construct the data as follows:

$$\begin{array}{c} \begin{array}{cc} x_{i,t} & =\frac{\sum_{k=(t-20)}^{t}\;z_{i,k}^{\left(g\right)}-z_{i,k}^{\left(b\right)}}{20},\;\;{\tilde{x}}_{i,t}=\frac{x_{i,t}-{\hat{\mu }}_{x_{i}}}{{\hat{\sigma }}_{x_{i}}}, \end{array} \end{array}$$

where *t* is a timepoint where a rejection decision is made, $z_{i,k}^{\left(g\right)}$ is the portfolio metric value for one particular portfolio construction (PEW, GMV, or MS) for the good ESG portfolio. $z_{i,k}^{\left(b\right)}$ is defined analogously but for the poor ESG portfolio. We then scale each feature and create ${\tilde{x}}_{i,t}$ where ${\hat{\mu }}_{x_{i}}$ and ${\hat{\sigma }}_{x_{i}}$ are the estimated mean and standard deviation of *x*_*i*_ respectively. The label *y*_*t*_ is the rejection decision according to the MONK estimator at time *t*, 0 if no rejection and 1 otherwise.

**Fig 18:**
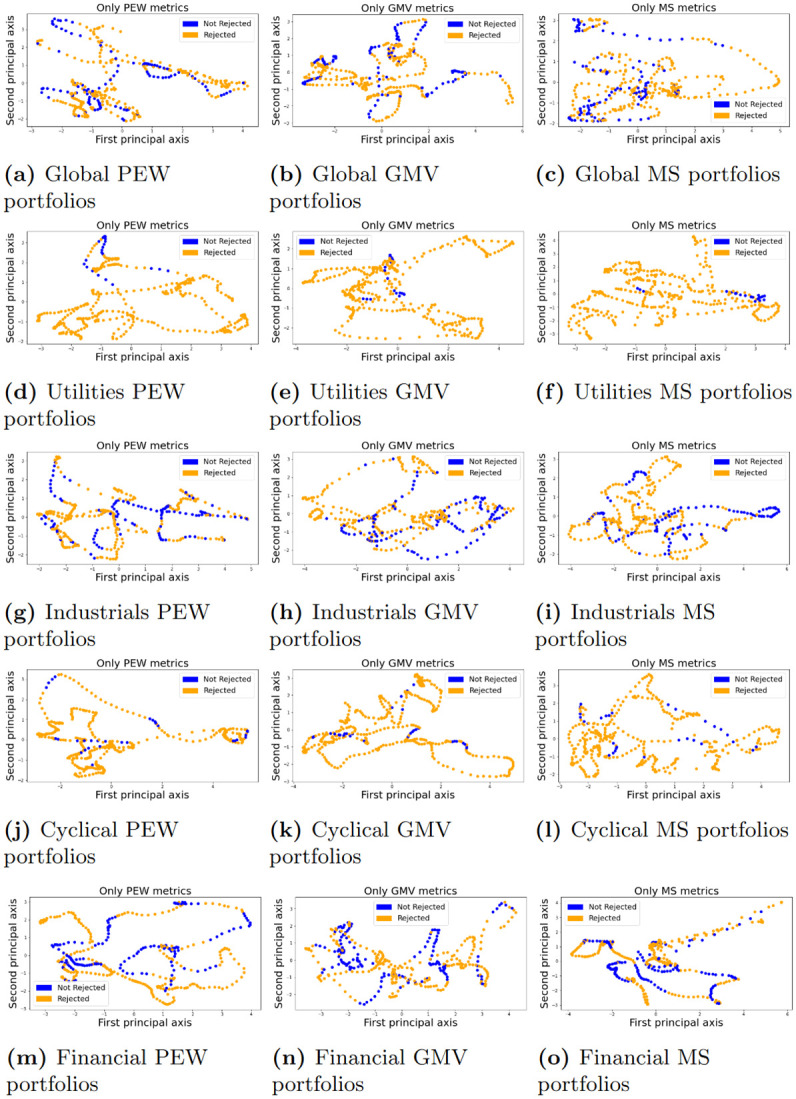
PCA biplots for a few asset universes. The orange dots indicate time points when a rejection was made while the orange dots indicate no rejection.

Fig 18 shows a PCA biplot using the RW kernel for a few sectors. A figure for all sectors can be found in the S1 Appendix. The non-rejections tend to occur in the neighborhood of other rejections, although the two classes are not linearly separable. We now perform a logistic regression whose objective is to classify a MONK rejection when the input data are portfolio metrics. We used a weighted logistic regression to adjust for the class imbalance defined as:

$$\begin{array}{c} \text{log-loss}=\sum_{i=1}^{n}w_{0}y_{k}\;\text{log}\left(p\left(x\right)\right)+w_{1}\left(1-y_{k}\right)\;\text{log}\left(1-p\left(x\right)\right)+\lambda \sum_{j=1}^{p}\left|\beta_{j}\right|, \end{array}$$

where *w*_0_ and *w*_1_ are the class weights for non-rejection and rejection labels, and $p\left(x\right)=\frac{1}{1-\text{exp}\;(\beta^{T}x)}$ using **x** as features and ***β*** is the vector of regression coefficients.*y*_*k*_ is the rejection decision in test nr. *k*. *n* is the number of tests performed and *p* is the number of features in the regression (number of portfolio constructions times the number of portfolio metrics). The regularization parameter λ is found by using a 3-fold CV. The data matrix was shuffled randomly before the estimation procedure began to evenly spread the non-rejections over each fold. The weights were found by using $w_{i}=\frac{n}{2n_{i}}$ where *n*_*i*_ is the number of observations in class *i*. Table 5 lists the area under the curve (AUC) for each fold and its corresponding estimated 95% confidence interval (CI). The column all metrics means that **x** contains all metrics for each portfolio optimization method 3*7 = 21 in total, while the columns PEW, GMV, and MS metrics mean that the logistic regression input **x** contained only metrics from the specific portfolio optimization mentioned in the column header. The table demonstrates that it is possible to relate, in all cases, the outcome of the test related to structural changes between portfolios with high vs. low ESG scores and financial performance. The table shows that there is a relationship between performance and ESG scores independent of the strategy of type. The key finding is that whatever strategy is used, there will be a difference by considering ESG. Because portfolio performance implies a difference in inter-asset relations measured between high and low ESG portfolios. Furthermore, the fact that the AUC is consistent over sectors, portfolio construction methods, and kernel types, we deduce that there are no special cases where ESG does not matter when one looks at inter-asset relations. We further confirmed these results by performing a support vector machine (SVM) classification to add non-linear relations. The AUC increases for all cases, quite significantly for some, indicating that there are also further non-linear relations. The table for the SVM can be found in the S1 Appendix.

**Table 5:** AUC of the logistic regression trying to classify a MONK non-rejection when the input data were portfolio metrics. The AUC and its 95% confidence interval were estimated using a 3-fold CV. The column all metrics means that **x** contains all metrics for each portfolio optimization while the columns PEW, GMV, and MS metrics mean that the logistic regression input **x** contained only metrics from the specific portfolio optimization mentioned in the column header.

	Kernel\ AUC	All metrics	PEW metrics	GMV metrics	MS metrics
Global	RW	0.88±0.06	0.82±0.08	0.73±0.12	0.73±0.12
	RW attr.	0.93±0.04	0.9±0.04	0.85±0.06	0.85±0.06
	prop.	0.79±0.05	0.75±0.09	0.63±0.03	0.63±0.03
	SP	0.78±0.05	0.72±0.1	0.68±0.07	0.68±0.07
	WL	0.71±0.18	0.58±0.06	0.43±0.04	0.7±0.1
Materials	RW	0.93±0.05	0.88±0.06	0.78±0.2	0.75±0.3
	RW attr.	0.98±0.03	0.84±0.2	0.84±0.14	0.84±0.14
	prop.	0.73±0.2	0.68±0.07	0.65±0.15	0.44±0.03
	SP	0.88±0.08	0.83±0.08	0.87±0.1	0.76±0.04
	WL	0.91±0.02	0.81±0.06	0.75±0.07	0.77±0.02
Utilities	RW	0.98±0.02	0.94±0.06	0.96±0.02	0.96±0.01
	RW attr.	0.97±0.07	0.84±0.2	0.84±0.14	0.84±0.14
	prop.	0.84±0.04	0.75±0.04	0.76±0.04	0.76±0.1
	SP	0.96±0.001	0.94±0.02	0.89±0.02	0.9±0.03
	WL	0.79±0.04	0.67±0.06	0.67±0.09	0.62±0.02
Industrials	RW	0.86±0.03	0.7±0.11	0.71±0.06	0.74±0.06
	RW attr.	0.95±0.07	0.9±0.09	0.83±0.13	0.77±0.15
	prop.	0.92±0.1	0.89±0.1	0.79±0.15	0.72±0.11
	SP	0.84±0.04	0.74±0.02	0.8±0.07	0.75±0.03
	WL	0.77±0.06	0.65±0.1	0.67±0.05	0.71±0.07
Energy	RW	0.93±0.03	0.9±0.04	0.75±0.07	0.81±0.06
	RW attr.	0.92±0.03	0.86±0.02	0.77±0.04	0.8±0.03
	prop.	0.88±0.01	0.83±0.08	0.64±0.12	0.82±0.06
	SP	0.83±0.05	0.79±0.05	0.69±0.04	0.83±0.05
	WL	0.73±0.04	0.68±0.08	0.6±0.13	0.63±0.07
Communication	RW	0.85±0.05	0.82±0.12	0.77±0.14	0.76±0.06
	RW attr.	0.86±0.07	0.71±0.12	0.66±0.24	0.68±0.15
	prop.	0.78±0.06	0.75±0.08	0.75±0.07	0.7±0.05
	SP	0.79±0.04	0.7±0.09	0.63±0.15	0.58±0.07
	WL	0.81±0.07	0.71±0.1	0.72±0.06	0.77±0.05
Cyclical	RW	0.89±0.07	0.76±0.11	0.76±0.02	0.76±0.11
	RW attr.	0.81±0.5	0.67±0.24	0.64±0.36	0.72±0.34
	prop.	0.92±0.02	0.85±0.01	0.88±0.01	0.76±0.01
	SP	0.91±0.006	0.86±0.06	0.69±0.05	0.85±0.06
	WL	0.69±0.04	0.62±0.11	0.6±0.04	0.61±0.08
Defensive	RW	0.87±0.04	0.66±0.02	0.75±0.08	0.77±0.03
	RW attr.	0.91±0.05	0.79±0.09	0.76±0.04	0.87±0.1
	prop.	0.78±0.08	0.73±0.05	0.76±0.07	0.72±0.13
	SP	0.85±0.11	0.73±0.11	0.72±0.12	0.56±0.14
	WL	0.73±0.06	0.72±0.12	0.72±0.1	0.63±0.02
Estate	RW	0.91±0.03	0.92±0.02	0.87±0.04	0.82±0.08
	RW attr.	0.67±0.03	0.53±0.12	0.6±0.1	0.63±0.12
	prop.	0.96±0.02	0.9±0.11	0.83±0.04	0.64±0.15
	SP	0.76±0.03	0.68±0.06	0.68±0.04	0.66±0.12
	WL	0.56±0.01	0.7±0.04	0.72±0.04	0.67±0.02
Technology	RW	0.82±0.14	0.64±0.14	0.75±0.02	0.69±0.15
	RW attr.	0.89±0.08	0.67±0.07	0.81±0.03	0.75±0.11
	prop.	0.85±0.08	0.82±0.04	0.81±0.04	0.73±0.1
	SP	0.85±0.02	0.7±0.02	0.74±0.05	0.57±0.07
	WL	0.72±0.06	0.65±0.04	0.69±0.06	0.71±0.13
Healthcare	RW	0.86±0.09	0.69±0.11	0.81±0.08	0.68±0.03
	RW attr.	0.89±0.02	0.83±0.04	0.82±0.03	0.79±0.06
	prop.	0.9±0.03	0.88±0.06	0.79±0.1	0.77±0.15
	SP	0.72±0.04	0.62±0.01	0.67±0.04	0.72±0.06
	WL	0.87±0.04	0.83±0.08	0.78±0.03	0.79±0.09
Financial	RW	0.85±0.06	0.69±0.06	0.74±0.05	0.73±0.06
	RW attr.	0.91±0.04	0.75±0.1	0.74±0.04	0.85±0.08
	prop.	0.91±0.07	0.8±0.09	0.83±0.11	0.74±0.07
	SP	0.8±0.06	0.7±0.12	0.6±0.08	0.53±0.08
	WL	0.8±0.07	0.67±0.08	0.67±0.12	0.7±0.07

These findings were analyzed further by looking at which features are affected by the difference in inter-asset correlations of high and low ESG portfolios. Table 6 shows which metrics remained in the lasso logistic regression using the 1 standard deviation rule [99]. First, we can see that there are some features that are almost always selected. These are the MDD, DRatio, and Treynor from independent from the construction, although the PEW features are most often observed.

**Table 6:** The table shows which metrics remained in the lasso logistic regression model using all metrics available. TR = Treynor, S = Sharpe, ST = Sortino, MDD = maximum drawdown, the subscript indicates which portfolio construction the metric came from.

	Kernel	DRatio_*pew*_	DVar_*pew*_	S_*pew*_	MDD_*pew*_	Ω_*pew*_	ST_*pew*_	TR_*pew*_	DRatio_*gmv*_	DVar_*gmv*_	S_*gmv*_	MDD_*gmv*_	Ω_*gmv*_	ST_*gmv*_	TR_*gmv*_	DRatio_*ms*_	DVar_*ms*_	S_*ms*_	MDD_*ms*_	Ω_*ms*_	ST_*ms*_	TR_*ms*_
Global	RW	x	x	x	x				x			x								x		
	RW attr.	x	x	x	x		x	x		x		x				x	x			x	x	x
	Prop	x	x	x	x	x	x	x	x	x		x		x	x		x	x		x	x	x
	SP	x	x		x		x	x	x	x	x	x				x	x		x	x	x	x
	WL		x	x	x		x		x			x		x	x		x	x	x	x	x	x
Utilities	RW	x							x			x					x	x	x			x
	RW attr.	x			x				x	x		x						x				x
	Prop		x		x			x		x		x							x	x		
	SP	x	x		x		x	x		x	x	x			x	x	x		x	x		
	WL	x	x	x	x			x	x	x		x	x		x	x		x	x			x
Materials	RW	x	x		x			x				x	x				x					x
	RW attr.								x						x		x				x	x
	Prop	x	x		x			x		x					x		x			x		x
	SP		x		x			x	x								x	x				
	WL	x	x		x	x		x	x	x	x	x				x	x		x	x		x
Industrial	RW	x	x	x	x	x		x	x	x		x		x	x		x		x	x		
	RW attr.	x	x		x					x		x	x			x						
	Prop	x	x			x		x			x					x	x		x		x	
	SP	x			x		x	x	x	x	x			x	x	x	x	x	x	x	x	x
	WL	x				x			x	x					x	x			x			x
Energy	RW		x		x	x		x		x	x	x				x	x		x			x
	RW attr.	x	x		x		x			x	x	x			x	x	x		x	x		x
	Prop	x	x	x	x			x	x	x	x				x	x	x	x	x			x
	SP	x	x		x	x		x							x	x				x		x
	WL	x	x	x	x			x		x		x			x	x	x			x		x
Com.	RW	x	x		x	x		x		x	x	x			x	x			x		x	x
	RW attr.	x	x	x	x	x		x	x	x		x			x	x	x		x		x	x
	Prop		x		x			x	x	x		x					x					x
	SP	x	x		x	x		x		x	x	x				x	x		x		x	x
	WL				x				x				x			x						x
Cyclical	RW	x	x		x		x			x		x			x	x	x	x		x		
	RW attr.	x	x		x			x	x						x		x					x
	Prop	x	x		x			x	x			x	x		x	x	x	x	x			x
	SP	x	x		x	x		x	x	x	x	x			x	x	x	x	x	x	x	x
	WL	x	x		x			x	x	x		x	x		x	x	x		x		x	x
Defensive	RW	x		x	x			x	x	x		x	x		x	x		x	x	x	x	x
	RW attr.	x	x		x		x	x		x		x		x	x	x	x			x		
	Prop	x			x		x			x		x	x		x		x		x			
	SP	x			x			x	x			x		x		x						x
	WL	x	x		x				x	x		x					x		x		x	
Estate	RW	x	x		x			x	x								x		x			x
	RW attr.	x	x	x	x	x		x		x		x		x	x	x	x		x	x		x
	Prop	x	x		x	x		x	x	x	x	x			x	x	x	x	x			x
	SP		x		x				x	x	x	x	x	x	x	x	x		x			x
	WL	x	x		x	x		x	x	x		x		x	x	x	x		x		x	x
Tech.	RW		x			x			x	x		x			x	x	x					x
	RW attr.	x	x		x		x		x	x	x	x	x		x	x	x	x		x		x
	Prop	x	x						x	x	x	x					x		x			x
	SP	x	x	x	x		x	x	x	x	x	x	x		x	x	x	x	x			
	WL	x	x		x	x		x	x			x		x	x	x			x		x	x
Health.	RW	x			x				x	x		x			x	x		x	x			
	RW attr.	x	x	x	x	x		x	x	x		x		x	x	x	x		x	x		x
	Prop	x	x	x	x	x		x	x	x		x			x	x	x	x	x		x	x
	SP	x	x	x	x			x	x		x	x			x	x	x	x	x	x	x	x
	WL	x	x		x			x		x						x			x			
Financial	RW				x				x	x	x	x	x			x			x			x
	RW attr.		x		x	x		x	x	x		x	x			x			x			
	Prop	x	x	x	x	x			x	x	x	x	x			x	x		x			
	SP	x	x	x					x			x				x			x		x	x
	WL	x	x	x	x			x	x	x		x	x						x	x		x

**Fig 19:**
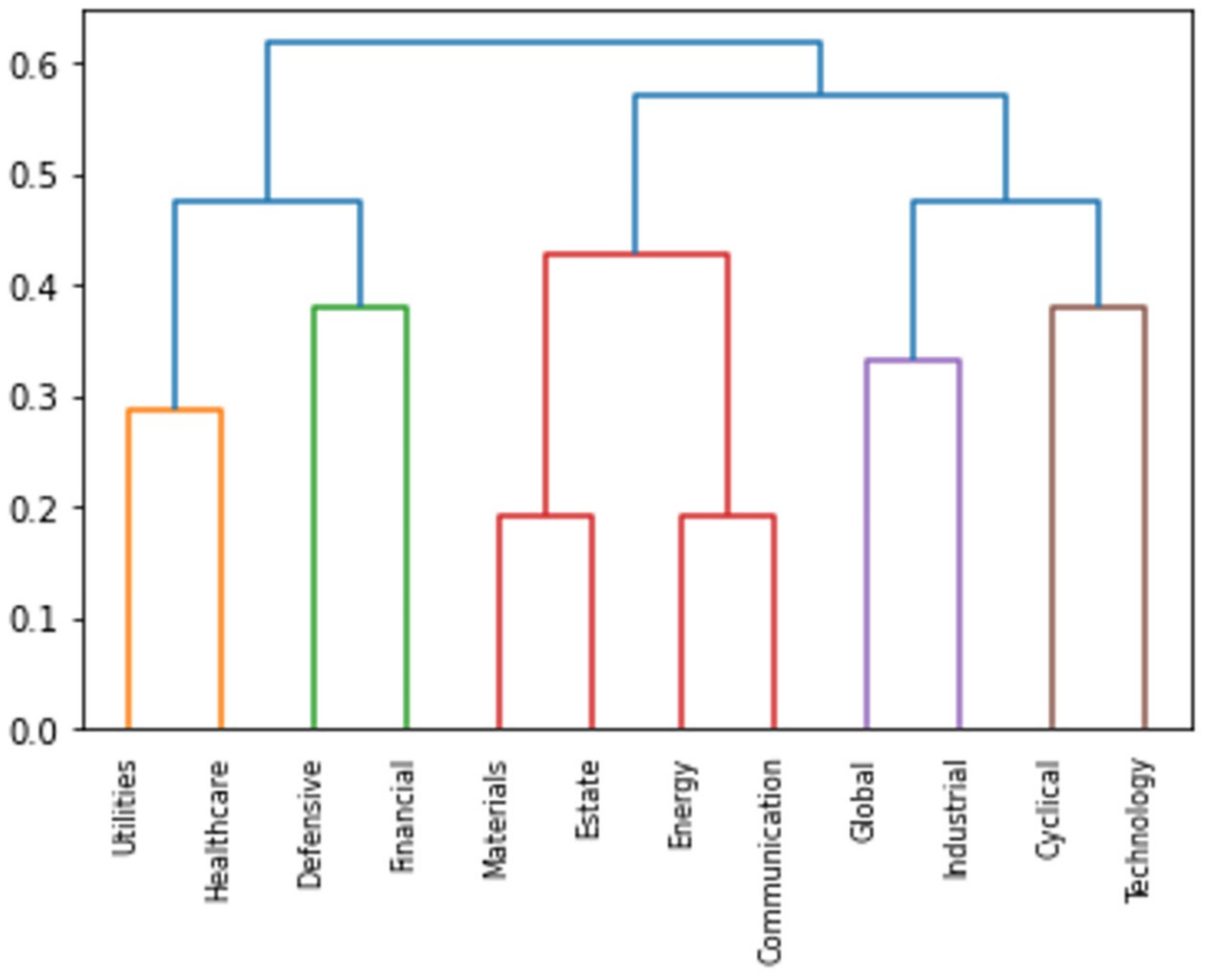
The normalized hamming distance between the feature sets for each sector on the RW kernel labels.

Fig 19 shows the normalized hamming distance between the feature sets for each sector on the RW kernel labels. We want to see if similar sectors are clustered together. It can be seen that we can cluster some sectors together, such as the Energy and Communication Services and the Real Estate and Basic Materials, although the reason for their similarity is not obvious.

## 8 Conclusion

In this manuscript, we took a novel approach to study and quantize differences in inter-asset relations and graph structures between portfolios with different ESG profiles. We used graph kernels to quantify the differences and identified differences in the analysis introduced by different graph kernels both in a simulation study and a real portfolio optimization scenario. We further introduced a robust estimator for such tests which are of great importance for the financial literature. We showed empirically that investing in a good ESG Investment-fund (PEW portfolio) does outperform its poor ESG counterpart for a global asset universe and for most sectors. However, once a max Sharpe optimization is allowed, then a poor ESG portfolio can outperform the good ESG portfolio counterpart. This is for example the case for the global asset universe. We note, however, that this is the result of an aggregated study over a 5-year history. Finally, we showed that given the kernel MMD rejections it is possible to use the difference between portfolio performance metrics to guess the rejection decision at a given time with high accuracy. Although this relationship is not obvious a lasso regression suggested that the MDD, DRatio, and Treynor metrics are the most informative. A non-linear classification was also performed giving an even higher AUC.

## Supporting information

S1 Appendix File containing pseudo code of algorithms, additional information on the labelled random walk kernel, and synthetic experiments. (PDF)
