# Supplementary material for: Assessing portfolio diversification via two-sample graph kernel inference. A case study on the influence of ESG screening
Source: PLoS One. 2024 Apr 16;19(4):e0301804. doi: 10.1371/journal.pone.0301804 (PMC11020627; doi:10.1371/journal.pone.0301804)
Supplement: S1 Appendix — (PDF) [file pone.0301804.s001.pdf]

Supplementary appendix - Assessing portfolio diversification via two-sample graph kernel inference.
A case study on the influence of ESG screening

Ragnar L. Gudmundarson[1†], Gareth W. Peters[2†],

1 Centre for Networks & Enterprise, Edinburgh Business School, Heriot-Watt University, Edinburgh, United Kingdom
2 Department of Statistics & Applied Probability, University of California, Santa Barbara Santa Barbara, California, United States

†These authors contributed equally to this work.
* rlg2000@hw.ac.uk

Contents

1 Approximation of p-value using permutation 1

2 Approximation of Edge Labelled Random Walk Kernel 3

3 Gaussian Process Smoothing using Stratified Cross Validation 4

4 Portfolio Construction and MMD Testing 5

5 Graph Experiments 6
5.1 Binomial Graphs . . . 6
5.2 Scale Free Graphs . . . 8
5.3 Stochastic Block Model with Different Label Distribution . . . 9
5.4 Stochastic Block Model with Different Block Matricies . . . 10
5.5 Signed Networks . . . 11
5.6 Robust Graph Testing . . . 14

6 Portfolio Metrics 15

7 PCA Analysis 16

8 SVM results 19

9 Rejection Rate per Portfolio Type and Sector 20

1 Approximation of p-value using permutation

To approximate the p-value of the mmd test statistic algorithm 1 may be used.

Algorithm 1: Estimate p-value of an i.i.d sample

```
input : K - Kernel matrix
        B - Number of permutations
        n - Number of elements in sample 1
        m - Number of elements in sample 2
        MMD - A function that estimates the MMD statistic

output: The p-value p


T_sample ← MMD(K, n, m)   // Start by calculating the MMD of the current sample

// Initialize empty array and repeat the MMD calculation
T ← array(b)

for b ∈ 1 : B do
    K_b ← array(n+m, n+m)
    index = permutation(n + m)   // index is a new permutation of the vector
        [1, 2, ..., n + m]

    for i ∈ 1 : (n + m) do
        for j ∈ 1 : (n + m) do
            K_b[i, j] = K[index[i], index[j]]
        end
    end

    T[b] = MMD(K_b, n, m)
end
p ← Σ_b 1(T_sample > T[b]) / B
```

$$p \leftarrow \frac{\sum_b \mathbb{1}(T_{sample} > T[b])}{B}$$

2 Approximation of Edge Labelled Random Walk Kernel

Following [1] take $\mathcal{H} = \mathbb{R}$ and $\Phi(\mathbf{X}) = \mathbf{A}$ where $\mathbf{A}$ is the adjacency matrix of G then $\mathbf{W}_\times = \mathbf{A}_\times$. This means that $< \phi(X_{ij}), \phi(X'_{kl}) >_{\mathcal{H}} = < A_{ij}, A'_{kl} >_{\mathbb{R}} = A_{ij}A'_{kl}$. Similarly, if $\phi(X_{ij}) = 1$ if $(v_j, v_j) \in E$ then $\mathbf{W}_\times = \tilde{\mathbf{A}}_\times$ as the degrees of nodes in $G_\times$ is the product of the degree of the nodes they are made up of. We can make the labeling more abstract, for example, if the edges take labels on a finite set $1, 2, \ldots, d$ and we let $\mathcal{H} = \mathbb{R}^d$. Define $\phi(X_{ij}) = e_l$ if edge (v_j, v_i) is labelled l and 0 otherwise. Then the weight matrix $W_\times$ can be written as.

$$\mathbf{W} = \sum_{l=1}^{d} {}^l\,\mathbf{A} \otimes {}^l\,\mathbf{A}'\,.$$

The geometric random walk kernel is:

$$K(G, G') = \mathbf{q}^T (\mathbf{I} - c\,\mathbf{W}_\times)^{-1}\,\mathbf{p}\,.$$

To approximate the geometric random walk kernel we follow [2]. The approximation does a r eigen decomposition of each ${}^l\,\mathbf{A}$, that is ${}^l\,\mathbf{A} = {}^l\,\mathbf{U}\,{}^l\,\mathbf{\Lambda}\,{}^l\,\mathbf{U}^T$ and then we calculate:

$$\begin{aligned}
&\mathbf{q}^T (\mathbf{I} - c\,\mathbf{W}_\times)^{-1}\,\mathbf{p} \\
&\quad = \mathbf{q}^T (\mathbf{I} - c \sum_{l=1}^{d} ({}^l\,\mathbf{U}_1 \otimes {}^l\,\mathbf{U}_2)({}^l\,\mathbf{\Lambda}_1 \otimes {}^l\,\mathbf{\Lambda}_2)({}^l\,\mathbf{U}_1^T \otimes {}^l\,\mathbf{U}_2^T))^{-1}\,\mathbf{p} \\
&\quad = \mathbf{q}^T (\mathbf{I} - c\,\mathbf{U}\,\mathbf{\Lambda}\,\mathbf{U}^T)^{-1}\,\mathbf{p} \\
&\quad = \mathbf{q}^T (\mathbf{I} + c(\mathbf{U} \otimes \mathbf{U})\tilde{\mathbf{\Lambda}}(\mathbf{U}^T \otimes \mathbf{U}^T))\,\mathbf{p} \\
&\quad = (\mathbf{q}_1^T\,\mathbf{p}_1)(\mathbf{q}_2^T\,\mathbf{p}_2) + c\Big(\sum_l (\mathbf{q_1}\,{}^l\,\mathbf{U}_1 \otimes \mathbf{q_2}\,{}^l\,\mathbf{U}_1)\Big)\tilde{\mathbf{\Lambda}}\Big(\sum_l ({}^l\,\mathbf{U}_1^T\,\mathbf{p}_1 \otimes {}^l\,\mathbf{U}_2^T\,\mathbf{p}_2)\Big),
\end{aligned}$$

where $\mathbf{U} = [({}^1\,\mathbf{U}_1 \otimes {}^1\,\mathbf{U}_2), \ldots, ({}^d\,\mathbf{U}_1 \otimes {}^d\,\mathbf{U}_2)]$, $\mathbf{\Lambda} = \mathrm{diag}\big(({}^1\,\mathbf{\Lambda}_1 \otimes {}^1\,\mathbf{\Lambda}_2), \ldots, ({}^d\,\mathbf{\Lambda}_1 \otimes {}^d\,\mathbf{\Lambda}_2)\big)$ and $\tilde{\mathbf{\Lambda}} = \big(\mathbf{\Lambda}^{-1} - c\,\mathbf{I}\big)^{-1}$ the third equality is obtained using the Sherman-Woodbury-Morrison lemma [3]. The top r eigendecomposition is $O(|E|r)$. Since $\mathbf{\Lambda}$ is a diagonal matrix of size $dr^2 \times dr^2$ its inversion and calculation of $\tilde{\mathbf{\Lambda}}$ is $O(dr^2)$. Multiplying $\mathbf{q_1}\,{}^l\,\mathbf{U}_1$ is $O(|V|r)$, the size of $(\mathbf{q_1}\,{}^l\,\mathbf{U}_1 \otimes \mathbf{q_2}\,{}^l\,\mathbf{U}_1)$ is $1 \times r^2$ giving so the complexity of the matrix multiplication within the sum is $O(r^4)$. The overall complexity is $O(|V|r + |E|r + r^4)$. Note that r is a small number and $r = 6$ will usually suffice.

3 Gaussian Process Smoothing using Stratified Cross Validation

The GP smoothing was performed using stratified cross-validation as illustrated in algorithm 2.

Algorithm 2: GP estimation procedure

input : k - Kernel function
n_{cv} - Number of folds
X - Features (time)
y - Observations
sigma_array - array with values of σ to be tested

output: f - Gaussian process

$T \leftarrow \text{length}(y)$
index $\leftarrow 1 : T$
$R \leftarrow$ zero array `// Store R hat`
for $\sigma \in$ *sigma_array* **do**
  for $i \in 1 : n_{cv}$ **do**
    test_index $\leftarrow i : T : n_{cv}$ `// Every` n_{cv} `observation is used as test`
    train_index $\leftarrow$ index $\setminus$ test_index `// Train set is index without test index`
    Xtrain $\leftarrow X$[train_index]
    ytrain $\leftarrow y$[train_index]
    $f \leftarrow$ fit GP using Xtrain, ytrain and σ
    Xtest $\leftarrow X$[test_index]
    $\hat{y} \leftarrow f(Xtest)$
    $R[\sigma] \leftarrow R[\sigma] + \left(1 - \frac{\sum_{i \in \text{test\_index}} (y_i - \hat{y}_i)^2}{\sum_{i \in \text{test\_index}} (y_i - \bar{y}_i)^2}\right) / \text{length(test\_index)}$
  end
end
Select σ that gave highest $\hat{R}^2$ in the array R. Fit a model using σ on all observations and return the model

4 Portfolio Construction and MMD Testing

The overall algorithm to construct and compare the portfolios is illustrated in algorithm 3.

Algorithm 3: Portfolio MMD test

```
input : R - Data frame of returns; E - Data frame of ESG scores
        w - Graph estimation window size; d - Graph estimation rolling
        window step size
        n - Number of time points; p - Number of assets
        s - MMD testing window size; k - MMD testing rolling window step
        size

output: A - Multi array to store graphs for top and low ESG assets
P - Multi array to store portfolio measures and for top and low ESG portfolios

// Start finding if assets divisible by 3
if P%3 != 0 then
  | Remove P % 3 assets from X at random
end

// Perform rolling window graph/portfolio estimation
for i ∈ w : n : d do
  | // Sort the assets at time i according to ESG
  | E_i ← sort(E[i]) // Extract top (0), medium (1) and low (2) ESG
  |    assets
  | for g ∈ [1, 2] do
  |   | E_ig ← Extract assets belonging to ESG group g
  |   | X_ig ← X[(i − w) : i, E_ig]
  |   | X_ig ← nonparanormal transformation(X_ig)
  |   | A_ig ← glasso(X_ig) // Best regularization found with EBIC
  |   | Σ_ig ← −A_ig^{−1} // Covariance estimator
  |   | μ_ig ← mean(X_ig)// Mean for each asset
  |   | P_ig ← Get portfolio weights and performance
  | end
end

// Perform MMD test
l ← length(A) // Nr of stored graphs
pval ← array to store p-values
for i ∈ s : l : k do
  | for g_1 ∈ [0, 1, 2] do
  |   | for g_2 ∈ g_1 : 2 do
  |   |   | S_1 ← A[(i − s) : s, g_1] // Graphs in group g_1 as sample 1
  |   |   | S_2 ← A[(i − s) : s, g_2] // Graphs in group g_2 as sample 2
  |   |   | pval_{ig_1g_2} ← MMDtest(S_1, S_2)
  |   | end
  | end
end
Output A, P and pval to analyze
```

5 Graph Experiments

This Section contains multiple Experiments on graph kernels and two-sample testing. We visualize the area under the ROC curve (AUC) [4] to compare the performances of different graph kernels.

5.1 Binomial Graphs

We start off by generating the simplest of graphs, the binomial graph. The main goal of this experiment is to check how graph kernels perform when compared to a test that only considers the average degree distribution. The nodes are labeled with their corresponding degree, that is, a node with degree 5 will be labeled 5. The main reason for this labeling is to test graph kernels that take labels into account, such as the WL kernel which is only applicable to labeled graphs.

Let p be the probability that an edge exists between two nodes, then the probability that a graph G appears is given by $P(G) = p^m(1-p)^{\binom{n}{2}-m}$ and average degree k is given by:

$$k = \mathbb{E}[2\frac{m}{n}] = \frac{2}{n}\,\mathbb{E}[m] = \frac{2}{n}\binom{n}{2}p = (n-1)p,$$

where we used the fact that $2m = \sum_{i=1}^{n} k_i$. Thus the average degree can also be used to parameterize the probability distribution.

Fig 1 displays the power as a function of α, the probability of type I error for different graph kernels. We look at the case when the number of graphs in each sample is 20 and when each graph has 60 nodes, the degree of the graphs in sample 1 is 4 and the degree of the graphs in sample 2 is 4.25. The ratio of the average degrees is 1.0625. All graph kernels are unnormalized.

- For the WL kernel we can see that the AUC is more or less indifferent to the number of WL iterations. This is perhaps not so surprising for so simple graphs.
- The shortest path kernel has the best performance when the nodes are not labeled according to their degree as can be seen in the figure.
- For the WWL kernel we can see that the AUC is indifferent to the number of WL iterations. Different values for the discount factor $\{0.001, 0.01, 0.1, 1\}$ were tested and they all gave the same AUC.
- The propagation kernel has multiple parameters. For this experiment it was best to have the maximum number of propagation to be set as 2, too many propagations lowered the AUC. However, as the bin width decreased the difference in the AUC decreased. For example, when the bin width was set to 0.001, the AUC of propagation kernels with a different number of propagations was almost the same. The overall AUC was also similar to the AUC of the propagation kernel with 2 propagations and a bin width of 0.1 as shown in the figure. The outcome was indifferent to the metric used.
- The pyramid kernel has the best performance when the number of levels is around $L = 6$. In this experiment, similar results are achieved for other dimension numbers. Note that too high L will make the kernel matrix diagonally dominant. Unlike the SP kernel, the pyramid kernels have better performance when the nodes are labeled according to their degrees (rather than performing the pyramid match without any labeling.
- The number of WL iterations does not matter for the WL-OA kernel in this experiment. The VH kernel gives surprisingly good results compared to other kernels.
- The Random Walk kernel gives a good performance, especially the ARKU_plus type which is an approximation to the geometric random walk kernel. The number of eigenpairs used to approximate the kernel does not play a significant role and the result is obtained only with 2 eigenpairs, we also tried 4 and 6 eigenpairs but the results did not differ. The p-step random walk is most sensitive to the discount parameter and gave the highest power when it was set to 0.01. The number of steps used in the p-rw kernel all gave a similar performance for steps from 2 to 20, Although a 1-step random walk had the lowest power giving a similar AUC as a test based on the average degree.

Finally, a comparison of all the kernels along with a test based on the average degree can be found in Fig 2

The best test is the approximated Random Walk kernel which outperforms all other kernel tests along with the test based on average degrees. The second best test is the shortest path kernel without labeling information and the third best is the test based on average degrees The reason why a test based on the average degree gives high power is because the average degree is a sufficient statistic for random graphs. As mentioned earlier the RW kernel is also a test based on the average degrees, but by taking more than 1 step we have a test that is based on higher moments. In fact, the ROC curve of the 1-step random walk kernel is similar to the ROC curve of the average degree test. Other kernels that give a high power are the Pyramid kernel and the WL-OA kernel. The worst kernel is the VH kernel but the difference is only by a marginal.

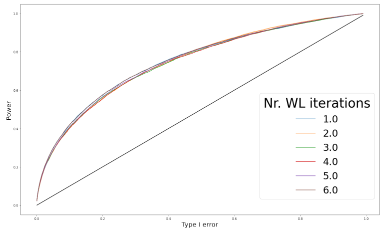

(a) The WL kernel.

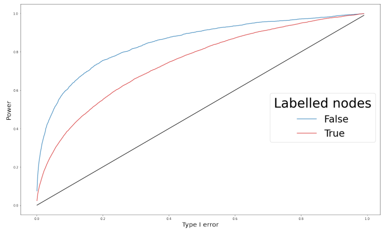

(b) The SP kernel.

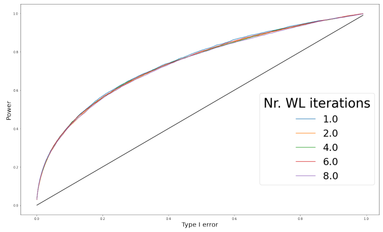

(c) The WWL kernel. The discount factor is 0.1.

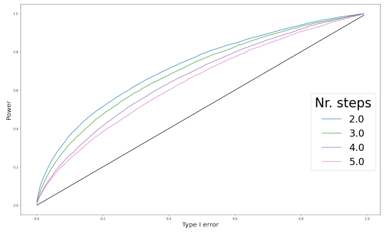

(d) The Propagation kernel. The bind width is 0.1.

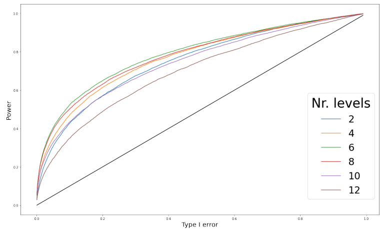

(e) The Pyramid kernel. The dimension is 2.

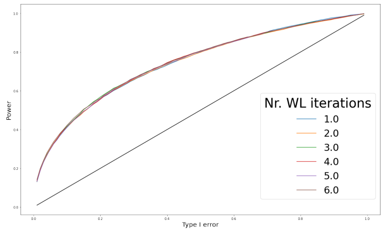

(f) The WL-OA kernel.

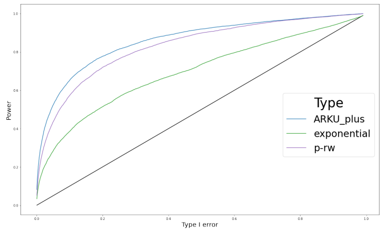

(g) The RW kernel. The number of eigenvalues used is 2 and the number of walks for the p-rw is $p = 4$.

Fig 1. ROC curves for the binomial graph experiment for different graph kernels

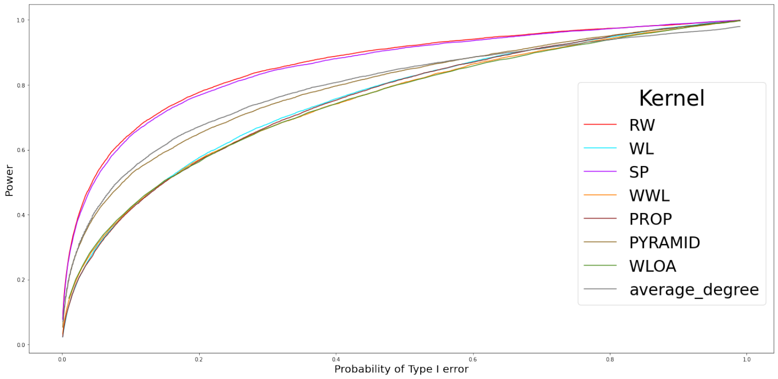

Fig 2. Kernel hypothesis testing performance on the binomial experiment

A remark on this experiment is that the number of samples is really small explaining the low power for $\alpha \approx 0.025$. If we increase the sample size all tests will give high power. For example, when $\alpha = 0.025$ the power is around 0.55, and when the sample size is 100 the power is above 0.8 for $\alpha = 0.025$.

5.2 Scale Free Graphs

Here we will look at how different graph kernels can discriminate between two populations following different power laws. Multiple naturally occurring graphs are said to have scale-free graphs, such as the internet, airline networks, co-author networks, and software dependency graphs.

Definition 5.1 (Scale Free Graphs)**.** *Let p_k denote the probability that a node as degree k, p_k is said to follow a power-law if we can write the relation between p_k and k as:*

$$p_k = Ck^{-\alpha},$$

where C and α are constants. A graph where the degree distribution follows a power law is called a scale-free graph.

It is possible to determine if a graph is a scale-free by determining whether $\log p_k$ and $\log k$ have a linear relationship, that is:

$$\log p_k = \log C - \alpha \log k,$$

where α is determined by, for example, a least-squares. The constant C can be approximated by using:

$$C \approx \frac{1}{\int_{k_{min}}^{\infty} k^{-\alpha}} = (\alpha - 1)k_{min}^{\alpha-1},$$

where $k_{min} > 0$ is an integer determining the lowest possible degree of a node. To sample the scale-free graph we use the configuration model. We begin by generating a degree sequence $\{k_i\}_{i=1}^{|}V|$, then we give each node v_i a total of k_i "stubs" of edges or "half-edges". Note that there are $\sum_i k_i = 2|E|$ stubs un total. Finally, we choose two stubs uniformly and connect them together at random to form an edge. One issue with this model is that the sum $\sum_i k_i$ must be some even number, meaning that we might have to sample from the power-law distribution more than once. Another issue is that this process might generate multi-edges or self-loops. If that happens, we simply remove them. In the following experiment, we generate a sample having 40 graphs with 80 nodes each using $k = 2.1$ and another sample having 40 graphs with 80 nodes using $k = 2.2$. Fig 3 shows the ROC curve for each graph kernel.

1. We can see that the random walk kernel outperforms the other kernels. The reason is that the random walk kernel compares the average degrees. Interestingly, the geometric random walk kernel still outperforms the other kernels even though the degree distributions do not have a second moment.
2. The SP kernel is giving very poor results in this experiment and it may be surprising given its superb performance for the binomial graph experiment. This can be explained if we look at the random variable L denoting the shortest path between two nodes. It has been showed that $\mathbb{E}[L] \sim \log \log |V|$ when $1 \geq \alpha \geq 2$ [5]. This means that the shortest paths in the two samples are the same on average.

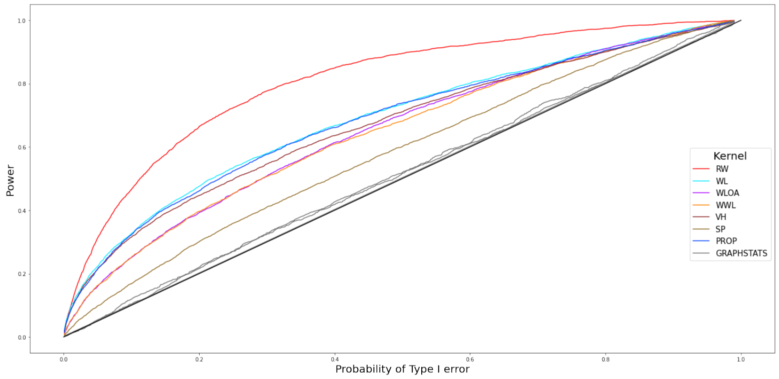

Fig 3. ROC curves for different graph kernels for the Scale Free

5.3 Stochastic Block Model with Different Label Distribution

In this subsection, we will consider graphs that follow a stochastic block model (SBM). A general SBM model is parameterized by a $k \times k$ matrix $\Theta = [\theta_{ab}]$ where θ_{ab} denotes the probability of an edge between nodes in class a and class b. It then follows that each entry in the adjacency matrix A has probability distribution $A_{ij} \sim \text{Bernoulli}(\theta_{c_i c_j})$, $i \neq j$, $i = 1, ..., n$ and $j = i, .., n$, where n is the number of nodes. Here we are not allowing self-connection and we only model the upper triangle as we are assuming an undirected graph.

We will consider a SBM with three blocks. The block matrix is shared between the two samples and is set as:

$$P = \begin{bmatrix} 0.15 & 0.05 & 0.02 \\ 0.05 & 0.25 & 0.07 \\ 0.02 & 0.07 & 0.2 \end{bmatrix},$$

where the entry P_{ij} defines the probability that there is an edge between nodes in block i and block j. The number of nodes in each block is 50, 30, and 35, respectively. The nodes in sample 1 are labeled according to their block, that is, nodes in block 1 are labeled 1. The nodes in sample 2 are labeled similarly but have a small chance of being labeled wrongly. Let b_i denote the block of node i and let $0 < c < 1$, then we define the node label probability as:

$$P(l(v_i) = b_i) = 1 - c$$
$$P(l(v_i) = k) = \frac{c}{2}, \quad \forall k \neq b_i.$$

Fig 4 displays the power as a function of α, the probability of type I error for different graph kernels. We look at the case when the number of graphs in each sample is 60 and when the noise parameter is 0.02. All graph kernels are unnormalized.

- The performance of the WL kernel is similar for each number of WL iterations tried.
- SP gives good performance and the results are indifferent to whether it is normalized or not.
- The propagation kernel performs best when the number of walks is not too high, and $t_{max} = 3$ is best in this case. Also, lower values of w usually outperform the higher ones, that is a value $w = 0.01$ and $w = 0.001$ gives the best performance, but the parameter can not be too low. Again, if $w = 0.001$ then the difference between the AUC of propagation kernels with different steps is lesser.
- The WWL kernel is giving good results. The number of WL iterations does not seem to matter and the discount was tested for the values 0.01, 0.1, 1, and 10 and they all gave similar results. The best parameter values in terms of AUC where $t_{max} = 3$ and $w = 0.01$.
- The Pyramid kernel is giving good results when the level L is low. The best parameters for this experiment were $d = 3$ and $L = 4$. The experiment tested $d = \{1, 2, 3, 4, 6\}$.
- The WLOA kernel is giving good results for all values of the number of wl iterations tested.
- The shortest path version of the deep kernel is giving better performances than the WL type. The number of WL iterations tested where $\{1, 2, 4, 6\}$.
- The label RW kernel gives the best performance when the nr of eigenvalues used is around 10. However, it does not give as good a performance as the best-performing kernels.

Fig 5 summarizes the results. The best-performing kernels in this experiment are the WL-OA, WWL, SP, and the propagation kernel. The vertex histogram is the worst-performing kernel.

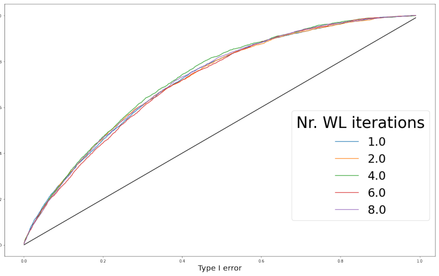

(a) The WL kernel.

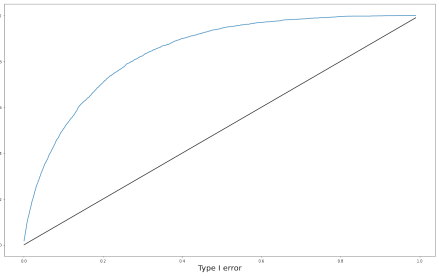

(b) The SP kernel.

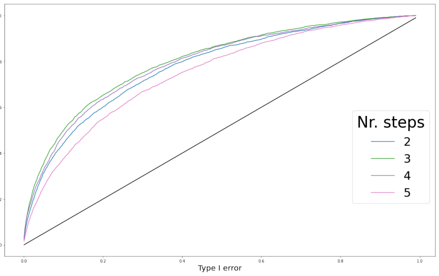

(c) The propagation kernel. The width parameter is set to 0.01.

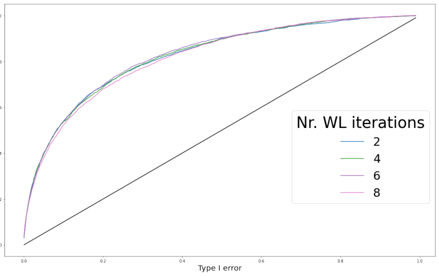

(d) The WWL kernel. The discount is set to 0.1.

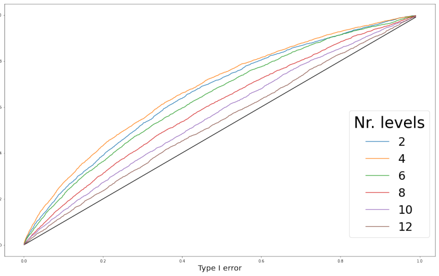

(e) The pyramid kernel. The dimension is set to 3.

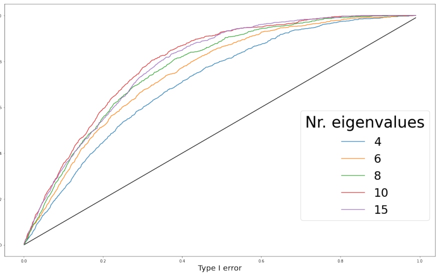

(f) The RW kernel. The discount constant is set to 0.01.

Fig 4. Experiment based on labeled SBM models.

5.4 Stochastic Block Model with Different Block Matricies

Here we will consider graphs from two SBM's with different block matrices. Consider the following block matrix:

$$P = (1-\lambda)\begin{bmatrix}0.08 & 0.01 & 0.01\\ 0.01 & 0.09 & 0.01\\ 0.01 & 0.01 & 0.095\end{bmatrix} + \lambda\begin{bmatrix}0.1 & 0.02 & 0\\ 0.02 & 0 & 0\\ 0 & 0 & 0\end{bmatrix}.$$

Sample 1 is made out of graphs with $\lambda = 0$ while sample 2 contains graphs with a block matrix with a $\lambda \neq 0$. Multiple values of $0 < \lambda < 1$ were tested. The nodes were labeled according to their degree.

From Fig 6 the following can be seen:

- For the WL kernel we can see that the AUC is more or less indifferent to the number of WL iterations.
- The SP kernel shows decent performance. It is better in this case to not label the nodes according to their degree.
- For the WWL kernel we can see that the AUC is more or less indifferent to the number of WL iterations and the discount factor.
- Generally, the propagation kernel performs best with a lower number of steps. However, if the width is around 0.001 then it does not matter what the number of walks is, they all perform very similarly.

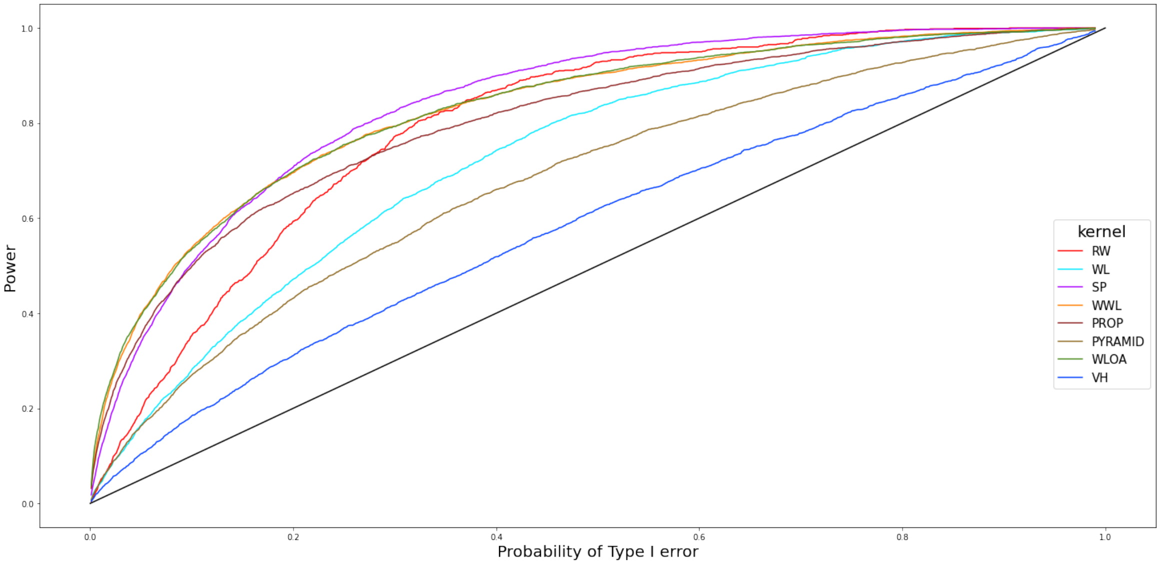

Fig 5. Kernel hypothesis testing performance on the SBM label experiment

- The pyramid kernel has the best performance when the number of levels is around $L = 6$ to $L = 8$. The dimension parameter gives the best performance when set to 2. Here it does not matter if the nodes are labeled according to their degree, the results are the same.
- The WL-OA kernel performance is indifferent to the number of iterations.
- The RW kernel gives a good performance. The ARKU_plus version gives the best performance. The p-step random walk kernel gives very similar results. The number of eigenvalues tested was 2,4 and 6. Surprisingly the ROC curve for the 6 eigenvalues was lower than for 2/4 eigenvalues.

Finally, we will compare all the kernels along with a test based on the average degree on one graph. In Fig 7 we see that the approximated geometric random walk kernel gives the best performance and that the shortest path kernel without node labeling is second. The reason that the RW kernel gives the best performance is perhaps not so surprising as the graph distribution is again specified by average degrees.

Again the power is very low for low type I errors, however, as the sample size is increased the power approaches 1.

5.5 Signed Networks

Signed networks and balance theory have been studied in a diverse range of contexts in both the natural and social sciences. It is therefore important to test how well graph kernels perform in a labeled/signed edge environment. The balance ratio is defined as

$$b = \frac{\text{Nr. balanced triangles}}{\text{Total nr. of triangles}}.$$

A sample of signed graphs is simulated in the following way:

- A balance target, b^* for each graph is set
- Each graph in the sample is from the configuration model where the degrees are generated from a scale-free degree with parameter $\alpha = 2.1$, that is $p(k) \sim k^{-\alpha}$
- The edges are labeled randomly with 1 or -1 uniformly
- For each graph the balance ratio b is calculated. If $b < b^*$ then a random triangle is balanced, and this procedure loops until $b \sim b^*$ or if $b > b^*$. If $b > b^*$ the reverse procedure is performed

Note that the balance target is rarely achieved but with more nodes, more triangles appear and the balance ratio will be close to the target. In this experiment sample 1 has balance target $b_1^* = 0.7$ and and sample 2 has $b_2^* = 0.5$. Both graphs have 200 nodes and there are 60 graphs in each sample.

From Fig 8 we can see that the proposed graph kernels can distinguish between signed networks with different balance ratio.

From Fig 9 we can see that in this case, it is best to label the nodes by concatenating the edge labels and the use a node label kernel. The RW kernel is giving worse performance than the VH

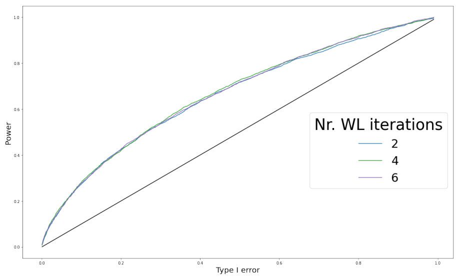

(a) The WL kernel.

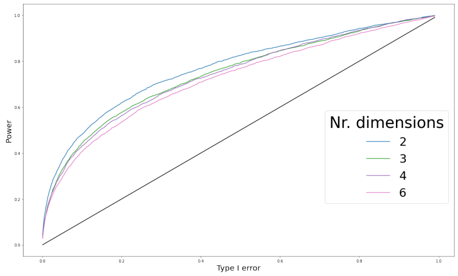

(b) The pyramid kernel. The number of levels is set to 8.

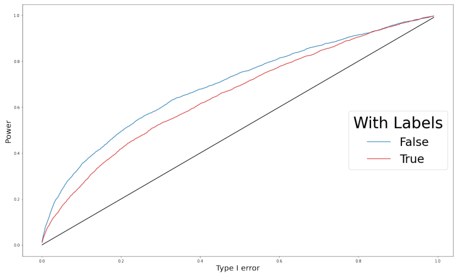

(c) The SP kernel.

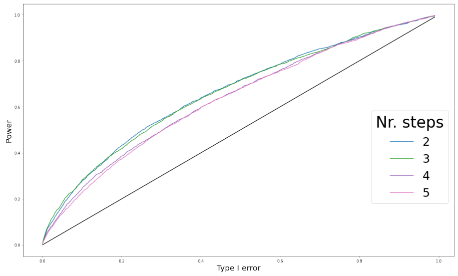

(d) The propagation kernel. The discoint factor is 0.01.

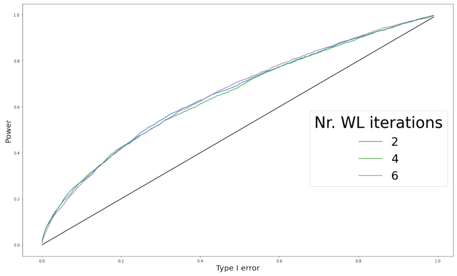

(e) The wloa kernel

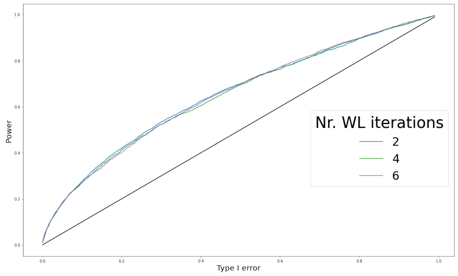

(f) The wwl kernel. The discount is set to 0.01.

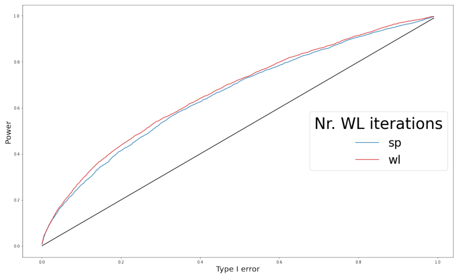

(g) The DK kernel. The wl type uses 4 WL iterations.

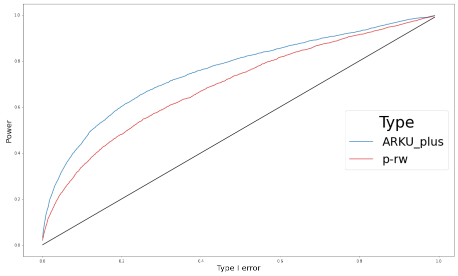

(h) The RW kernel. The discount factor used was 0.001, the number of eigenvalues used is 4 and the p-step random walk kernel used 4 steps.

Fig 6. ROC curves for different graph kernel MMD tests for the SBM topology experiment.

kernel. The experiment demonstrates that the RW kernel does not perform well when measuring balance. To give the RW change for redemption we consider an additional study. We will consider the same power law samples from before, but now we randomly assign signs to the edges. For sample one, the edges can take the label +1 with a probability of 0.6, while the graphs in sample two can have

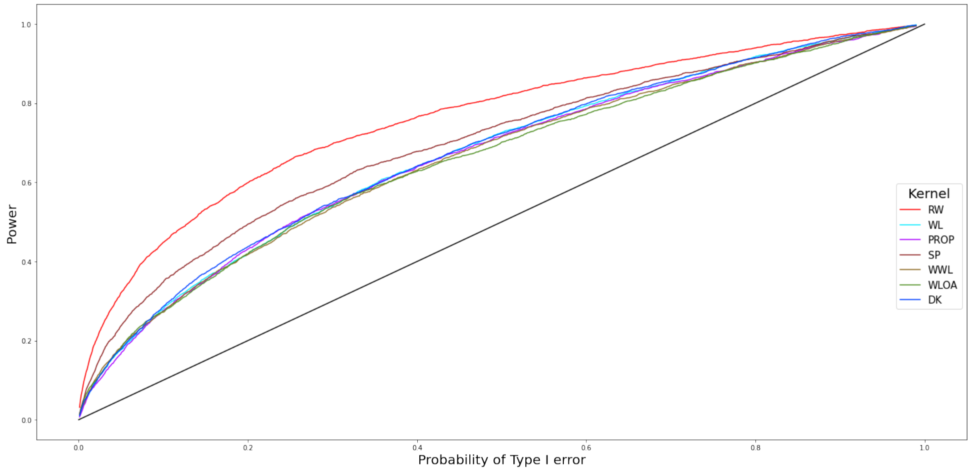

Fig 7. Kernel hypothesis testing performance on the SBM block matrix experiment

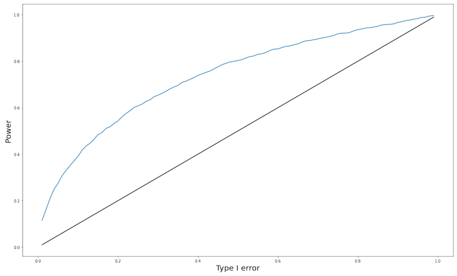

(a) The edge histogram kernel

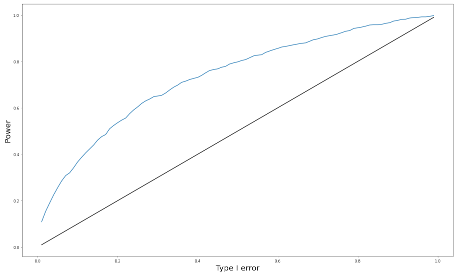

(b) The vertex histogram kernel

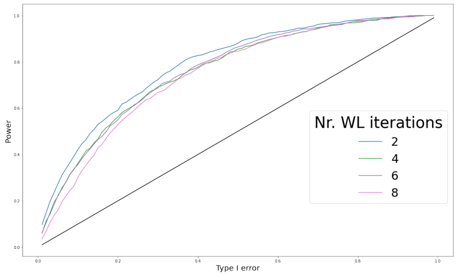

(c) The WLOA kernel

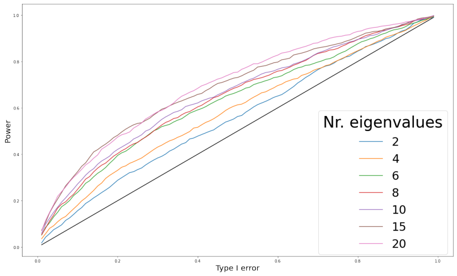

(d) The RW kernel. The discount was set to 0.001.

Fig 8. The signed network experiment

their edges labeled +1 with a probability of 0.55. Fig 10 demonstrates the result when there are 60 graphs in each sample and when each graph has 50 vertices. Now the results have completely changed! The RW kernel is the best one (along with the EH kernel) while the WLOA gives the best performance.

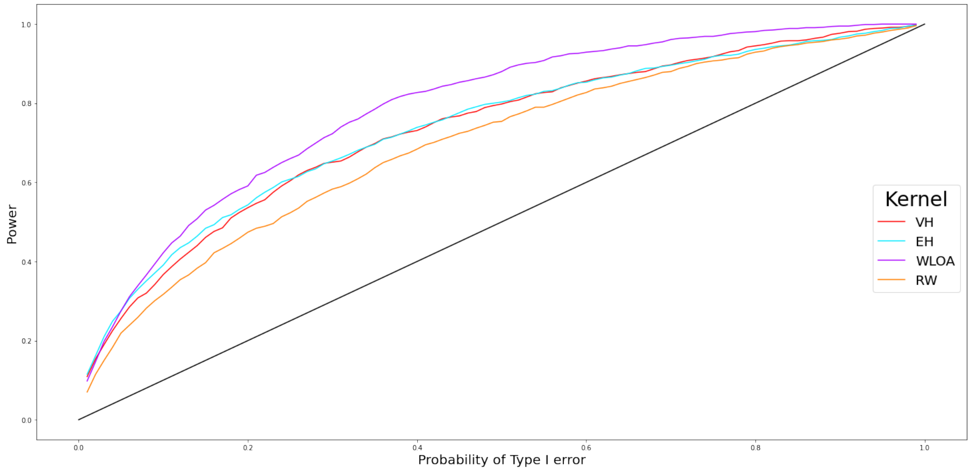

Fig 9. Kernel hypothesis testing performance for the signed network experiment.

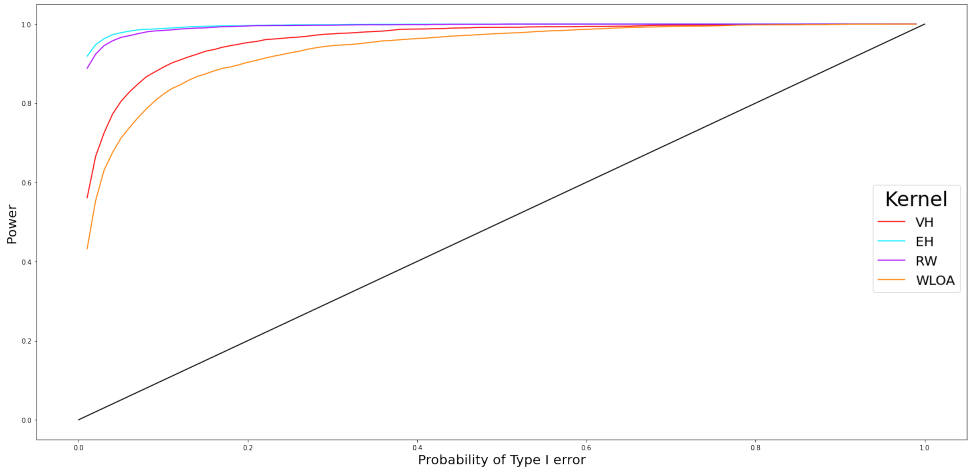

Fig 10. Kernel hypothesis testing performance for the signed network experiment.

5.6 Robust Graph Testing

Consider two graph samples $\{G_i\}_{i=1}^n$ and $\{G'_i\}_{i=1}^m$ coming from the same distribution. If the samples are corrupted meaning that some of the graphs within the samples are outliers then the unbiased or biased MMD might reject the test as they are not robust to outliers. The probability of Type I error of course depends on the number of corrupted samples but here we demonstrate the robustness of the MONK estimator. We will consider two experiments, the first considers the case when $\{G_i\}_{i=1}^{n-5}, \{G'_i\}_{i=1}^{n-5} \sim B\big(\binom{50}{2}, p\big)$ and $\{G_i\}_{i=n-5+1}^{n} \sim B\big(\binom{50}{2}, p'\big)$ with $p = 4/49$ and $p' = 7/49$ where $n = 100$ and each the p - value is simulated 1000 times.

In Fig 11a we can see the ROC curve for different MMD estimators. Note in this case H_0 is true meaning that we want the curve to be as close to the line $y = x$ as possible (black line). As the MONK partition, Q increases we see that the proportion rejected lowers, Also the power for MMD_u and MMd_P at $\alpha = 0.05$ is around 0.56, while it is 0.33 and 0.3 for $Q = 5$ and $Q = 11$, respectively.

In the second experiment, we again generate binomial graphs using $\{G_i\}_{i=1}^n, \{G'_i\}_{i=1}^n \sim B\big(\binom{50}{2}, p\big)$ but for $\{G_i\}_{i=1}^{n-5}$ and $\{G'_i\}_{i=1}^n, \{G'_i\}_{i=1}^n$ we generate node attributes using $N(0, 0.1)$ while for $\{G_i\}_{i=n-5+1}^n$ we generate node attributes using $N(10, 0.1)$. In Fig 11a we can see the ROC curve for different MMD estimators. Again we see that the $MONK$ estimator gives lower rejection rate and here the power for MMD_u and MMd_b at $\alpha = 0.05$ is around 0.15, while it is 0.1, 0.08 and 0.09 for $Q = 5$, $Q = 9$ and $Q = 11$, respectively.

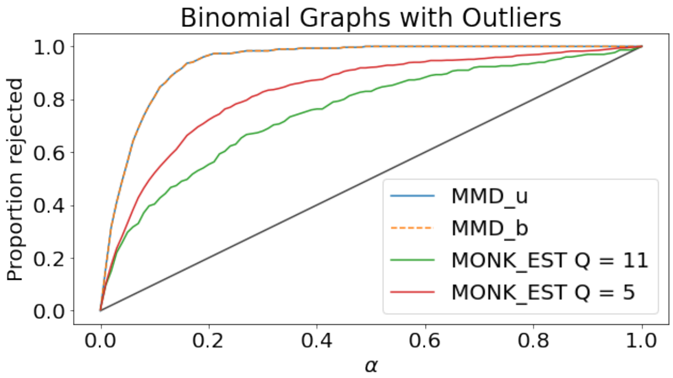

(a) ROC curve for binomial graphs with outliers

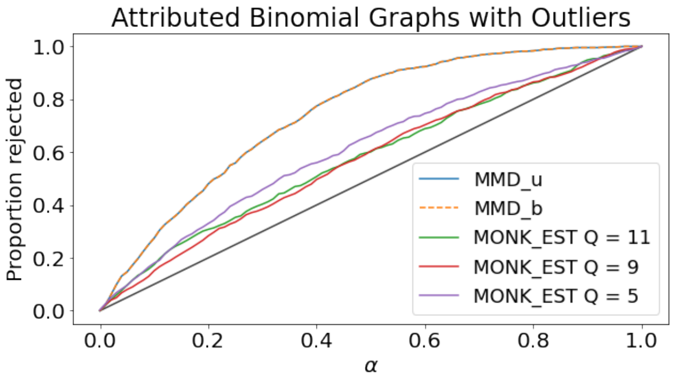

(b) ROC curve for binomial graphs with outliers

6 Portfolio Metrics

The Sharpe ratio is a risk-adjusted measurement for the performance of a portfolio and is defined as:

$$\mathrm{SR} = \frac{\mathbb{E}[R_p - R_f]}{\sqrt{\mathbb{V}[R_p]}}.$$

The maximum drawdown (MDD) is the maximum observed loss from a peak to a trough of a portfolio before a new peak is attained:

$$\min_t \left(\frac{\tilde{p}_t}{\max_{t' \le t} (\tilde{p}_{t'})} - 1 \right),$$

where $\tilde{p}_t = \frac{p_t}{p_0}$ and p_t is the price at time t. The omega function [6] was introduced as a risk measure to capture higher moment information and incorporate sensitivity to return levels. It captures higher moment information in the returns distribution and also incorporates sensitivity to return levels. Consider a return level r and define the expected gain and loss relative to the threshold as $g = \mathbb{E}[R|R \ge r] - r$ and $l = r - \mathbb{E}[R|R \le r]$ respectively. The values are then weighted with the appropriate probabilities for a meaningful comparison with results in the following ratio $\frac{g(1-F(r)}{lF(r)}$ where F is the cumulative distribution function for the returns. This ratio only considered one particular gain and loss possibility. To account for more possibilities the ratio is generalized to infinitesimal return levels which gives rise to the Omega function:

$$\Omega(r) = \frac{\int_r^\infty (1 - F(x)dx}{\int_{-\infty}^r F(x)dx}.$$

The main advantage of the omega function is that it provides an appealing and easily calculated risk measure that provides a full characterization of the risk-reward characteristics of the distribution by incorporating the beneficial impact of gains as well as the detrimental effect of losses, relative to any individual's loss threshold. Furthermore, Omega may be used to rank manager performance, without the need to introduce utility functions [6]. We use the empirical distribution and the python package Scipy [7], namely the function *integrate.quad*, to approximate the integral

The Sortino ratio [8] is a variation of the Sharpe ratio that differentiates harmful volatility from total overall volatility by using the asset's standard deviation of negative portfolio returns and is defined as:

$$\mathrm{ST} = \frac{\mathbb{E}[R_p - R_f]}{\sigma_d},$$

where $\sigma_d = \int_{-\infty}^{\mathbb{E}[R_f]} (r - \mathbb{E}[R_f])^2 f(r) dr$ and $f(r)$ is the distribution of the returns. We take $\mathbb{E}[R_f] = 0$ and use the following approximation

$$\hat{\sigma}_d = \frac{1}{n} \sum_{i=1}^n \mathbf{1}[r_i < 0] r_i^2.$$

The portfolio beta β_p measures single index CAPM gradient of a portfolio against the S&P500 index return:

$$\beta_p = \frac{\mathrm{cov}[R_p, R^{\mathrm{S\&P500}}]}{\mathrm{Var}[R^{\mathrm{S\&P500}}]}.$$

The Treynor coefficient measures the portfolios index adjusted Sharpe-type ratio:

$$\text{TR} = \frac{\mathbb{E}[R_p - R_f]}{\beta_p}.$$

7 PCA Analysis

PCA was performed on the following data:

$$x_{i,t} = \frac{\sum_{k=(t-20)}^{t} z_{i,k}^{(g)} - z_{i,k}^{(b)}}{20},$$
$$\tilde{x}_{i,t} = \frac{x_{i,t} - \hat{\mu}_{x_i}}{\hat{\sigma}_{x_i}},$$

where t is a timepoint where a rejection decision is made, $z_{i,k}^{(g)}$ is a portfolio metric value for one particular portfolio construction for the good ESG portfolio while $z_{i,k}^{(b)}$ is defined analogously but for the poor ESG portfolio. We then scale each feature and create $\tilde{x}_{i,t}$ where $\hat{\mu}_{x_i}$ and $\hat{\sigma}_{x_i}$ are the estimated mean and standard deviation of x_i respectively. Note that we take the past 20 observations from time point t. The label y_t is the rejection decision according to the MONK estimator at time t. Figs 12, 13 and 14 shows a PCA biplot for the global portfolio and all sectors, for different portfolio constructions.

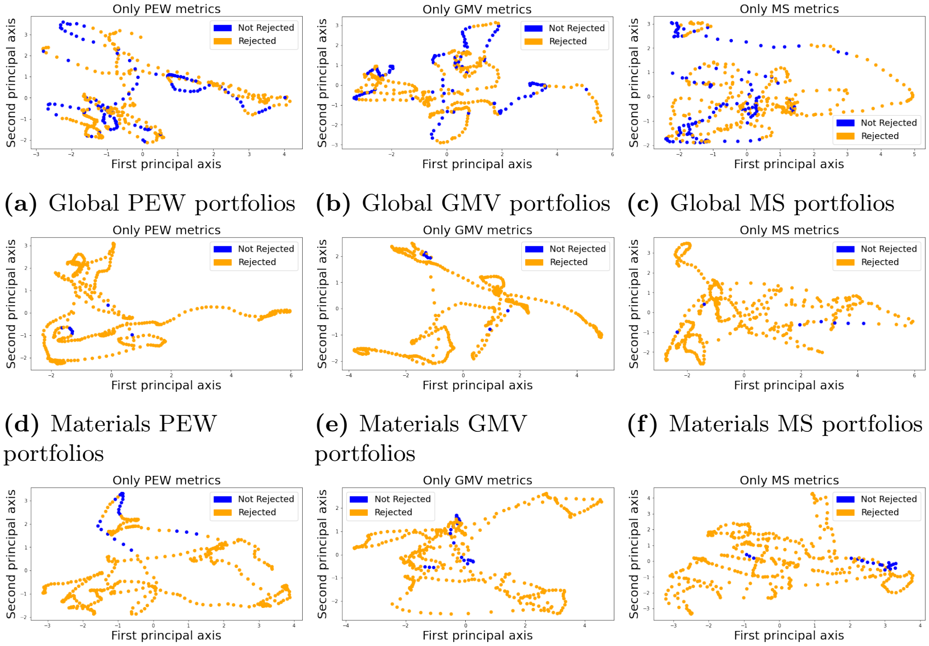

(a) Global PEW portfolios **(b)** Global GMV portfolios **(c)** Global MS portfolios

(d) Materials PEW portfolios **(e)** Materials GMV portfolios **(f)** Materials MS portfolios

(g) Utilities PEW portfolios **(h)** Utilities GMV portfolios **(i)** Utilities MS portfolios

Fig 12. PCA biplots. The orange dots indicate time points when a rejection was made while the orange dots indicate no rejection. Only the labels change between each row.

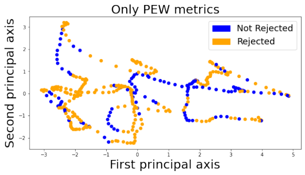

(a) Industrials PEW portfolios

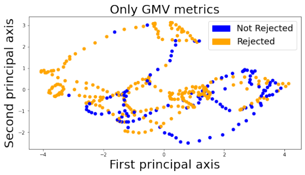

(b) Industrials GMV portfolios

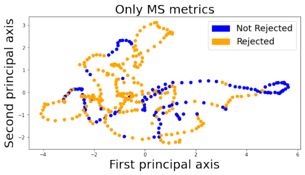

(c) Industrials MS portfolios

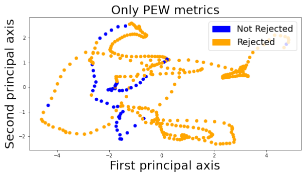

(d) Energy PEW portfolios

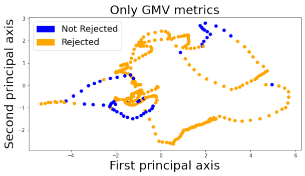

(e) Energy GMV portfolios

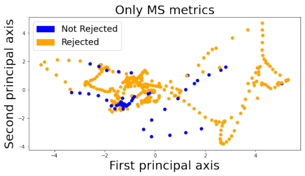

(f) Energy MS portfolios

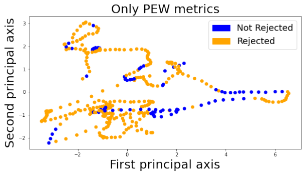

(g) Communication PEW portfolios

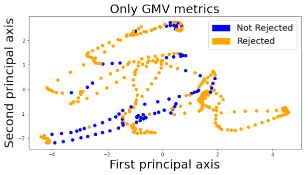

(h) Communication GMV portfolios

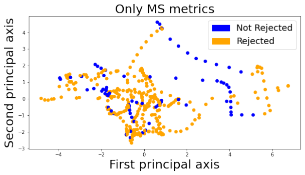

(i) Communication MS portfolios

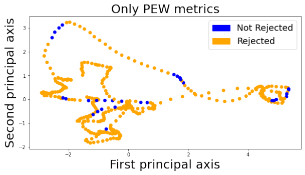

(j) Cyclical PEW portfolios

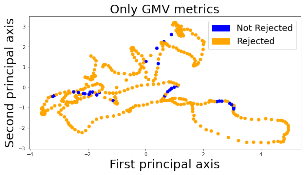

(k) Cyclical GMV portfolios

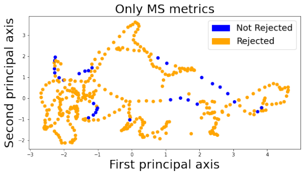

(l) Cyclical MS portfolios

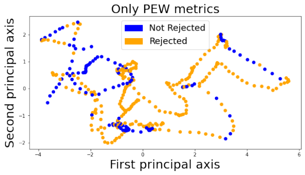

(m) Defensive PEW portfolios

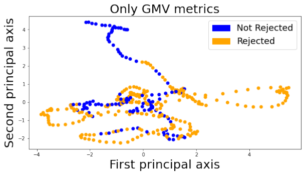

(n) Defensive GMV portfolios

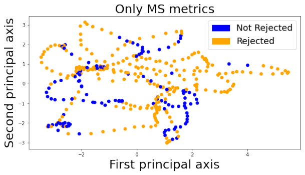

(o) Defensive MS portfolios

Fig 13. PCA biplots. The orange dots indicate timepoints when a rejection was made while the orange dots indicate no rejection. Only the labels change between each row.

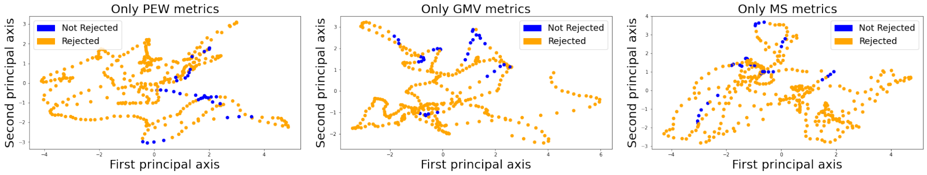

(a) Estate PEW portfolios **(b)** Estate GMV portfolios **(c)** Estate MS portfolios

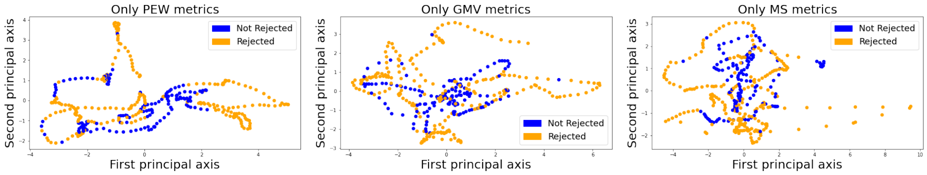

(d) Technology PEW portfolios **(e)** Technology GMV portfolios **(f)** Technology MS portfolios

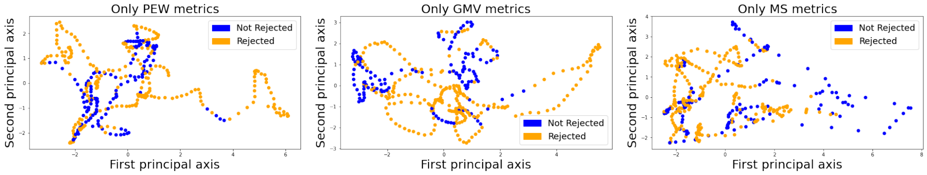

(g) Healthcare PEW portfolios **(h)** Healthcare GMV portfolios **(i)** Healthcare MS portfolios

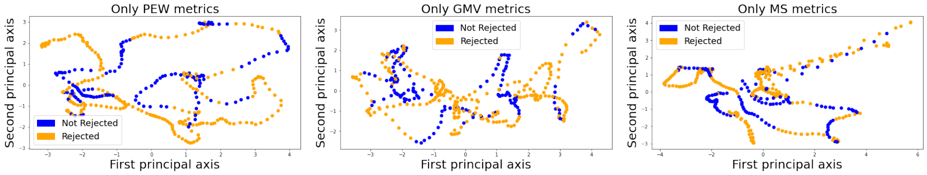

(j) Financial PEW portfolios **(k)** Financial GMV portfolios **(l)** Financial MS portfolios

Fig 14. PCA biplots. The orange dots indicate timepoints when a rejection was made while the orange dots indicate no rejection.

8 SVM results

Table 1 show the AUC using a SVM classification on MONK MMD rejections and the portfolio metrics difference features.

Table 1. AUC of the SVM trying to classify a MONK non rejection when the input data where portfolio metrics. The AUC and its 95% confidence interval were estimated using a 3 fold CV. The column all metrics means that **x** contains all metrics for each portfolio optimization while the columns PEW, GMV and MS metrics mean that the logistic regression input **x** contained only metrics from the specific portfolio optimization mentioned in the column header.

	Kernel\ AUC	All metrics	PEW metrics	GMV metrics	MS metrics
	RW	0.92±0.03	0.87±0.06	0.89±0.04	0.89±0.04
	RW attr.	0.97±0.03	0.96±0.03	0.93±0.1	0.96±0.06
Global	prop.	0.85 ± 0.11	0.82±0.12	0.84± 0.05	0.83± 0.16
	SP	0.88 ± 0.06	0.82±0.12	0.82± 0.11	0.87± 0.06
	WL	0.87 ± 0.1	0.89±0.09	0.83± 0.06	0.85± 0.17
	RW	0.91±0.12	0.82±0.26	0.95±0.04	0.89±0.2
	RW attr.	0.98±0.03	0.84±0.2	0.84±0.14	0.84±0.14
Materials	prop.	0.86 ± 0.01	0.87±0.05	0.83± 0.06	0.81± 0.14
	SP	0.9 ± 0.09	0.91±0.08	0.88± 0.08	0.88± 0.1
	WL	0.97 ± 0.01	0.96±0.01	0.95± 0.02	0.95± 0.02
	RW	1±0.004	0.99±0.008	0.99±0.014	1±0.002
	RW attr.	0.97±0.06	0.95±0.08	0.98±0.04	0.91±0.01
Utilities	prop.	0.93 ± 0.03	0.92±0.02	0.93± 0.02	0.93± 0.04
	SP	0.98 ± 0.01	0.98 ± 0.002	0.97± 0.01	0.98± 0.01
	WL	0.93 ± 0.04	0.91±0.04	0.91± 0.05	0.92± 0.04
	RW	0.91±0.05	0.89±0.04	0.89±0.04	0.89±0.04
	RW attr.	0.95±0.07	0.96±0.02	0.94±0.04	0.96±0.04
Industrials	prop.	0.94 ± 0.05	0.91±0.08	0.93± 0.05	0.94± 0.06
	SP	0.92 ± 0.01	0.92 ± 0.05	0.89± 0.005	0.92± 0.02
	WL	0.84 ± 0.03	0.82 ± 0.03	0.81± 0.08	0.8± 0.04
	RW	0.97±0.02	0.98±0.004	0.97±0.02	0.96±0.04
	RW attr.	0.98±0.002	0.97±0.02	0.98±0.01	0.95±0.02
Energy	prop.	0.96 ± 0.03	0.97±0.02	0.96± 0.03	0.94± 0.07
	SP	0.84 ± 0.08	0.82±0.07	0.77± 0.07	0.78± 0.08
	WL	0.9 ± 0.07	0.9±0.06	0.91± 0.08	0.85± 0.07
	RW	0.91±0.02	0.91±0.03	0.91±0.06	0.9±0.02
	RW attr.	0.95±0.01	0.92±0.05	0.93±0.02	0.91±0.02
Communication	prop.	0.87±0.02	0.86±0.07	0.87±0.05	0.84±0.06
	SP	0.9±0.07	0.86±0.09	0.78±0.09	0.8±0.04
	WL	0.91±0.05	0.9±0.07	0.87±0.03	0.85±0.05
	RW	0.97±0.01	0.96±0.02	0.91±0.02	0.93±0.11
	RW attr.	0.97±0.07	0.9±0.24	0.94±0.14	0.83±0.44
Cyclical	prop.	0.95±0.02	0.95±0.01	0.92±0.04	0.95±0.01
	SP	0.94±0.06	0.94±0.02	0.92±0.05	0.95±0.06
	WL	0.85±0.04	0.81±0.05	0.84±0.02	0.81±0.07
	RW	0.9±0.05	0.88±0.06	0.9±0.01	0.89±0.07
	RW attr.	0.95±0.05	0.8±0.1	0.9±0.1	0.93±0.06
Defensive	prop.	0.9±0.03	0.9±0.01	0.88±0.05	0.86±0.06
	SP	0.9±0.02	0.9±0.05	0.9±0.01	0.9±0.03
	WL	0.86±0.09	0.84±0.09	0.87±0.09	0.86±0.08
	RW	0.97±0.03	0.97±0.04	0.97±0.02	0.97±0.03
	RW attr.	0.89±0.02	0.87±0.03	0.86±0.01	0.87±0.04
Estate	prop.	0.95±0.05	0.94±0.06	0.94±0.05	0.96±0.03
	SP	0.89±0.03	0.86±0.05	0.89±0.01	0.87±0.004
	WL	0.87±0.08	0.86±0.1	0.89±0.06	0.85±0.08
	RW	0.89±0.05	0.89±0.05	0.87±0.09	0.9±0.06
	RW attr.	0.95±0.04	0.93±0.03	0.95±0.03	0.95±0.06
Technology	prop.	0.9±0.05	0.92±0.04	0.91±0.04	0.9±0.06
	SP	0.91±0.01	0.91±0.04	0.9±0.01	0.9±0.05
	WL	0.83±0.05	0.8±0.08	0.83±0.04	0.84±0.08
	RW	0.95±0.01	0.93±0.01	0.93±0.03	0.93±0.01
	RW attr.	0.96±0.05	0.95±0.03	0.96±0.05	0.95±0.07
Healthcare	prop.	0.96 ± 0.06	0.95±0.03	0.96± 0.01	0.94± 0.02
	SP	0.91 ± 0.02	0.91±0.02	0.85± 0.04	0.76± 0.11
	WL	0.86 ± 0.11	0.87±0.09	0.86± 0.15	0.89± 0.11
	RW	0.95±0.02	0.93±0.06	0.91±0.07	0.94±0.03
	RW attr.	0.97±0.01	0.94±0.01	0.97±0.01	0.97±0.04
Financial	prop.	0.95 ± 0.05	0.93±0.06	0.94± 0.05	0.92± 0.08
	SP	0.88 ± 0.04	0.87±0.03	0.89± 0.04	0.88± 0.08
	WL	0.9 ± 0.04	0.9±0.04	0.88± 0.07	0.89± 0.05

9 Rejection Rate per Portfolio Type and Sector

Tables 2 and 3 show the results for the aggregated proportion metric $\mathrm{P}_{\mathrm{rejected}}$ and the individual proportion metric $\mathrm{P}_{\mathrm{rejected},m}$.

Table 2. The occurrence ratio when the good ESG portfolio gave better performances for each portfolio type for each test decision.

		PEW		GMV		MS	
	Kernel\MMD decision	Rejected	Not Rejected	Rejected	Not Rejected	Rejected	Not Rejected
Global	RW	0.67	0.75	0.57	0.53	0.4	0.41
	RW attr.	0.7	0.68	0.61	0.47	0.41	0.4
	Prop.	0.7	0.67	0.56	0.57	0.39	0.47
	SP	0.7	0.66	0.48	0.57	0.41	0.33
	WL	0.7	0.68	0.56	0.55	0.4	0.44
Materials	RW	0.79	0.86	0.74	0.86	0.62	0.79
	RW attr.	0.79	0.92	0.73	0.92	0.61	0.88
	Prop.	0.79	0.87	0.74	0.74	0.62	0.6
	SP	0.8	0.83	0.73	0.85	0.6	0.72
	WL	0.84	0.75	0.73	0.75	0.65	0.58
Utilities	RW	0.46	0.65	0.5	0.56	0.36	0.7
	RW attr.	0.47	0.54	0.5	0.5	0.37	0.55
	Prop.	0.54	0.42	0.55	0.48	0.45	0.34
	SP	0.46	0.66	0.5	0.55	0.37	0.55
	WL	0.46	0.51	0.49	0.56	0.39	0.37
Industrials	RW	0.79	0.77	0.76	0.7	0.68	0.61
	RW attr.	0.79	0.75	0.75	0.7	0.67	0.63
	Prop.	0.76	0.8	0.76	0.71	0.66	0.66
	SP	0.8	0.74	0.76	0.71	0.7	0.59
	WL	0.8	0.75	0.77	0.7	0.72	0.58
Energy	RW	0.65	0.92	0.44	0.61	0.59	0.68
	RW attr.	0.63	0.85	0.41	0.6	0.59	0.63
	Prop.	0.64	0.79	0.42	0.56	0.6	0.61
	SP	0.68	0.79	0.46	0.55	0.6	0.68
	WL	0.68	0.7	0.49	0.42	0.64	0.54
Communication	RW	0.65	0.44	0.53	0.4	0.55	0.5
	RW attr.	0.6	0.72	0.49	0.59	0.53	0.68
	Prop.	0.56	0.77	0.48	0.57	0.5	0.66
	SP	0.61	0.59	0.5	0.45	0.54	0.53
	WL	0.52	0.71	0.46	0.55	0.46	0.62
Cyclical	RW	0.33	0.25	0.37	0.38	0.31	0.31
	RW attr.	0.32	0.3	0.37	0.24	0.32	0.2
	Prop.	0.31	0.36	0.35	0.43	0.31	0.29
	SP	0.35	0.15	0.39	0.25	0.32	0.23
	WL	0.33	0.32	0.338	0.36	0.33	0.3
Defensive	RW	0.57	0.72	0.57	0.7	0.55	0.64
	RW attr.	0.63	0.44	0.64	0.42	0.61	0.28
	Prop.	0.67	0.52	0.65	0.56	0.6	0.53
	SP	0.63	0.56	0.63	0.59	0.59	0.55
	WL	0.6	0.66	0.61	0.64	0.58	0.58
Real Estate	RW	0.14	0.15	0.23	0.37	0.27	0.4
	RW attr.	0.14	0.16	0.25	0.23	0.29	0.26
	Prop.	0.1	0.26	0.23	0.29	0.27	0.32
	SP	0.12	0.18	0.23	0.27	0.28	0.29
	WL	0.14	0.13	0.24	0.25	0.29	0.28
Technology	RW	0.65	0.64	0.39	0.35	0.66	0.62
	RW attr.	0.67	0.58	0.36	0.42	0.65	0.65
	Prop.	0.65	0.65	0.3	0.41	0.67	0.64
	SP	0.61	0.68	0.38	0.37	0.64	0.65
	WL	0.64	0.67	0.37	0.39	0.63	0.7
Healthcare	RW	0.37	0.26	0.53	0.49	0.4	0.34
	RW attr.	0.29	0.45	0.46	0.7	0.32	0.58
	Prop.	0.3	0.41	0.49	0.61	0.35	0.49
	SP	0.34	0.3	0.52	0.51	0.39	0.37
	WL	0.33	0.3	0.54	0.4	0.4	0.31
Financial	RW	0.89	0.88	0.66	0.7	0.75	0.72
	RW attr.	0.89	0.86	0.68	0.66	0.76	0.61
	Prop.	0.9	0.85	0.72	0.6	0.75	0.7
	SP	0.85	0.9	0.64	0.7	0.77	0.72
	SP	0.88	0.89	0.69	0.65	0.75	0.7

Table 3. The occurrence ratio when the good ESG portfolio gave better performances for each portfolio type for each test decision for the RW kernel and the global asset universe. TR = Treynor, S = Sharpe, ST = Sortino, MDD = maximum drawdown.

		PEW		GMV		MS	
	Metric	Rejected	Not Rejected	Rejected	Not Rejected	Rejected	Not Rejected
Global	DRatio	0.77	0.71	0.94	0.82	0.8	0.65
	DVar	0.1	0.34	0.22	0.42	0.22	0.25
	MDD	0.55	0.7	0.31	0.19	0.3	0.17
	Ω	0.76	0.86	0.59	0.55	0.24	0.44
	S	1	1	0.79	0.64	0.84	0.77
	ST	0.76	0.84	0.6	0.56	0.24	0.41
	T	0.77	0.78	0.54	0.54	0.18	0.17
Materials	DRatio	0.69	0.93	0.69	0.93	0.74	0.89
	DVar	0.74	0.93	0.69	0.93	0.72	0.8
	MDD	1	1	0.93	1	0.93	1
	Ω	0.79	0.99	0.72	0.81	0.46	0.72
	S	0.78	0.99	0.71	0.79	0.45	0.72
	ST	0.78	0.99	0.8	0.71	0.47	0.72
	T	0.77	0.98	0.7	0.79	0.53	0.71
Utilities	DRatio	0	0.15	0.26	0.02	0.32	0.5
	DVar	0.01	0.27	0.3	0.41	0.24	0.49
	MDD	0.73	0.86	0.55	0.63	0.47	0.82
	Ω	0.58	0.77	0.68	0.7	0.34	0.76
	S	0.6	0.81	0.58	0.7	0.36	0.75
	ST	0.6	0.81	0.58	0.7	0.35	0.75
	T	0.7	0.85	0.65	0.79	0.44	0.77
Industrials	DRatio	0.46	0.7	0.46	0.67	0.64	0.8
	DVar	0.41	0.49	0.46	0.69	0.6	0.69
	MDD	0.86	0.52	0.73	0.58	0.75	0.66
	Ω	0.94	0.92	0.92	0.77	0.71	0.55
	S	0.94	0.92	0.91	0.78	0.71	0.53
	ST	0.95	0.93	0.91	0.75	0.71	0.54
	T	0.93	0.89	0.92	0.71	0.66	0.54
Energy	DRatio	0.83	0.92	0.83	0.95	0.57	0.72
	DVar	0.82	0.87	0.77	0.89	0.72	0.77
	MDD	0.76	0.98	0.27	0.43	0.53	0.68
	Ω	0.56	0.92	0.32	0.5	0.63	0.71
	S	0.55	0.92	0.32	0.49	0.63	0.73
	ST	0.54	0.92	0.3	0.5	0.62	0.72
	T	0.52	0.9	0.28	0.54	0.53	0.72
Communication	DRatio	0.4	0.4	0.48	0.46	0.68	0.59
	DVar	0.71	0.36	0.53	0.41	0.74	0.57
	MDD	0.58	0.17	0.53	0.27	0.46	0.22
	Ω	0.75	0.56	0.55	0.4	0.46	0.53
	S	0.74	0.54	0.55	0.4	0.46	0.53
	ST	0.74	0.52	0.55	0.4	0.48	0.53
	T	0.68	0.52	0.55	0.41	0.58	0.55
Cyclical	DRatio	0	0.03	0.03	0.04	0.17	0.21
	DVar	0.0	0.0	0.23	0.11	0.22	0.29
	MDD	0.66	0.22	0.47	0.65	0.65	0.5
	Ω	0.39	0.37	0.46	0.45	0.26	0.28
	S	0.39	0.36	0.46	0.46	0.26	0.28
	ST	0.43	0.37	0.47	0.46	0.28	0.46
	T	0.44	0.37	0.49	0.46	0.35	0.32
Defensive	DRatio	0.88	0.9	0.38	0.46	0.3	0.41
	DVar	0.77	0.7	0.32	0.59	0.27	0.47
	MDD	0.4	0.55	0.56	0.5	0.61	0.62
	Ω	0.5	0.69	0.68	0.86	0.68	0.77
	S	0.49	0.69	0.69	0.87	0.7	0.75
	ST	0.5	0.69	0.68	0.87	0.69	0.77
	T	0.46	0.67	0.7	0.89	0.59	0.69
Real Estate	DRatio	0.2	0.6	0.16	0.67	0.32	0.67
	DVar	0.25	0.35	0.24	0.62	0.41	0.72
	MDD	0.07	0	0.34	0.25	0.26	0.58
	Ω	0.11	0.03	0.23	0.28	0.25	0.24
	S	0.1	0.02	0.23	0.28	0.25	0.24
	ST	0.12	0.02	0.24	0.28	0.24	0.28
	T	0.12	0	0.19	0.22	0.17	0.12
Technology	DRatio	0.72	0.79	0.41	0.23	0.62	0.4
	DVar	0.68	0.75	0.5	0.48	0.62	0.48
	MDD	0.66	0.67	0.45	0.5	0.64	0.62
	Ω	0.65	0.6	0.34	0.3	0.76	0.74
	S	0.63	0.57	0.34	0.31	0.69	0.7
	ST	0.63	0.58	0.33	0.32	0.67	0.72
	T	0.6	0.53	0.33	0.32	0.65	0.71
Healthcare	DRatio	0.63	0.55	0.85	0.9	0.51	0.58
	DVar	0.48	0.48	0.69	0.89	0.49	0.53
	MDD	0.42	0.35	0.47	0.34	0.45	0.34
	Ω	0.26	0.12	0.45	0.36	0.34	0.26
	S	0.26	0.11	0.45	0.36	0.36	0.24
	ST	0.26	0.12	0.45	0.36	0.36	0.26
	T	0.24	0.06	0.37	0.23	0.33	0.06
Financial	DRatio	1	1	0.93	0.92	0.86	0.86
	DVar	0.95	0.99	0.68	0.64	0.5	0.68
	MDD	1	1	0.69	0.86	0.95	0.98
	Ω	0.88	0.85	0.6	0.64	0.77	0.66
	S	0.84	0.81	0.59	0.62	0.76	0.65
	ST	0.83	0.8	0.59	0.61	0.76	0.65
	T	0.72	0.68	0.56	0.6	0.62	0.52

References

1. Vishwanathan SVN, Schraudolph NN, Kondor R, Borgwardt KM. Graph Kernels. Journal of Machine Learning Research. 2010;11(40):1201–1242.
2. Kang U, Tong H, Sun J. Fast Random Walk Graph Kernel. In: Proceedings of the 2012 SIAM International Conference on Data Mining. Society for Industrial and Applied Mathematics; 2012.Available from: https://doi.org/10.1137/1.9781611972825.71.
3. Piegorsch WW, Casella G. Erratum: Inverting a Sum of Matrices. SIAM Review. 1990;32(3):470–470.
4. Murphy KP. Machine learning : a probabilistic perspective. Cambridge, Mass. [u.a.]: MIT Press; 2013.
5. Cohen R, Havlin S. Scale-Free Networks Are Ultrasmall. Phys Rev Lett. 2003;90:058701.
6. Keating C, Shadwick W. A Universal Performance Measure. Journal of Performance Measurement. 2002;6.
7. Virtanen P, Gommers R, Oliphant TE, Haberland M, Reddy T, Cournapeau D, et al. SciPy 1.0: Fundamental Algorithms for Scientific Computing in Python. Nature Methods. 2020;17:261–272.
8. Sortino FA, Price LN. Performance Measurement in a Downside Risk Framework. The Journal of Investing. 1994;3:59. doi:10.3905/joi.3.3.59.
